# A Quantitative, High-Throughput Reverse Genetic Screen Reveals Novel Connections between Pre–mRNA Splicing and 5′ and 3′ End Transcript Determinants

**DOI:** 10.1371/journal.pgen.1002530

**Published:** 2012-03-29

**Authors:** Laura-Oana Albulescu, Nevin Sabet, Mohanram Gudipati, Nicholas Stepankiw, Zane J. Bergman, Tim C. Huffaker, Jeffrey A. Pleiss

**Affiliations:** Department of Molecular Biology and Genetics, Cornell University, Ithaca, New York, United States of America; Yale University School of Medicine, United States of America

## Abstract

Here we present the development and implementation of a genome-wide reverse genetic screen in the budding yeast, *Saccharomyces cerevisiae*, that couples high-throughput strain growth, robotic RNA isolation and cDNA synthesis, and quantitative PCR to allow for a robust determination of the level of nearly any cellular RNA in the background of 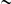
5,500 different mutants. As an initial test of this approach, we sought to identify the full complement of factors that impact pre–mRNA splicing. Increasing lines of evidence suggest a relationship between pre–mRNA splicing and other cellular pathways including chromatin remodeling, transcription, and 3′ end processing, yet in many cases the specific proteins responsible for functionally connecting these pathways remain unclear. Moreover, it is unclear whether all pathways that are coupled to splicing have been identified. As expected, our approach sensitively detects pre–mRNA accumulation in the vast majority of strains containing mutations in known splicing factors. Remarkably, however, several additional candidates were found to cause increases in pre–mRNA levels similar to that seen for canonical splicing mutants, none of which had previously been implicated in the splicing pathway. Instead, several of these factors have been previously implicated to play roles in chromatin remodeling, 3′ end processing, and other novel categories. Further analysis of these factors using splicing-sensitive microarrays confirms that deletion of Bdf1, a factor that links transcription initiation and chromatin remodeling, leads to a global splicing defect, providing evidence for a novel connection between pre–mRNA splicing and this component of the SWR1 complex. By contrast, mutations in 3′ end processing factors such as Cft2 and Yth1 also result in pre–mRNA splicing defects, although only for a subset of transcripts, suggesting that spliceosome assembly in *S. cerevisiae* may more closely resemble mammalian models of exon-definition. More broadly, our work demonstrates the capacity of this approach to identify novel regulators of various cellular RNAs.

## Author Summary

The coding portions of most eukaryotic genes are interrupted by non-coding regions termed introns that must be excised prior to their translation. The excision of introns from precursor messenger RNA (pre–mRNA), is catalyzed by the spliceosome, a large macromolecule composed of both RNA and protein components. Several studies have uncovered connections between pre–mRNA splicing and other RNA processing pathways such as the remodeling of chromatin structure, transcription, and processing events that take place at the 3′ end of the transcript. To date, however, the full complement of factors that function to couple splicing to other processes in the cell remains unknown. Here, we have developed a novel screening methodology in the budding yeast, *Saccharomyces cerevisiae*, that allowed us to individually examine nearly all of the ∼6,000 genes to determine which factors functionally impact splicing. We identified mutations in components that function at either the 5′ or 3′ end of a gene. Most of these components have previously established roles in other aspects of gene expression, including chromatin remodeling and cleavage and polyadenylation processes, and their identification here provides the first evidence for their roles in coupling these pathways.

## Introduction

The coding portions of most eukaryotic genes are interrupted by non-coding introns which must be removed prior to the translation of their messenger RNAs (mRNA). Removal of introns from pre–mRNAs is catalyzed by the spliceosome, a large and dynamic ribonucleoprotein complex comprised of five small nuclear RNAs (snRNAs) and at least 100 proteins [1]. Much of our knowledge about the components that comprise the spliceosome as well as their mechanisms of action has been derived from experiments using the powerful genetic tools available in the budding yeast, *Saccharomyces cerevisiae*. Indeed, although the *RNA2 – RNA11* genes originally identified in Hartwell's forward genetic screen *preceded* the discovery of splicing [2], the mechanistic characterizations of these genes, since renamed *PRP2 – PRP11*, underlie current models of the splicing pathway. Importantly, because the core components of the spliceosome are highly conserved between budding yeast and humans, the mechanistic details derived from work in yeast have been instrumental in understanding mechanisms of pre–mRNA splicing in higher eukaryotes.

The modern view of pre–mRNA splicing acknowledges the integrated role of the spliceosome with several other aspects of RNA processing. Whereas the historical view of splicing envisioned a cascade of temporal events initiated by transcription, followed by polyadenylation, and finalized with splicing and export of mRNAs from the nucleus, it is now clear that these pathways are not independent from one another but rather are functionally coupled. Strong evidence in both yeast and higher eukaryotes demonstrates that recruitment of the spliceosome to intron-containing transcripts occurs co-transcriptionally [3]–[6], mediated at least in part by physical associations between the C-terminal domain (CTD) of RNA polymerase II and the U1 snRNP [7]. A growing body of evidence also indicates that the landscape of chromatin modifications encountered by transcribing polymerase molecules can dictate the activity of the spliceosome at various splice sites. For example, recent work has identified an enrichment of methylated lysine-36 in the histone H3 protein specifically within exonic sequences, suggesting a possible mechanism for facilitating the identification of intron-exon boundaries [8], [9]. Similarly, the rate of transcription by RNA polymerase II, which can be impacted by chromatin marks, has also been shown to be critical for dictating alternative splicing decisions [10]. Furthermore, it is also clear that splicing is coupled to downstream steps in RNA processing. For example, the yeast Ysh1 protein [11], [12], which is the homolog of CPSF73, the mammalian endonuclease required for 3′ end processing, was originally identified as Brr5 in a cold-sensitive screen for mutants defective in pre–mRNA splicing [13]. Consistent with this observation, recent evidence suggests that transcriptional pausing near the 3′ end of genes is a critical component of pre–mRNA splicing efficiency [14]. Despite the increasing evidence of the interconnectivity of these pathways, in many cases the mechanistic details which underlie these functional relationships remain unclear. Our understanding of these mechanistic connections would benefit from a more complete understanding of the complement of factors through which splicing is connected to these cellular processes.

A variety of recent genome-wide approaches have provided important insights into the connections that exist between the spliceosome and other cellular processes. Two powerful approaches, Synthetic Genetic Array (SGA) Analysis [15] and Epistatic MiniArray Profiling (E-MAP) [16], leverage genetic tools available in yeast to systematically generate millions of double-mutant strains and then carefully quantitate their cellular fitness to determine an interaction score for every pair-wise mutation. On the basis of strong positive or negative genetic interaction scores these approaches have been successfully used to infer functional relationships between many cellular pathways, including several with pre–mRNA processing [17], [18]. Simultaneously, improvements in proteomic methodologies have enabled the direct analysis of protein complexes in organisms as diverse as humans and yeast, allowing for an assessment of all of the stably-bound proteins involved in pre–mRNA splicing in many organisms [19], [20]. While the combination of these and other approaches has provided a global picture of many of the cellular factors that influence the splicing pathway, either directly or indirectly, an important question remains about the functional significance of these factors in the splicing of specific transcripts. Indeed, it has long been known that certain transcripts require the activity of unique accessory factors to facilitate their splicing [21]. Moreover, recent work supports the idea that different transcripts can have a greater or lesser dependence upon the activity of core spliceosomal components for their efficient splicing [22], [23].

Here we present the results of a novel approach that complements the genetic and physical approaches of others by allowing for a direct functional assessment of nearly every gene in the *S. cerevisiae* genome in the pre–mRNA splicing process. For this work, we developed automated methods that enabled the isolation of total cellular RNA from about 5500 unique strains, each of which contained a mutation in a single gene, and all of which were examined during exponential growth in liquid medium. Using a high-throughput quantitative PCR (QPCR) assay, the relative cellular level of nearly any RNA can be readily determined in the background of each of these strains. By assessing the levels of several different pre–mRNA species, we were able to identify not only those factors which are necessary for the splicing of many transcripts, but also factors that are specifically required for the splicing of a subset of intron-containing genes. Whereas our study specifically examines the levels of several cellular pre–mRNAs, the approach described herein can be easily adapted to study the level of nearly any RNA molecule of interest under a wide variety of cellular growth conditions.

## Results

### A high-throughput method for measuring cellular levels of specific RNA species

To identify the comprehensive network of cellular factors that lead to a change in splicing efficiency, we developed a high-throughput reverse genetic screen that allowed us to readily assess changes in pre–mRNA levels in the background of ∼5500 *Saccharomyces cerevisiae* strains, each of which contained a mutation in a single gene. The library of strains contained deletions of non-essential genes [24] as well as conditional mutations in essential genes [25], accounting for mutational access to over 93% of known yeast genes. Using a liquid-handling robot, protocols were developed (see Materials and Methods) that allowed for the simultaneous collection of each of these strains under exponential growth conditions in liquid medium in 384-well plates. Total cellular RNA was isolated robotically from each of these strains using a phenol extraction protocol [23] followed by a glass-fiber purification step [26]. After converting this RNA into cDNA using a random-priming strategy, QPCR was used to directly measure the level of a given RNA species within each strain. Because of the inherent variability between the samples in the cell collection, RNA isolation, and cDNA synthesis steps, the levels of six different RNA species were measured in each of the samples in order to calculate a normalization constant. On the basis of this normalization constant, the relative level of virtually any cellular RNA species can be determined in each of the mutant strains.

**Figure 1:**
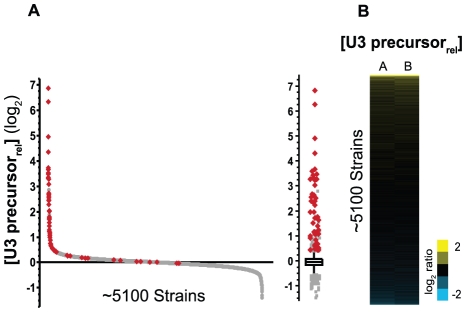
A subset of gene disruptions causes an increase in the unspliced levels of U3 snoRNA. A. The relative level of U3 snoRNA precursor in 5122 strains, ordered from the highest to lowest. Red data points highlight the strains containing mutations in a factor within the GO PROCESS: *RNA Splicing* category (see Figure S1). To the right, a box and outlier plot for the same dataset. B. False color representation of both the A (left) and B (right) biological replicates. The values are ordered from high to low, mirroring the data in (A).

As an initial test of our approach we sought to identify the full complement of factors involved in pre–mRNA splicing by determining the relative levels of unspliced U3 small nucleolar RNA (snoRNA) present in each of the mutant strains. The U3 snoRNA is unique in the *S. cerevisiae* genome in that it is the only known non-coding RNA that is interrupted by a spliceosomal intron [27]. Nevertheless, the U3 transcript has been widely used historically as a splicing reporter, owing to its relatively high basal expression level and the strong accumulation of U3 precursor levels observed in the background of canonical splicing mutants [13], [28], [29]. As shown in Figure 1, the U3 precursor levels are unaffected in the vast majority of the strains examined, with levels varying by less than 1.35-fold from one another for 95% of the strains. Indeed, only ∼200 of the ∼5100 strains that passed our quality filters (see Materials and Methods) showed a change in the relative U3 precursor levels of more than ∼30% from the median value (∼0.35 in log_2_-transformed space), consistent with our expectation that mutations in most genes will have little or no effect on cellular pre–mRNA splicing efficiency. The tight distribution of relative U3 precursor levels seen within this dataset demonstrates the high precision with which these measurements can be made, and suggests a low false discovery rate for our approach.

### Known spliceosomal components dominate the top subset of mutations

To characterize the data generated by this approach we sought to define the biological significance of those strains that showed increased levels of U3 precursor. As an initial analysis, we examined the U3 precursor levels in those strains containing mutations in known splicing genes. Using the GO PROCESS: *RNA Splicing* as a guide [30], a total of 71 strains in our library were classified as containing mutations in canonical splicing factors (Figure S1), of which 68 passed our quality filters for the U3 precursor dataset (see Materials and Methods). A strong overrepresentation of these splicing factors can be seen within the set of strains showing an enrichment of U3 precursor (Figure 1A). Of the 68 strains containing splicing mutations that passed our quality filters: 53 are found within the top 200 strains (p = 9.28E-64, Fisher's exact test); 38 are found in the top 50 strains (p = 1.33E-66); and the top 14 strains all belong to this list (p = 1.27E-27). Taken together, these data argue strongly that the candidates identified by this approach will be characterized by a high true positive discovery rate.

**Figure 2:**
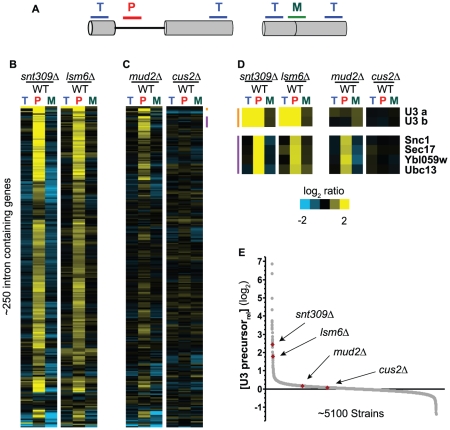
Not all mutations in splicing factors result in defective splicing for all intron-containing genes. A. Splicing-sensitive microarrays contain probes that target precursor (P), mature (M), and total (T) mRNA levels. B. Global splicing profiles of the *snt309Δ* and *lsm6Δ* strains, showing a strong splicing defect for both mutants. C. Global splicing profiles of the *mud2Δ* and *cus2Δ* strains, illustrating different splicing defects for these mutants. Whereas many transcripts show a splicing defect in the *mud2Δ* strain, many continue to be efficiently spliced. Similarly, most intron-containing genes show no splicing defect in the *cus2Δ* strain. D. The behavior of specific transcripts in the different mutant backgrounds. E. Locations within the U3 precursor dataset of the mutant strains for which microarrays are shown in (B) and (C).

By contrast, out of the 68 strains containing mutations in known splicing factors for which we obtained high quality data, 15 failed to show an enrichment of U3 precursor levels in this dataset, suggesting either that mutations in these genes don't cause an increase in U3 precursor levels (true negative), or that our approach incorrectly failed to detect the accumulation of unspliced U3 (false negative). To better resolve these possibilities we chose to more completely examine the global splicing fitness of some of these strains using splicing-sensitive microarrays. For every intron-containing gene in the genome, these custom-designed microarrays contain at least three probes (Figure 2A) that allow us to distinguish between spliced and unspliced isoforms [31]. We used these microarrays to assess the global splicing defects of four mutants: two canonical splicing mutants that showed strong U3 precursor accumulation (*snt309Δ* and *lsm6Δ*, Figure 2B), and two that showed little or no accumulation (*mud2Δ* and *cus2Δ*, Figure 2C). As expected, and consistent with previous work from others [32], the *snt309Δ* and *lsm6Δ* strains demonstrate a broad splicing defect, with most intron-containing genes displaying an increase in precursor levels accompanied by a decrease in the amount of spliced mRNA. By contrast, the global splicing profiles of the *mud2Δ* and *cus2Δ* strains are markedly different. In the *cus2Δ* background, few intron-containing genes display a splicing defect: very little precursor accumulation is observed, and there is little if any detectable loss in mature mRNA. The *mud2Δ* mutation does cause a splicing defect for some intron-containing genes, whereas little change in splicing efficiency is seen for many others. Notably, as seen in Figure 2D, the microarrays of both the *snt309Δ* and *lsm6Δ* strains show a strong accumulation of U3 precursor levels, whereas the *mud2Δ* and *cus2Δ* strains show almost no accumulation, consistent with our QPCR screen results. It is worth noting that in our experience the behavior of the U3 transcript differs from the other intron-containing genes in that every splicing mutation we have examined that causes an increase in the U3 precursor levels also results in an *increase* in the total level of U3; the reason for this apparent discrepancy is currently under investigation. Nevertheless, these microarray data demonstrate that our failure to detect an increase in U3 precursor levels in the *mud2Δ* and *cus2Δ* strains does not represent a failure of the approach, but rather that these are true negative results.

### Assessing transcript-specificity of the mutants

**Figure 3:**
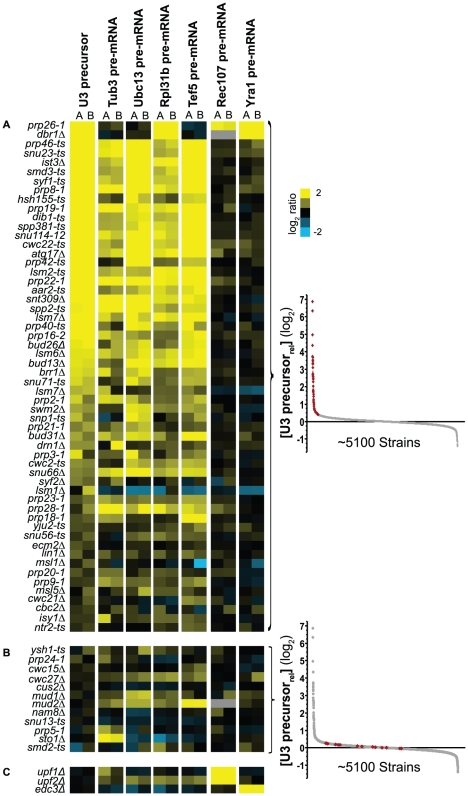
Mutations in most known splicing factors lead to increased precursor levels of canonical splicing substrates. Relative levels of the indicated RNAs are shown in the background of all strains containing mutations in known splicing factors. The biological replicates (A and B) are shown for each RNA. Precursor levels for all transcripts are ordered based on the average expression values of U3 precursor, from high to low values. Gene disruptions are indicated on the left (*-ts* indicates a temperature sensitive allele). Insets to right indicate the location of the data in the U3 precursor dataset. A. Mutations that lead to an increase in the U3 snoRNA. B. Mutations that do not affect U3 precursor levels but may affect the levels of other intron containing genes. C. The increase of Rec107 precursor levels seen in the *ufp1Δ* and *upf2Δ* backgrounds, and the increase of Yra1 precursor levels seen in the *edc3Δ* background are consistent with their well-characterized degradation pathways.

To better assess the total complement of genes that can impact the splicing of any precursor transcript, we chose to expand our analysis by measuring the precursor levels of several additional intron-containing genes. We chose to examine four ‘canonical’ intron-containing genes (*RPL31B*, *UBC13*, *TUB3* and *TEF5*) that vary in terms of intron size, transcriptional frequency, biological function, and the presence or absence of an intron-encoded snoRNA. In spite of these differences, these transcripts are similar to one another in so much as they each contain splice site and branch point sequences that conform to consensus sequences. In addition to these four genes, we chose to examine two intron-containing genes (*YRA1* and *REC107/MER2*) that are known to be poorly spliced under standard growth conditions [21], [33], [34]; as such, we expected the behavior of these two transcripts to be distinct from the efficiently spliced transcripts. For all six of these genes, the precursor levels were measured in all ∼5500 strains. As an initial analysis of this data set, we considered the behavior of the 71 strains containing mutations in spliceosomal components (Figure 3). As expected, precursor accumulation can be detected for each of the canonical intron-containing transcripts in the background of nearly all of the splicing mutations. While all four canonical precursors accumulate in the *mud2Δ* background, consistent with our microarray data, no precursor accumulation is detected for any of these transcripts in the *cus2Δ* strain (Figure 3B). In addition, several of the splicing mutants that failed to cause an increase in the U3 precursor levels do cause a splicing defect for these other transcripts. Importantly, the behavior of the Rec107 and Yra1 pre–mRNAs within this subset of strains differs significantly from that seen for the canonically spliced transcripts. Splicing of the Rec107 pre–mRNA shows a strong accumulation in the *upf1Δ* and *upf2Δ* strains (Figure 3C), consistent with its known degradation via the nonsense-mediated decay pathway [35]. Because the Rec107 pre–mRNA does not engage the spliceosome during vegetative growth [21], no precursor accumulation is expected in strains containing spliceosomal mutations. Likewise, the Yra1 pre–mRNA shows a strong accumulation in the *edc3Δ* strain [34], consistent with its previously characterized cytoplasmic degradation pathway. The failure to detect Yra1 pre–mRNA accumulation in strains containing spliceosomal mutations presumably reflects the inherently high levels of unspliced Yra1 transcript present in a wild type cell. Taken together, these data strongly support the capacity of this approach to successfully identify mutations that impact pre–mRNA splicing with low false positive and false negative rates of discovery.

### Global splicing efficiency is impacted by many cellular mutations

**Figure 4:**
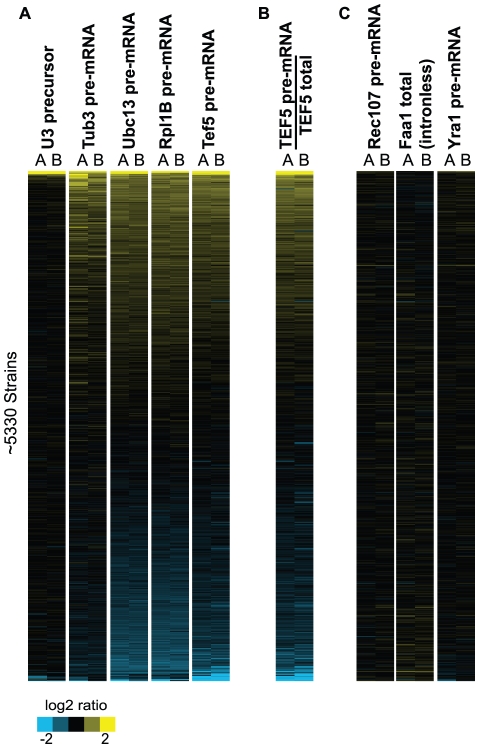
Increases in precursor levels correlate with a decrease in splicing efficiency. A. Relative levels of the indicated RNAs are shown in the background of 5334 strains. The data are ordered from high to low values based on a composite rank order, determined on the basis of the expression levels of the five canonical splicing substrates. B. The relative splicing efficiency of the Tef5 transcript in each of these strains, calculated as the relative Tef5 precursor levels divided by the relative Tef5 total RNA levels. C. The relative levels of the low abundance Rec107 pre–mRNA and Faa1 transcripts are shown along with the inefficiently spliced Yra1 pre–mRNA, none of which shows a similar pattern to the splicing defects seen in (A).

To expand our analysis beyond previously characterized splicing factors, we sought to identify novel mutations that caused an increase in precursor levels in most, if not all, of our canonical intron-containing genes. By determining the rank order of precursor accumulation in each strain for each of the five canonical splicing substrates (U3, Rpl31b, Tef5, Tub3, and Ubc13 precursors), a composite rank order of each strain was calculated as the average of these independent measurements (Figure 4). Remarkably, while the majority of the mutations examined cause little or no change in precursor levels of these four transcripts, the subset of mutations which do cause detectable increases in precursor levels is larger for some of the coding mRNAs than was seen for U3. Interestingly, although there is variation in the number of strains that cause pre–mRNA accumulation of the different transcripts, with Tub3<Tef5<Ubc13∼Rpl31b, strong overlap can nevertheless be identified across the four transcripts. For example, the majority of the strains that cause an increase in the Tub3 pre–mRNA also display an increase in the pre–mRNA levels of the other three transcripts. By contrast, many strains cause a strong accumulation of the Ubc13 and Rpl31b pre–mRNAs without causing a significant change in the Tub3 or Tef5 pre–mRNA levels.

Because the absolute levels of the Rpl31b, Tef5, Tub3 and Ubc13 pre–mRNAs are significantly lower than the U3 precursor levels in most strain backgrounds (Table S1), we considered the possibility that these results reflected a technical artifact associated with measuring the cellular levels of low abundance RNA species in certain strain backgrounds. Importantly, however, the relative levels of the Rec107 pre–mRNA, whose normal cellular level is similar to these other pre–mRNAs, is largely unchanged in the vast majority of the strains examined (Figure 4). Likewise, an analysis of the cellular levels of the Faa1 mRNA, an intronless gene whose transcript abundance is of a similar magnitude as the Rpl31b, Tef5, Tub3 and Ubc13 pre–mRNAs, also shows a nearly constant level in all of the examined strains, further suggesting that there is no inherent bias in detecting low level transcripts. Finally, the Yra1 pre–mRNA, which is inefficiently spliced and has a higher endogenous level than most pre–mRNAs, also shows very little change in the examined strains. Taken together, these results strongly support the conclusion that the levels of the Rpl31b, Tef5, Tub3 and Ubc13 pre–mRNAs are increased in these strains.

### Increases in precursor levels correlate with a decrease in splicing efficiency

Because our approach, as described so far, directly measures the cellular levels of precursor RNA but does not directly determine the efficiency of splicing *per se*, those mutations which cause an increase in the precursor levels could be doing so simply by increasing the transcriptional frequency of these genes rather than by directly impacting their splicing. To distinguish this possibility from a true splicing defect, we chose to directly calculate the splicing efficiency of the Tef5 transcript by measuring the total cellular level of Tef5 mRNA by QPCR in each strain and using this value to calculate the ratio of unspliced∶spliced RNA in the cell, a classical measure of splicing efficiency. Consistent with a splicing rather than transcriptional cause for precursor accumulation, the measured levels of total Tef5 transcript showed little variation across nearly the entire set of strains (Figure S2). Indeed, nearly every strain that showed an increase in Tef5 pre–mRNA levels also showed a decrease in the splicing efficiency of the Tef5 transcript (Figure 4), suggesting that those mutations affect the splicing of this transcript rather than its transcription. These results strongly suggest that the increased pre–mRNA levels observed in these strains largely reflect changes in pre–mRNA splicing.

To assess the functional significance of the strains displaying increased pre–mRNA levels, we sought to rule out the possibility that mutations which cause a change in overall cellular fitness might indirectly lead to a decrease in overall splicing efficiency. To test this, we compared our precursor accumulation levels with recently described strain fitness data calculated for each of the 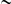
5000 non-essential genes [36]. This comparison yielded no correlation between precursor accumulation and cellular fitness (Figure S2), suggesting that cellular growth rate alone is insufficient to explain the observed increase in pre–mRNA levels.

### Assessing the statistical significance of the precursor accumulation

While the precursor accumulation seen for each of the canonical transcripts in the known splicing mutants lends strong empirical support for the overall robustness of our approach, additional analysis was needed to assess the statistical significance of the data we generated. Towards this end, we employed a statistical approach originally developed for analysis of microarray data called Significance Analysis of Microarrays [37], or SAM (see also Materials and Methods). We chose this software because, conceptually, the data generated by our QPCR approach are orthogonal to those from a microarray experiment: whereas a microarray experiment examines the behavior of thousands of mRNAs in a single strain, here we examine the behavior of a single RNA in thousands of different strains. Because similar concerns regarding multiple hypothesis testing apply to both types of data [38], we used this software as a tool for assessing the quality of our data. The results of our SAM analysis were consistent with the qualitative results seen in Figure 4, in so much as the number of strains causing a statistically significant increase in the levels of each precursor species varied depending upon the precursor mRNA in question. A total of 224 strains caused a statistically significant increase in the Rpl31b pre–mRNA levels, 209 strains caused a significant increase in Ubc13 pre–mRNA levels, 146 strains caused a significant increase in U3 precursor levels, 83 strains caused a significant increase in Tef5 pre–mRNA levels, and 78 strains caused a significant increase in Tub3 pre–mRNA levels. The complete list of SAM-identified strains for each RNA species is provided in Table S2. Importantly, many of the SAM-identified strains are found to cause a significant enrichment of the precursor levels of all five of these RNAs, including the majority of strains with mutations in canonical splicing factors.

Interestingly, for some of the species examined, a small number of strains were identified which showed *decreased* levels of precursor RNA. In certain instances these reflected expected outcomes: a large decrease in the Ubc13 precursor was identified in the *ubc13Δ* strain, for example. However, in other cases these may indicate important biological phenomena. For example, both the *xrn1Δ* and the *tfg2Δ* strains cause a significant decrease in the U3 precursor levels. We have previously shown that deletion of the Xrn1 nuclease paradoxically leads to decreases in many precursor RNAs [39], although the mechanism by which this occurs remains unknown. Likewise, it is unclear whether the decreased precursor resulting from deletion of the TFIIF component Tfg2 reflects an overall decrease in transcription of this gene, or whether this in fact reflects increased splicing efficiency perhaps resulting from a decreased transcription elongation rate [10].

### Top screen candidates predict specific links between splicing and several pre–mRNA processing pathways

To better characterize the factors that impact pre–mRNA splicing, we examined our lists of SAM-identified candidates for factors that are not canonical components of the spliceosome. As an initial approach, we asked whether any functional categories of proteins were statistically overrepresented within this set of strains. For this analysis, we ordered the strains according to the largest precursor accumulation that they affected for any of the RNA species. We then used the *GO::Term Finder* program [40] to identify overrepresented classes of genes. As expected, when considering the 50 strains that caused the largest precursor accumulations, a strong enrichment for splicing factors was seen with 30 out of 50 strains containing mutations in genes belonging to the GO PROCESS: *RNA Splicing* category (p = 1.3E-40 with Bonferroni correction). Interestingly, when the top 100 strains are considered, significant enrichment can also be seen for strains with mutations in factors belonging to the GO PROCESS: *Chromatin Remodeling* category, with eight different mutants causing precursor accumulation (*arp5Δ*, *arp8Δ*, *bdf1Δ*, *npl6Δ*, *rsc2Δ*, *rsc9-ts*, *vps72Δ*, and *yaf9Δ*; p = 1.5E-03). Expanding our analysis to the top 200 candidates increases the enrichment of this category to include twelve factors (adding *arp6Δ*, *swc5Δ*, *swr1Δ*, and *taf14Δ*; p = 1.8E-04). Interestingly, within the top 200 candidates, significant enrichment is also seen for the GO PROCESS: *RNA Catabolic Process* category, with 13 different factors being present (*ccr4Δ*, *dis3-ts*, *dbr1Δ*, *kem1Δ*, *lsm2-ts*, *lsm6Δ*, *lsm7Δ*, *prp18Δ*, *rrp6Δ*, *rtt101Δ*, *ski3Δ*, *ssn2Δ*, and *upf3Δ*; p = 8.5E-03). Whereas some of these factors, such as *lsm2-ts*, *lsm6Δ*, *lsm7Δ*, and *prp18Δ* are known to directly function in pre–mRNA splicing, the identification of many of these factors presumably reflects their defects in degradation pathways for unspliced pre–mRNAs.

**Figure 5:**
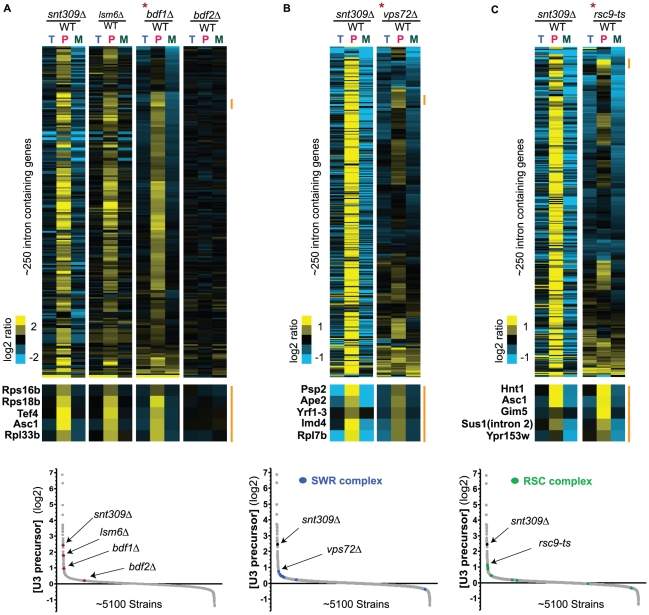
Mutations in chromatin remodeling factors can impact splicing in a global or transcript-specific manner. Splicing sensitive microarrays of candidate factors (A) *bdf1Δ* and *bdf2Δ*, (B) *vps72Δ*, and (C) *rsc9-ts*, as compared to the phenotypes of known splicing mutants *snt309Δ* and *lsm6Δ*. For each panel, the asterisk indicates the strain by which the data have been organized using hierarchical clustering, with the other data sets in those panels sharing an identical gene ordering. The orange bar highlights the location of specific subsets of transcripts showing splicing defects. The bottom insets show the location of each of the candidates within the U3 precursor dataset. Also, the locations of each of the components of the SWR complex are shown in blue, and each of the components of the RSC complex are shown in green.

One of the top factors we identified that bridges chromatin remodeling with transcription initiation is the bromodomain factor Bdf1. Bdf1 is a member of the SWR1 complex and, along with its homolog Bdf2, has been shown to interact with the TFIID component of RNA polymerase II [41]. Moreover, *BDF1* and *BDF2* have been demonstrated to be genetically redundant with one another. Whereas our SAM analysis indicated that the *bdf1Δ* caused a statistically significant accumulation of most of the canonical precursor species in our experiments, the *bdf2Δ* strain showed little or no detectable increase in the levels of any of the precursors tested (Figure 5A), and was not considered by SAM analysis to be significant for increases in any of the precursor RNAs. To better characterize the global splicing profile of these two mutants, we again turned to our splicing-sensitive microarrays. Remarkably, a dramatic splicing defect can be seen in the *bdf1Δ* strain for most intron-containing genes, as evidenced by an increase in the precursor transcript levels with a concomitant decrease in the mature and total transcript levels (Figure 5A). By comparison, the *bdf2Δ* mutation has almost no effect on cellular splicing, strongly corroborating the specific identification of Bdf1 in our screen. To better assess the mechanism by which Bdf1 impacts pre–mRNA splicing, we monitored U1 snRNP recruitment in the background of wild-type, *bdf1Δ*, and *bdf2Δ* strains using chromatin immunoprecipitation coupled to QPCR (ChIP-QPCR). As seen in Figure S3, these experiments show that the deletion of Bdf1 but not of Bdf2 decreases the occupancy of U1snRNP at several intron-containing genes, suggesting impairment of co-transcriptional spliceosomal recruitment in the *bdf1Δ* strain. A more comprehensive ChIP-Seq experiment will be required to fully characterize the global landscape of genes impacted by the deletion of Bdf1 and further characterize the roles of Bdf1 and Bdf2 in transcription and splicing.

We also chose to further examine several factors that our screen identified that are more classically connected with chromatin remodeling. The lower panels of Figure 5B and 5C show the locations within our U3 precursor dataset of all of the strains containing mutations in components of the SWR1 complex, and the RSC complex, respectively. Notably, mutations in many but not all of the components of these complexes cause a splicing defect of the U3 transcript. Moreover, each of the five precursor species that we examined shows a slightly different susceptibility to the different components of these complexes. We chose to examine the global splicing defects of strains containing mutations in two of these components: Vps72, a member of the SWR1 complex; and Rsc9, a member of the RSC complex. Splicing-sensitive microarrays of the *vps72Δ* and *rsc9-ts* strains, respectively, reveal a splicing defect in each strain (Figure 5B and 5C). However, unlike the *bdf1Δ* strain, the *vps72Δ* and *rsc9-ts* strains cause a splicing defect in only distinct subsets of intron-containing genes. Interestingly, the affected subsets of transcripts are neither completely overlapping nor completely independent of one another; rather the microarray data are consistent with our QPCR data in suggesting that mutations in specific chromatin-modifying components can result in aberrant splicing of specific pre–mRNA transcripts.

**Figure 6:**
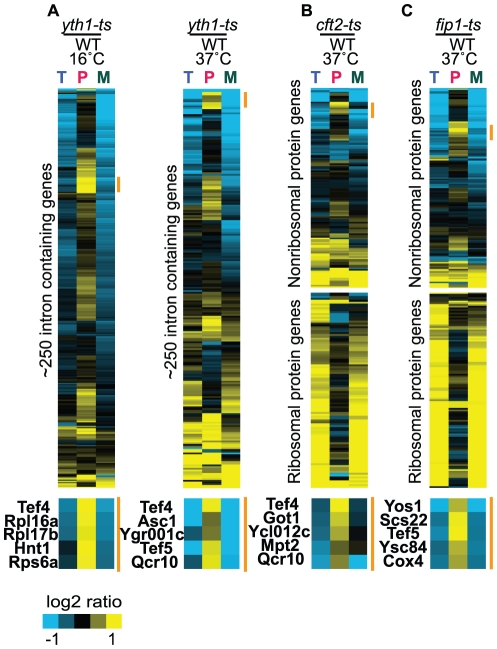
Mutations in 3′ end processing mutants result in transcript-specific splicing defects. Splicing sensitive microarrays for the cleavage and polyadenylation factor mutants *yth1-ts* (A), *cft2-ts* (B), and *fip1-ts* (C). For each panel, the data have been independently organized using hierarchical clustering. The orange bar highlights the location of specific subsets of transcripts showing splicing defects.

While an ontology-based approach can successfully identify entire pathways that display enrichment, we were also interested in considering those factors which showed strong pre–mRNA accumulation but whose functional categories were not statistically over-represented at the top of our dataset. Remarkably, while the GO PROCESS: *RNA 3′ end Processing* wasn't significantly overrepresented as a category within our dataset (9 out of top 200, p = 0.09), several strains with mutations in factors belonging to this category resulted in a strong, statistically-significant accumulation of multiple precursor species. Included among these were: *yth1-ts*, a zinc-finger containing protein that is the homolog of human CPSF-30; *cft2-ts*, the homolog of human CPSF-100; and *fip1-ts*, a component of the polyadenylation factor PF I. To further examine the global splicing defects of each of these mutants, microarrays were performed comparing mutant and wild type behavior after shifting them to both elevated and reduced temperatures. Of the three mutants, the profile seen in the *yth1-ts* mutation most closely resembles a canonical splicing defect, with more than half of the genes showing an increase of precursor and loss of mature RNA (Figure 6A). Interestingly, the splicing defect is strongest at reduced temperatures even though this strain has only a subtle low-temperature growth defect (not shown). By comparison, neither the *cft2-ts* nor the *fip1-ts* strains showed a strong splicing defect at low temperature (not shown), but each mutant was characterized by an unusual phenotype at elevated temperatures. As seen in Figure 6B and 6C, two distinct types of behavior are seen in the *cft2-ts* and *fip1-ts* mutants, respectively, that are largely defined by whether or not the affected transcript encodes a ribosomal protein gene (RPG). For a subset of the non-RPG transcripts a canonical splicing defect is apparent, consistent with our QPCR results. Interestingly, the subset of affected non-RPG transcripts is different between the two mutant strains. By comparison, nearly all of the RPG transcripts show a dramatic increase in both the mature and total mRNA levels, with little or no detectable change in precursor levels. The strong increases caused by these mutants suggest that the RPG transcripts may be subject to regulatory control at their 3′ ends. Interestingly, while it has long been known that RPG introns are, in general, longer than non-RPG introns [42], whereas the second exons of RPGs tend to be shorter than non-RPGs [5], we nevertheless find no strong correlation between either intron or second exon length and the strength of the splicing defect seen for these 3′ end mutants (data not shown). The mechanisms by which these 3′ end factors impact pre–mRNA splicing are currently under investigation.

### Top screen candidates do not cause changes in the cellular mRNA levels of most spliceosomal components

In considering the mechanisms by which candidate factors may be functioning, we sought to determine whether any of the candidates we examined might be indirectly affecting pre–mRNA splicing by changing the cellular levels of known spliceosomal components. Although our splicing-sensitive microarrays were designed primarily to interrogate the splicing status of the ∼300 intron-containing genes in *S. cerevisiae*, they also contain probes against all ∼6000 protein-coding genes and ∼200 RNA genes, including the spliceosomal snRNAs. Figure S4 shows the relative RNA levels for each of the canonical spliceosomal components, including the snRNAs, in the background of each of the different strains we examined, as determined from our microarray analyses. While these results positively recapitulate the expected changes (for example, the decreases in Snt309 and Lsm6 mRNA levels in the *snt309Δ* and *lsm6Δ* strains, respectively), with only a few exceptions, most spliceosomal components appear unchanged in most of the mutants we examined. Importantly, the transcript encoding the Mud1 protein showed dramatic mis-regulation in both the *yth1-ts* and *cft2-ts* strains, increasing by more than 10-fold in each background. To test whether Mud1 overexpression might be causing the splicing defects observed in these strains, a strain was constructed where the wild type Mud1 transcript was encoded on a high-copy plasmid. As seen in Figure S5, in spite of the over 30-fold increase in Mud1 levels in this strain, there is no detectable change in pre–mRNA splicing. Therefore, while the mis-regulation of Mud1 levels in these 3′ end mutants suggests that, similar to its human homolog, Mud1 levels in yeast may be subject to negative regulation via its 3′ end processing [43], it nevertheless appears that the splicing defect observed in these strains is not a consequence of Mud1 overexpression.

Interestingly, several of the strains, including *bdf1Δ*, *yth1-ts*, *cft2-ts*, and *fip1-ts*, showed an ∼2-fold *increase* in the levels of both the U1 and U2 snRNAs. Although spliceosomes function as an equimolar complex of all five snRNAs, the total cellular levels of the snRNAs vary: in yeast, the U2 snRNA is the most abundant [44], while in mammals the U1 snRNA is most abundant [45]. While recent work demonstrates the cellular defects associated with decreased levels of snRNA [46], it is less clear whether increases in their levels will impart a defect on global splicing. Nevertheless, because each of these strains shows a similar increase in these snRNA levels but distinct splicing defects, it seems unlikely that the changes in snRNA levels alone can explain the observed splicing phenotypes. However, it is not inconceivable that small changes in levels for one or more of these transcripts could lead to the observed splicing defects. As such, additional work will be necessary to determine the functional consequences of these mutations.

## Discussion

### A high-throughput, reverse genetic approach to measure the cellular levels of specific RNA species

Here we present the results of a global survey designed to identify the full subset of cellular factors in the budding yeast, *Saccharomyces cerevisiae*, that impact the efficiency of pre–mRNA splicing. As a complement to other recently described genetic and physical genome-wide approaches, in this work we have developed an approach that allows for a direct readout of the accumulation of specific RNA species in the background of thousands of different mutant strains. An important strength of a genome-wide screen such as this is its unbiased approach. By directly measuring the splicing efficiency of endogenous transcripts, this method avoids bias generated using reporter constructs. Moreover, the ability to examine numerous different transcripts allowed us to distinguish the natural variation in the spliceosomal factors that are required for efficient splicing of different intron-containing transcripts. Indeed, by systematically examining the precursor levels in the background of each strain, mutations can be identified which result in a change in splicing efficiency regardless of their previously described functions. In the work described here, mutations in scores of genes with no previously known role in splicing were identified, some of which impacted the splicing of all five canonical transcripts examined and some of which impacted only a subset of them. While some of these factors have been further characterized and discussed here, many have not (see Table S2). To be sure, as is the case with all genetic screens, it is impossible on the basis of these screen data alone to ascribe a direct role for any of these candidate factors in the splicing pathway. Rather, the identification of these different factors can be seen as generating a rich dataset from which hypotheses can be generated and tested for their mechanistic underpinnings.

### The 5′ end: Connecting splicing with chromatin remodeling and transcription initiation

Beyond known splicing factors, the most highly over-represented set of factors identified in this work function in chromatin remodeling. One particularly interesting mutation that was identified was the *bdf1Δ* mutant. In budding yeast, Bdf1 has been demonstrated to play a role precisely at the interface of transcription initiation and chromatin remodeling. Based in part on its physical interaction with the Taf7 subunit of TFIID, yeast Bdf1 has been proposed to function as the missing C-terminal portion of the higher eukaryotic TAF_II_250 [41], the largest subunit of the TFIID complex. More recently, it has become clear that Bdf1 interacts with Swr1 and functions in recruiting the entire SWR1 chromatin remodeling complex to nucleosomes. A recent genome-wide study demonstrates that Bdf1 is enriched on the +1 and +2 nucleosomes of actively transcribed genes [47], and that it coincides with the localization of Vps72, another component of the SWR1 complex, and another component which was identified in our screen (Figure 5).

Remarkably, we demonstrate here that the splicing of nearly every intron-containing gene is negatively affected in a *bdf1Δ* strain, and that the quantitative defect seen in this mutant rivals that seen for canonical splicing mutants. Given its role in global gene expression, one possible explanation for our results in the *bdf1Δ* strain is that the transcription of some key splicing factor is repressed by this mutation, causing a decrease in splicing efficiency. Indeed, early work on Bdf1 from the Séraphin lab suggested a role in global transcription, including transcription of the spliceosomal snRNA genes [48]. However, our microarray analyses show essentially normal RNA levels of all known splicing components in the *bdf1Δ* strain (see Figure S4). Moreover, our microarray data assessing the snRNA levels themselves are entirely consistent with Séraphin's original observations and demonstrate that none of the five wild type snRNAs are decreased in cellular level during growth at 30°C in the *bdf1Δ* mutant; rather, there are subtle increases in the U1 and U2 snRNA levels. Importantly, our ChIP-QPCR experiments in the *bdf1Δ* strain demonstrate a decreased occupancy of the U1 snRNP on all four intron-containing genes we tested, suggesting the intriguing possibility that Bdf1 plays a direct role in connecting pre–mRNA splicing with chromatin remodeling and transcription initiation.

In considering such a role for Bdf1, it is important to note that the yeast *BDF1* gene has a close sequence homolog in the *BDF2* gene. These two genes are genetically redundant, in so much as both single gene deletions are viable but the double mutant *bdf1Δ/bdf2Δ* is lethal. Moreover, it has been shown that these two genes evolved from a single ancestral gene following a whole-genome duplication event [49]. Yet surprisingly, unlike the *bdf1Δ* strain, the *bdf2Δ* strain showed no signs of a splicing defect either in our screen or when examined by splicing sensitive microarrays. Moreover, unlike the *bdf1Δ* strain, there was no apparent decrease in U1 snRNP ChIP-QPCR signal in the *bdf2Δ* strain. In considering a mechanism whereby Bdf1 connects transcription initiation and chromatin remodeling with pre–mRNA splicing, it is worth noting that, unlike human genes, the majority of yeast genes do not contain an intron. As such, co-transcriptional recruitment of the spliceosome is unnecessary for most yeast genes. We are intrigued by the possibility that, in the time since the duplication event, Bdf2 has evolved to a point where it retains the capacity to recruit RNA polymerase but has lost the ability to efficiently connect splicing with transcription. Such a scenario would explain the differences observed between the *bdf1Δ* and *bdf2Δ* microarrays and U1 snRNP ChIP-QPCR data. It would also likely explain the previously published results that Bdf1 shows higher sequence conservation with the C-terminal domain of human TAF_II_250 than does Bdf2 [41]. Given such a model for the divergence of Bdf1 and Bdf2 functions, the differences in protein sequence between these two proteins may prove informative for deciphering the mechanism of Bdf1 activity.

### The 3′ end: Connecting splicing with cleavage and polyadenylation

**Figure 7:**
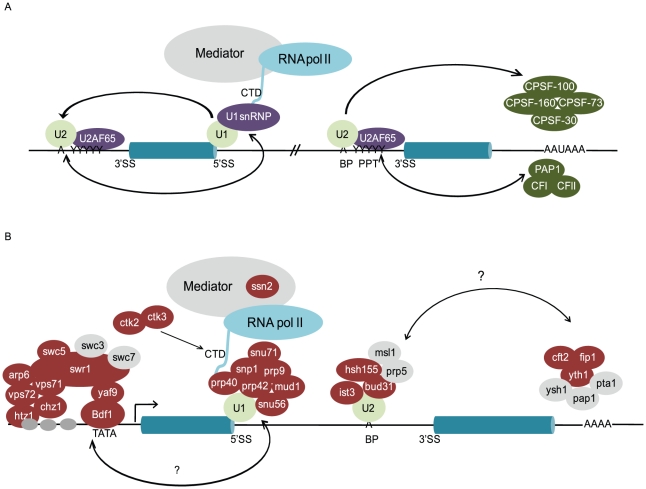
The location of mutants that disrupt splicing suggests a similarity to exon-definition in mammalian splicing. A. Spliceosome assembly in mammalian systems is thought to occur via exon-definition, where interactions between U1 and U2 snRNP components across an internal exon, or CPSF components and U2 snRNP across a terminal exon, are necessary for efficient processing of the upstream intron. B. Functional location of a subset of the mutants examined in this work, presented on a model transcript. Factors shown in red caused a statistically significant increase in the precursor levels of at least one transcript in our screen, whereas those in grey did not affect the splicing of any of the transcripts.

In addition to the over-representation of factors marking the 5′ end of genes, our screen identified a number of factors involved in the 3′ end processing of mRNAs. Splicing-sensitive microarrays confirm a broad splicing defect in a mutant of Yth1, the homolog of human CPSF30, and transcript-specific splicing defects in mutants of Cft2 and Fip 1, the homolog of human CPSF100 and a component of the polyadenylation factor complex PF I, respectively. In higher eukaryotes, components of the 3′ end processing machinery have been shown to physically associate with components of the U2 snRNP [50] and U2AF65 [51]; moreover, *in vitro* studies demonstrate a functional link between the pre–mRNA splicing and 3′ end processing pathways [52]. The interactions between these two pathways in mammalian systems have led to the proposal that the 3′ end machinery plays an important role in terminal exon definition. Whereas the exon-definition model for mammalian spliceosome assembly posits that internal exons are defined by interactions between U1 and U2 snRNP components across an exon [53], definition of terminal exons is achieved by interactions between the 3′ end processing machinery and the U2 snRNP (Figure 7A), imposing a functional connection between the pathways. Yet because of the relatively short length of *S. cerevisiae* introns, and the limited number of genes that are interrupted by multiple introns, splicing in yeast has long been considered to proceed through a model of intron-definition. Nevertheless, the Keller lab recently demonstrated that some conditional alleles of *YSH1*/*BRR5* lead to a decrease in splicing efficiency [54]. Our demonstration here of pre–mRNA splicing defects in the background of additional mutants in 3′ end processing mutants suggests the intriguing possibility that some of the basic interactions that facilitate exon-definition in higher systems may also be present in budding yeast. Indeed, further characterizing the mechanism by which these 3′ end processing factors are affecting splicing in yeast may provide important insights into the mechanisms by which exon-definition is accomplished in higher eukaryotes.

### A global perspective

An important strength of an approach such as this is the genome-wide perspective that it provides. Figure 7B shows a model of an idealized transcript along with the functional location of a subset of the factors that have been examined in our screen. It is striking to note that many of the factors identified here function both during transcription initiation (Bdf1 and others) and termination (Yth1, Cft2, Fip1, and others), thereby defining the beginning and ends of the first and last exons, respectively. In this work, we have identified not only those factors whose disruption leads to a functional defect in splicing efficiency, but in many cases the specific transcripts whose splicing is affected.

More broadly, the work presented here demonstrates the feasibility of quantitating cellular RNA levels in the background of large mutant strain collections. While our current approach examined splicing efficiency in the context of optimized growth conditions, a similar approach could be applied to identify factors necessary for efficient splicing under varying cellular or developmental growth states. Likewise, although our work focused on the levels of several pre–mRNA species, this methodology should be directly applicable to assessing the levels of nearly any cellular RNA of interest.

## Materials and Methods

### High-throughput strain handling

All experiments were performed using haploid strains. To assess the function of non-essential genes, the mat a version of the haploid deletion library from Open Biosystems [55] was used (referred to herein as non-essential strains). Likewise, to assess the function of essential genes, a collection of strains provided by the Hieter lab [25] was used (referred to herein as essential strains). In addition, a collection of strains containing previously characterized mutations in core spliceosomal components was used (from here on considered a part of the essential strains set). A complete list of the strains used in this work is included in Table S3. Unless otherwise indicated, all strains were grown in rich medium supplemented with 2% glucose (YPD) [56]. When appropriate, strains were recovered from frozen glycerol stocks on solid medium supplemented with 200 µg/ml G418 grown at either 30°C (non-essential strains) or 25°C (essential strains). A manual pinning tool (V&P Scientific, cat.#: VP384FP6) was used to transfer cells from solid medium into 384-well microtiter plates (Greiner BioOne, cat.#: 781271) for growth in liquid media. Liquid cultures were grown in an Infors HT Multitron plate shaker at 900 rpm with 80% constant humidity. Breathable adhesive tape (VWR, cat.#: 60941-086) was used to seal the plates and reduce evaporation.

Because the growth rates of the strains being used vary significantly [36], an approach was developed to enable the systematic collection of a similar number of rapidly dividing cells for each strain. An initial liquid culture was grown in 384-well plates for two days, allowing nearly all strains to reach saturation. Because all of the strains being used are derived from a common parental strain, the cell density for each of these strains is nearly identical at saturation, allowing us to effectively ‘normalize’ the cell numbers. Using a liquid handling robot (Biomek NX), 2 µl of saturated culture were used to inoculate a fresh 150 µl of YPD. This culture was allowed to grow for four hours, an amount of time which is sufficient to allow all strains to exit lag-phase and begin exponential growth, but not so long as to result in a large variation in cell densities among the strains (Figure S6). For the non-essential strains, all growth was conducted at 30°C. For the essential strains, the initial growth was done at 25°C (a permissive temperature for all strains), but the saturated cells were back-diluted into plates containing media pre-warmed to 30°C (a non-permissive temperature for many, but not all, of the strains) and allowed to continue growing at 30°C for four hours. For both the non-essential and the essential strains, two independent biological replicates were initiated from each saturated plate. After four hours of outgrowth, cells were harvested by centrifugation at 4000*×g* for five minutes. The cell pellets were flash frozen in liquid N_2_ and stored at −80°C until further processing.

### High-throughput RNA isolation

Isolation of total cellular RNA was performed using custom protocols written for a Biomek NX liquid handling system. To each frozen cell pellet collected as described above, 50 µl of Acid Phenol: Chloroform (5∶1, pH<5.5) and 25 µl of AES buffer (50 mM sodium acetate (pH 5.3), 10 mM EDTA, 1%SDS) were added. The plates were sealed with plastic CapMats (Greiner BioOne, cat.#: 384070) and vortexed for five minutes at top speed on a plate vortex. The plates were incubated for 30 minutes in a water bath at 65°C with intermittent vortexing. After incubation, the plates were spun for one minute at 1000×*g*. An additional 35 µl of AES buffer was added to each well, and after mixing the organic and aqueous phases were separated by centrifugation for five minutes at 3000×*g*. Using a slow aspiration speed, 40 µl of the upper phase containing the RNA were robotically transferred to a new 384-well microtiter plate. The transferred aqueous phase was mixed with 3 volumes of RNA Binding Buffer (2 M Guanidine-HCl, 75% isopropanol) and passed through a 384-well glass fiber column (Whatman, cat.#: 7700-1101) by centrifugation for two minutes at 2000×*g*. The column was washed twice with two volumes of Wash Buffer (80% ethanol, 10 mM Tris-HCl (pH 8.0)), followed by a final dry spin for two minutes at 2000×*g*. To remove any contaminating genomic DNA, 5 µl of DNase Mix (1× DNase Buffer, 0.25 units of DNase I (Promega)) was added to each well and incubated at room temperature for 15 minutes. After the incubation, 80 µl of RNA Binding Buffer was added to each well of the 384-well glass fiber plate and spun as before. After washing and drying as above, 15 µl of sterile water was added to each well of the glass-fiber plate to elute the RNA into a clean 384-well microtiter plate (Greiner BioOne, cat. #: 781280). In general, this procedure yielded about 1 µg of total cellular RNA from each cell pellet. The quality of the RNA produced by this protocol is equal to our conventionally purified samples, and the effectiveness of the DNase treatment is demonstrated in Figure S7.

### Synthesis of cDNA and quantitative PCR

Total cellular RNA was converted into cDNA in 384-well microtiter plates. Of the 15 µl of RNA purified as described above, 10 µl were used in a cDNA synthesis reaction that had a total volume of 20 µl and which contained 50 mM Tris-HCl (pH 8.3), 75 mM KCl, 3 mM MgCl_2_, 10 mM DTT, 0.5 mM each dNTP, 5 µg dN_9_ primer, and 60 ng M-MLV RT. Reactions were incubated overnight at 42°C.

The cDNA reactions were diluted 30-fold with water, giving a final concentration of ∼1 ng/µl based on the initial RNA concentration, and used without any further purification as templates in high-throughput quantitative PCR (QPCR) reactions. The QPCR reactions were performed in a reaction volume of 10 µl, containing 5 µl of template (∼5 ng of template), 10 mM Tris-HCl (pH 8.5), 50 mM KCl, 1.5 mM MgCl_2_, 0.2 mM each dNTP, 0.25× SYBR Green, 5% DMSO, 0.7 ng *Taq* DNA polymerase, and 250 nM forward and reverse primers. The sequences of the primers used for each targeted RNA are shown in Table S4. Standard curves were generated consisting of 4-fold serial dilutions of genomic DNA and covering a range of 1.6×10^5^ molecules. Each primer pair was well-behaved, showing an amplification efficiency of between 86% and 97% (Figure S8). Two technical replicates were measured for each biologically independent sample, generating four independent measurements for each of the ∼5500 mutant strains.

### Processing QPCR data

On the basis of standard curves generated using QPCR, relative nanogram quantities were calculated for every RNA transcript within each of the ∼5500 strains tested. To assess reproducibility, coefficients of variation (CV) were determined for each primer pair and each strain. The vast majority of these were highly reproducible, both overall and on a per plate basis. As an initial quality filter, we chose to exclude any samples for which the CV was greater than 0.25.

Because no simple mechanism exists to normalize for variability in each of our experimental steps, we instead chose to measure the levels of six different RNAs in each of the samples and use these to determine a composite normalization value to account for the overall yield in our procedure. The six RNAs were: U1 snRNA, Scr1 (SRP) RNA, Tef5 mRNA, Tub1 mRNA, Srb2 mRNA and Faa1 mRNA. These RNAs were chosen because their biological functions are diverse and their cellular levels vary over a broad range (∼300-fold, Table S1). For both independent biological replicates of every strain, a composite normalization constant, 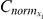, was calculated according the following formula:
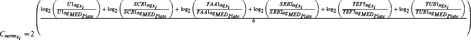
For each primer pair, 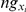 represents the relative nanogram quantity calculated for an individual sample. Similarly, 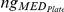 represents the median value determined for a given primer pair on an individual QPCR plate run. Because of the subtle variations that are apparent from one plate run to the next, we found that this per plate normalization using 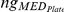 gave us the most robust data. By determining the ratio of 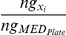 for every primer pair, a relative abundance of total RNA can be calculated for every sample. As seen in Figure S9, a histogram of 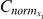 values follows a normal distribution in log_2_ space with a variance of 1.5 units. A second filter at the level of 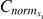 values was introduced which allowed for the filtering of samples with very low amounts of cDNA.

For strains that passed this filter, the normalized levels of a given RNA were determined according to the following formula:
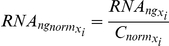
The relative amount of RNA in a given strain was then determined according to the following formula:
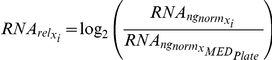
For each primer pair, 
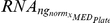
 represents the median value of the normalized RNA levels determined within a given QPCR plate. For each biological replicate of every strain, both the 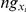 and the 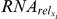 values are available through Gene Expression Omnibus (GEO) using accession number GSE34330.

### Significance analysis of QPCR data

To determine the subset of strains that cause a statistically significant increase or decrease in precursor levels, we employed the *Significance Analysis of Microarrays*, or SAM, program [37]. While this software was originally designed for the analysis of microarray data, a significance analysis of our QPCR data is subject to similar concerns regarding multiple hypothesis testing. For each RNA, SAM analysis was performed on the four 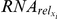 values, comprised of both technical and biological replicates that were generated for each of the ∼5500 strains. For each transcript, a one class SAM analysis was performed where the Δ value was adjusted to minimize the false discovery rate (FDR), yielding the following values: for the U3 precursor using Δ = 0.983, FDR = 0.045; for the Tub3 pre–mRNA using Δ = 0.91, FDR = 0; for the Rpl31b pre–mRNA using Δ = 1.061, FDR = 0.003; for the Ubc13 pre–mRNA using Δ = 0.978, FDR = 0.002; and for the Tef5 pre–mRNA using Δ = 0.99, FDR = 0.

### Splicing-sensitive microarrays

The candidate non-essential deletion strains were grown to saturation in YPD at 30°C, then back diluted in 25 ml cultures in flasks at a starting A_600_∼0.2 and allowed to grow at 30°C until they reached an optical density of between A_600_ = 0.5 and A_600_ = 0.7. The candidate essential strains were initially grown at 25°C in YPD, then shifted to the indicated temperatures for 15 minutes after they reached an optical density of between A_600_ = 0.5 and A_600_ = 0.7. In parallel with the collection of the mutant strains, wild type isogenic controls were grown and collected under the same conditions as the mutant strains examined. Total cellular RNA samples were isolated, converted into cDNA, and fluorescently labeled as previously described [31]. All microarrays were performed as two-color arrays comparing mutant and wild type strains, each grown under identical conditions. Both raw and processed microarray data are available through GEO using accession number GSE34330.

### Chromatin immunoprecipitation

The U1C-Tap *bdf1Δ* and U1C-Tap *bdf2Δ* strains were generated by deleting the appropriate genes in the background of the U1C-Tap strain from Open Biosystems [57] using standard techniques. The strains were grown at 30°C in rich medium supplemented with 2% glucose (YPD) until they reached an optical density of A_600_∼0.7. The chromatin was cross-linked with 1% formaldehyde for 2 minutes at 30°C. Glycine was added at a final concentration of 125 mM and the cultures were left shaking for another 5 minutes. Cell pellets from 50 ml of culture were collected by centrifugation at 1620×g for 3 minutes, then washed with 25 ml ice-cold 1× PBS and the pellet stored at −80°C. The pellets were resuspended in 1 ml Lysis buffer (50 mM Hepes pH 7.5, 140 mM NaCl, 1 mM EDTA, 1% TritonX-100, 0.1% Na-deoxycholate supplemented with protease inhibitors) and lysed in the presence of 500 µl 0.5 mm glass beads in a beat beater. The lysate was collected by centrifugation at 1000×g for 1 minute, and then pre-cleared by spinning for 15 minutes at 14000 rpm in a tabletop centrifuge at 4°C. The pellet was re-suspended in another 1 ml of Lysis buffer and the chromatin was sheared to an average size of 300 bp (range 100–500 bp) by means of a Bioruptor sonicator. The sample was clarified by 2 cycles of centrifugation at 14000 rpm for 15 minutes in a tabletop centrifuge at 4°C and the resultant chromatin solution frozen and stored at −80 C. From the chromatin samples a 1% Input sample was retained, and then each sample was split equally between a Mock IP and an IP sample. The IP samples were incubated with 5 µl 0.5 mg/ml anti-Tap Antibody (Thermo Scientific, CAB1001). After 2 hours at 4°C on a rotator, 25 µl of protein A/G-agarose resin (#Sc-2003Santa Cruz) was added to all samples and they were further incubated for another 2 h at 4°C. The resin was washed twice with 1 ml Lysis buffer, twice with 1 ml Wash buffer (10 mM Tris-HCl, 25 mM LiCl, 0.5% NP-40, 0.5%Na-deoxycholate, 1 mM EDTA) supplemented with 360 mM NaCl, twice with 1 ml Wash buffer, and finally twice with 1 ml TE. The first wash was a brief one, followed by a 15 minute incubation of the samples on a rotator at 4°C for the second wash. In between washes, the resin was collected by short spins at 2000 rpm in a tabletop centrifuge. The resin was resuspended in 100 ul Elution buffer (50 mM Tris-HCl pH 8.0, 5 mM EDTA, 1% SDS) and the immunoprecipitated material was eluted from the beads by incubating at 65°C for 30 minutes with occasional tapping. To reverse crosslinks, the IPs and the 1% Input samples were incubated overnight in a 65°C water bath. The next day, the samples were treated with 12.5 µl 20 mg/ml Proteinase K solution and incubated at 42° for 2 h. The DNA was then purified by using a Cycle Pure Kit (Omega Bio-Tek, D6492-01) following the manufacturer's instructions and eluted in a final volume of 120 µl.

Quantitative real-time PCR was performed on a Roche Light Cycler 480 machine as described above, using the 1% Input sample to generate a standard curve for each of the primer pairs we used. For the primers used in the screen, the sequences are available in Table S4. The primers for the different regions of actin gene and the PMA1 gene are the same as previously published [5]. For each sample the Mock IP value calculated as percent input was subtracted from the IP value (in percent input). Then, a fold enrichment value was calculated, by dividing these values by the PMA1 value.

### Mud1 overexpression

An overexpressing plasmid containing a full-length copy of the Mud1 gene including ∼500 bp up- and down-stream of the ORF was transformed into BY4741 (Open Biosystems). This strain and a control strain containing the empty vector were grown in 25 ml minimal media until they reached an optical density of A_600_∼0.5–0.6. RNA isolation was performed as previously described [31], and cDNA synthesis and Q-PCR were performed as described above. The primer sequences are found in Table S4.

## Supporting Information

Figure S1 Splicing factors included within strain library. Within the “GO process: RNA splicing” box are listed all splicing factors as annotated by Gene Ontology [30]. These factors have been subdivided based on the presence or absence of a strain within our library, and further by the presence or absence of data from our final filtered dataset. The subset highlighted in red represents the strains which passed all the filters in the U3 snoRNA precursor screen. Strains for which there is “NO DATA” that passed our quality filters are listed separately. Factors for which “NO STRAIN” was present in our library are also noted. We “MANUALLY REMOVED” several strains that are included by Gene Ontology but that we felt are either are not part of the spliceosome or have a yet uncharacterized function. In addition, we have “MANUALLY ADDED” three factors which are known to impact splicing. Two more strains that partially “DISRUPT READING FRAMES” of specific splicing factors (indicated in the parentheses) were also added to our dataset.
(EPS)

Figure S2 Global changes in Tef5 RNA levels. A. Total RNA levels of the Tef5 transcript in 5198 strains which passed the quality filters. The total RNA levels are presented in log_2_ space as a composite behavior of both biological replicates, and are ordered from the highest (left) to the lowest (right) values. On the right side of the figure the data are presented as a heat map, with both biological datasets (A and B) shown. The data in the heat map are ordered from the highest to the lowest values, similar to the representation on the left. B. A comparison of the levels of Tef5 pre–mRNA versus the splicing efficiency of this transcript (calculated as a ratio of precursor Tef5 to total Tef5 levels across the entire dataset) shows a strong correlation. C. A comparison of the relative growth rate of the non-essential library strains [36] versus the Tef5 pre–mRNA levels reveals no correlation between cellular fitness and splicing efficiency.
(EPS)

Figure S3 U1 snRNP recruitment is diminished upon Bdf1 deletion. Chromatin immunoprecipitation (ChIP) was performed using a Tap tagged version of Yhc1 (U1C) in wild type, *bdf1Δ*, and *bdf2Δ* strains to assess the co-transcriptional occupancy of the U1 snRNP. A) Primers that had been previously used in a similar assay (Tardiff *et al.* Mol Cell 2006) allowed us to monitor by quantitative PCR the amount of U1 snRNP associated with different genomic regions of the ACT1 gene. The plotted values were calculated as the percent of input signal detected at given region within the actin gene divided by the percent of input signal observed for the intronless gene PMA1. The error bars represent the standard deviations of technical replicates. B) The ChIP samples described above were assayed with the same primers used in our screening which targeted intronic regions of the U3 snoRNA, Rpl31B and Ubc13 pre–mRNAs. As above, the values are presented as fold enrichment over the intronless gene PMA1 and the error bars are indicate standard deviation of technical replicates. For all four intron-containing genes, decreased levels of U1 snRNP are detected in the *bdf1Δ* strain relative to both the wild type and *bdf2Δ* strains.
(EPS)

Figure S4 The RNA levels of most spliceosomal factors are unchanged in most mutants. Total RNA levels for all splicing factors in the background of different gene deletions or point mutations for which microarrays were performed. The data are organized on the basis of the highest to the lowest average change in the *snt309Δ* and *lsm6Δ* strains.
(EPS)

Figure S5 Mud 1 overexpression does not cause increases in precursor levels. A high copy plasmid (2-micron) containing the Mud1 gene was transformed into an otherwise wild type strain in order to affect its overexpression. The expression levels of Mud1 and several precursor RNAs were monitored by quantitative real-time PCR and compared to a wild type strain containing an empty vector. The data were normalized to the expression of the intronless Faa1 transcript to account for loading differences in the samples. While a >30-fold increase is apparent for the Mud1 transcript, no increase is detected in the precursor levels of any of the RNAs surveyed, suggesting that their splicing is unaffected by Mud1 overexpression. The error bars represent the standard deviation of technical replicates.
(EPS)

Figure S6 Growth rates of mutant strains in liquid culture. Growth curves for a subset of 96 mutant deletion strains over a 600 minute time interval. Whereas the majority of the strains (A) grow at a rate which is similar to wild type, a small number of strains (B) grow at a slightly reduced rate, while one strain (C) grows very slowly. On the basis of these data, we chose to harvest cells after 4 hours of outgrowth, which ensures that the majority of the strains are harvested when A_600_∼0.5.
(EPS)

Figure S7 Assessing Total RNA and cDNA quality. A. A comparison of RNA quality between a “classic” phenol-extraction protocol and our robotic procedure. The diagram above the picture of the gel indicates the regions of a 384-well plate from which RNA was selected and run. Each lane contains about 300 ng of total cellular RNA and duplicates from each region of the plate are shown. B. Effectiveness of the DNase treatment as measured by quantitative RT-PCR, demonstrating the shift in fluorescence before and after DNase treatment. The green text indicates the samples that were treated with DNase, whereas the black text indicates the untreated samples. C. The measured levels of Tef5 pre–mRNA in the indicated samples demonstrates that the contaminating genomic DNA is strongly depleted by DNase treatment.
(EPS)

Figure S8 Standard curves for quantitative PCR (QPCR) for all primer pairs. Standard curves for all primer pairs used in this study. The Cp values (crossing points) are plotted against the logarithm of known starting sample amounts (in nanograms of genomic DNA). The equations that describe the curves as well as the R^2^ values are specified for each of the primer pairs.
(EPS)

Figure S9 Distribution of C_norm_ values. The distribution of the normalization constants (C_norm_) in log_2_ space for all samples in the biological replicate A dataset. The area highlighted in dark green comprises C_norm_ values which have passed the C_norm_ filter. All log_2_(C_norm_) values less than −3 (an 8-fold decrease from the median) were eliminated from the dataset. Above the histogram, a box and outlier plot underlines the same distribution of C_norm_ values.
(EPS)

Table S1 Relative abundances of RNA species as measured by QPCR. RNA species for which expression levels were measured and their corresponding levels. The values are normalized to the Tub3 pre–mRNA level, which was the lowest abundance transcript measured in our experiments.
(DOCX)

Table S2 List of SAM identified strains and their SAM score for the various pre–mRNAs measured. Strains which were found to be statistically significant for any of the five precursor RNAs we measured, arranged in the decreasing order of their maximal D score generated by SAM.
(DOCX)

Table S3 The complete list of the strains used in this study. The systematic names of all genes which were either deleted or contained a temperature sensitive mutation are noted, as well as their mutations and a reference to their sources. The sources are a = Giaver et al., 2002; b = Ben-Aroya et al., 2008; c = from the Guthrie lab collection.
(DOCX)

Table S4 Forward and Reverse primer sequences used for QPCR for each target RNA. The list of forward and reverse primers used in this study.
(DOCX)
