# Supplementary material for: A Quantitative, High-Throughput Reverse Genetic Screen Reveals Novel Connections between Pre–mRNA Splicing and 5′ and 3′ End Transcript Determinants
Source: PLoS Genet. 2012 Mar 29;8(3):e1002530. doi: 10.1371/journal.pgen.1002530 (PMC3315463; doi:10.1371/journal.pgen.1002530)
Supplement: Table S1 — Relative abundances of RNA species as measured by QPCR. RNA species for which expression levels were measured and their corresponding levels. The values are normalized to the Tub3 pre–mRNA level, which was the lowest abundance transcript measured in our experiments. (DOCX) [file pgen.1002530.s010.docx]

| **RNA** | **Relative abundance** |
| --- | --- |
| U1 total | 1800 |
| Scr1 total | 1200 |
| Tef5 total | 280 |
| Yra1 pre-mRNA | 110 |
| U3 precursor | 70 |
| Tub1 total | 35 |
| Faa1 total | 12 |
| Srb2 total | 5 |
| Rec107 pre-mRNA | 3 |
| Rpl31b pre-mRNA | 3 |
| Tef5 pre-mRNA | 3 |
| Ubc13 pre-mRNA | 2 |
| Tub3 pre-mRNA | 1 |
