# Supplementary material for: A Quantitative, High-Throughput Reverse Genetic Screen Reveals Novel Connections between Pre–mRNA Splicing and 5′ and 3′ End Transcript Determinants
Source: PLoS Genet. 2012 Mar 29;8(3):e1002530. doi: 10.1371/journal.pgen.1002530 (PMC3315463; doi:10.1371/journal.pgen.1002530)
Supplement: Table S2 — List of SAM identified strains and their SAM score for the various pre–mRNAs measured. Strains which were found to be statistically significant for any of the five precursor RNAs we measured, arranged in the decreasing order of their maximal D score generated by SAM. (DOCX) [file pgen.1002530.s011.docx]

| **NAME** | **Strain** | **U3** | **Tef5** | **Rpl31b** | **Tub3** | **Ubc13** | **Max score** |
| --- | --- | --- | --- | --- | --- | --- | --- |
| YKL149C | *dbr1Δ* | 42.22551967 |  | 11.82728566 |  |  | 42.22551967 |
| YLR147C | *smd3-ts* | 24.46192788 | 4.273348907 | 3.504766447 | 2.78578464 | 6.843757264 | 24.46192788 |
| YIR005W | *ist3Δ* | 24.4269788 | 5.007952572 | 5.46024035 | 2.847594351 | 8.792733716 | 24.4269788 |
| YGR278W | *cwc22-ts* | 22.3665944 | 3.890875467 | 4.282661509 | 2.207388784 | 6.240979063 | 22.3665944 |
| YHR165C | *prp8-1* | 21.08715014 | 4.140007321 | 4.171477706 | 4.493496417 | 6.474045238 | 21.08715014 |
| YLR423C | *atg17Δ* | 20.13981855 | 4.04583548 | 4.586851883 | 3.141942873 | 5.445348941 | 20.13981855 |
| YDL098C | *snu23-ts* | 18.08781305 | 5.245568367 | 3.812454951 | 3.725382375 | 5.459364848 | 18.08781305 |
| YPL151C | *prp46-ts* | 17.80260682 | 5.299065316 | 2.956984208 | 3.79662316 | 6.11991609 | 17.80260682 |
| YPR101W | *snt309Δ* | 16.31346804 |  | 4.84608278 | 5.357626415 | 7.550526599 | 16.31346804 |
| YNL147W | *lsm7Δ* | 15.79251572 | 4.227826468 | 4.154070784 | 2.087243045 | 3.451017304 | 15.79251572 |
| YLL036C | *prp19-1* | 14.40436194 | 4.158972165 | 4.370301114 | 4.809654062 | 4.710251798 | 14.40436194 |
| YKR086W | *prp16-2* | 14.38885557 | 3.231885445 |  | 2.592096586 | 2.973951754 | 14.38885557 |
| YDR235W | *prp42-ts* | 14.02089385 | 3.012337082 | 2.543604026 |  | 5.982118573 | 14.02089385 |
| YPR082C | *dib1-ts* | 13.60808292 | 4.149312557 | 3.862449396 | 2.59238035 | 4.770765376 | 13.60808292 |
| YBL026W | *lsm2-ts* | 13.26351297 | 4.426682341 | 4.813062639 | 2.627553094 | 3.767290469 | 13.26351297 |
| YDR416W | *syf1-ts* | 12.86804869 | 4.923986208 | 4.378028053 | 2.784352923 | 5.111411803 | 12.86804869 |
| YDR378C | *lsm6Δ* | 12.55281276 | 3.097391317 | 4.215994301 | 2.499824745 | 4.37257837 | 12.55281276 |
| YER013W | *prp22-1* | 11.5555972 | 4.957758493 | 5.119803383 | 3.779590519 | 6.80466963 | 11.5555972 |
| YBR152W | *spp381-ts* | 11.08632319 | 4.477375923 | 4.239990522 | 2.619408398 | 3.986992389 | 11.08632319 |
| YKL173W | *snu114-12* | 10.69376914 | 4.429189449 | 4.048280611 | 2.965286274 | 5.234218453 | 10.69376914 |
| YPR057W | *brr1Δ* | 10.42686534 | 2.203224764 | 2.03131637 |  | 4.347145418 | 10.42686534 |
| YNL147W | *lsm7Δ* | 10.42394921 |  | 2.126407209 |  |  | 10.42394921 |
| YGL174W | *bud13Δ* | 10.37568127 | 4.022942091 | 4.412894465 |  | 7.49812796 | 10.37568127 |
| YMR314W | *pre5-ts* | 10.05612936 | 4.031213003 | 2.523245052 | 5.545743259 | 4.11558963 | 10.05612936 |
| YMR288W | *hsh155-ts* | 9.712928133 | 3.060988597 |  |  | 3.108214196 | 9.712928133 |
| YPL157W | *tgs1Δ* | 8.782079355 |  |  |  | 2.511207622 | 8.782079355 |
| YKL012W | *prp40-ts* | 8.368141374 |  | 3.239767313 |  | 5.028092485 | 8.368141374 |
| YGR013W | *snu71-ts* | 7.700324634 | 2.794835862 | 2.144641019 |  | 3.402527947 | 7.700324634 |
| YGR006W | *prp18Δ* | 3.956584683 | 7.595808504 |  | 2.516997744 |  | 7.595808504 |
| YGR093W | *ygr093wΔ* | 7.506240194 |  |  |  |  | 7.506240194 |
| YBL074C | *aar2-ts* | 7.113384844 | 3.481247792 | 3.637293142 | 2.087499564 | 3.598032065 | 7.113384844 |
| YNR004W | *ynr004wΔ* | 6.90192639 | 2.289738306 | 2.820060193 |  | 5.109669169 | 6.90192639 |
| YDL209C | *cwc2-ts* | 6.34462198 | 2.763591739 | 2.79101552 |  | 3.702469167 | 6.34462198 |
| YOR148C | *spp2-ts* | 5.739276802 | 3.418111091 | 3.08527119 | 5.985583064 | 4.552646661 | 5.985583064 |
| YPR107C | *yth1-ts* | 5.973739462 |  |  | 2.088352845 | 4.469595548 | 5.973739462 |
| YLR115W | *cft2-ts* | 5.909021756 |  |  |  | 2.70946699 | 5.909021756 |
| YOR308C | *snu66Δ* | 5.805020495 | 3.424851068 | 3.903860699 | 3.346285121 | 5.61012194 | 5.805020495 |
| YKR040C | *ykr040cΔ* | 5.479704399 |  | 2.243623672 |  | 2.39291999 | 5.479704399 |
| YJL203W | *prp21-1* | 5.462688252 |  |  |  | 3.732162279 | 5.462688252 |
| YPR079W | *mrl1Δ* |  |  | 5.353288287 |  |  | 5.353288287 |
| YKR041W | *ykr041wΔ* | 5.321112482 |  |  |  | 2.594554778 | 5.321112482 |
| YOL121C | *rps19aΔ* |  |  | 5.231660389 |  |  | 5.231660389 |
| YOL119C | *mch4Δ* |  |  | 5.159384252 |  |  | 5.159384252 |
| YCR063W | *bud31Δ* | 4.317653194 | 3.791448355 |  | 2.119265513 | 5.147896353 | 5.147896353 |
| YPR062W | *fcy1Δ* |  |  | 5.112023463 |  |  | 5.112023463 |
| YPR047W | *msf1Δ* |  |  | 4.989530372 |  |  | 4.989530372 |
| YDR485C | *vps72Δ* | 4.745523478 |  |  |  | 2.05787566 | 4.745523478 |
| YBR232C | *ybr232cΔ* | 4.155674184 |  | 4.624713437 |  |  | 4.624713437 |
| YGR201C | *ygr201cΔ* |  |  | 4.602227576 |  |  | 4.602227576 |
| YPR065W | *rox1Δ* |  |  | 4.578386409 |  |  | 4.578386409 |
| YGR188C | *bub1Δ* | 2.374475069 |  | 4.573037425 |  |  | 4.573037425 |
| YML127W | *rsc9-ts* | 4.56414331 |  |  |  |  | 4.56414331 |
| YPR063C | *ypr063cΔ* |  |  | 4.555272043 |  |  | 4.555272043 |
| YDR241W | *bud26Δ* |  | 3.192278865 |  |  | 4.437450766 | 4.437450766 |
| YOL064C | *met22Δ* | 4.413596235 |  |  |  |  | 4.413596235 |
| YOL118C | *yol118cΔ* |  |  | 4.35226373 |  |  | 4.35226373 |
| YBR119W | *mud1Δ* |  |  | 2.087336715 |  | 4.304903548 | 4.304903548 |
| YPL173W | *mrpl40Δ* | 4.288041008 |  |  |  |  | 4.288041008 |
| YLR357W | *rsc2Δ* | 4.251811526 |  | 2.986628897 |  |  | 4.251811526 |
| YGR129W | *syf2Δ* | 4.200739583 |  |  |  |  | 4.200739583 |
| YNL107W | *yaf9Δ* | 4.195451284 |  |  |  |  | 4.195451284 |
| YMR091C | *npl6Δ* | 4.148514544 |  |  |  |  | 4.148514544 |
| YIL061C | *snp1-ts* | 3.53696104 |  |  |  | 4.142906222 | 4.142906222 |
| YLR399C | *bdf1Δ* | 3.920257531 | 2.122363982 | 4.141264625 |  |  | 4.141264625 |
| YAL016W | *tpd3Δ* |  |  |  | 4.117393474 |  | 4.117393474 |
| YML090W | *yml090wΔ* |  |  | 4.082819967 |  |  | 4.082819967 |
| YML074C | *fpr3Δ* |  |  | 4.074084676 |  |  | 4.074084676 |
| YBR011C | *ipp1-ts* |  |  |  |  | 4.044818612 | 4.044818612 |
| YDR443C | *ssn2Δ* |  | 2.10221244 | 3.992609183 |  | 2.189904326 | 3.992609183 |
| YKL074C | *mud2Δ* |  | 3.120156268 | 2.103149765 |  | 3.857403331 | 3.857403331 |
| YOL117W | *rri2Δ* |  |  | 3.834137636 |  |  | 3.834137636 |
| YKL095W | *yju2-ts* | 3.796871098 |  |  |  |  | 3.796871098 |
| YKR086W | *prp23-1* | 3.784775548 | 2.714894165 | 2.092016708 | 2.18758359 | 2.576667149 | 3.784775548 |
| YDR243C | *prp28-1* |  | 2.206927313 | 2.93505319 | 3.763483972 | 3.200271288 | 3.763483972 |
| YNL059C | *arp5Δ* | 3.760554654 |  |  |  |  | 3.760554654 |
| YER096W | *shc1Δ* |  |  | 3.75182496 |  |  | 3.75182496 |
| YLR403W | *sfp1Δ* |  |  |  | 2.751774648 | 3.747418255 | 3.747418255 |
| YPR189W | *ski3Δ* |  | 3.707167289 | 2.061826593 |  |  | 3.707167289 |
| YDR081C | *pdc2-ts* | 3.687426146 |  |  |  |  | 3.687426146 |
| YHR005C | *gpa1Δ* |  |  | 3.675082054 |  |  | 3.675082054 |
| YML092C | *pre8-ts* |  |  |  |  | 3.541715322 | 3.541715322 |
| YHR140W | *yhr140wΔ* |  |  | 3.538077004 |  |  | 3.538077004 |
| YMR167W | *mlh1Δ* | 3.53299276 |  |  |  |  | 3.53299276 |
| YOL145C | *ctr9Δ* | 3.525792974 | 2.124797614 |  |  |  | 3.525792974 |
| YDR462W | *mrpl28Δ* |  |  | 3.521224302 |  |  | 3.521224302 |
| YPR046W | *mcm16Δ* |  |  | 3.502719401 |  |  | 3.502719401 |
| YCR069W | *cpr4Δ* |  |  | 3.47029275 |  |  | 3.47029275 |
| YGR064W | *ygr064wΔ* | 3.448142936 |  |  |  |  | 3.448142936 |
| YOR141C | *arp8Δ* | 3.436853175 |  |  |  |  | 3.436853175 |
| YHR026W | *ppa1Δ* |  |  | 3.419124983 |  |  | 3.419124983 |
| YOR096W | *rps7aΔ* |  |  | 3.412331303 |  |  | 3.412331303 |
| YJR093C | *fip1-ts* | 3.410160119 |  |  |  |  | 3.410160119 |
| YAL044W-A | *yal044w-aΔ* |  |  | 3.400784234 |  |  | 3.400784234 |
| YPR083W | *mdm36Δ* | 3.373322098 |  |  |  |  | 3.373322098 |
| YOL021C | *dis3-ts* | 3.08060329 |  |  |  | 3.369112166 | 3.369112166 |
| YML112W | *ctk3Δ* | 2.636579442 |  |  |  | 3.367304359 | 3.367304359 |
| YOR317W | *faa1Δ* | 3.36493781 |  | 2.355474339 |  | 2.073580027 | 3.36493781 |
| YML073C | *rpl6aΔ* |  |  | 3.297329872 |  |  | 3.297329872 |
| YFR021W | *atg18Δ* | 3.268994117 |  |  |  |  | 3.268994117 |
| YER093C-A | *yer093c-aΔ* |  |  | 3.267797648 |  |  | 3.267797648 |
| YGL215W | *clg1Δ* |  |  | 2.116574072 |  | 3.266753113 | 3.266753113 |
| YAL051W | *oaf1Δ* |  |  | 3.265892615 |  |  | 3.265892615 |
| YOR362C | *pre10-ts* |  |  |  |  | 3.247628286 | 3.247628286 |
| YKR099C-A | *ykr099c-aΔ* |  | 3.246962192 |  |  | 2.85354944 | 3.246962192 |
| YJL045W | *yjl045wΔ* |  |  | 3.236295228 |  |  | 3.236295228 |
| YCL014W | *bud3Δ* |  | 2.096774502 | 3.234343613 |  |  | 3.234343613 |
| YDR334W | *swr1Δ* | 3.233635039 |  |  |  |  | 3.233635039 |
| YNL296W | *ynl296wΔ* | 3.232620115 |  |  | 2.349246756 |  | 3.232620115 |
| YOL090W | *msh2Δ* | 3.223225456 |  |  |  |  | 3.223225456 |
| YLR358C | *ylr358cΔ* | 2.799126937 |  | 3.213457313 |  |  | 3.213457313 |
| YNR032C-A | *hub1Δ* | 3.196406996 |  |  |  |  | 3.196406996 |
| YNR011C | *prp2-1* | 3.157345904 | 2.20677852 | 2.208479469 | 3.175597221 | 2.304013136 | 3.175597221 |
| YLL006W-A | *yll006w-aΔ* |  | 3.163057778 |  |  |  | 3.163057778 |
| YMR206W | *ymr206wΔ* | 3.158749429 |  |  |  |  | 3.158749429 |
| YBR049C | *reb1-ts* |  |  | 3.156258781 |  |  | 3.156258781 |
| YJR083C | *acf4Δ* |  |  | 3.150492012 |  |  | 3.150492012 |
| YFR019W | *fab1Δ* |  |  | 3.150350979 |  |  | 3.150350979 |
| YMR119W-A | *ymr119w-aΔ* |  |  | 3.146632235 |  |  | 3.146632235 |
| YPL205C | *ypl205cΔ* | 3.142285872 |  |  |  |  | 3.142285872 |
| YAR042W | *swh1Δ* |  | 2.042325282 | 3.14131985 |  | 2.134438711 | 3.14131985 |
| YBR294W | *sul1Δ* |  | 2.440596809 | 3.131276358 |  |  | 3.131276358 |
| YLR085C | *arp6Δ* | 3.129119328 |  | 2.135544864 |  |  | 3.129119328 |
| YGR134W | *caf130Δ* | 3.122958839 |  |  |  |  | 3.122958839 |
| YFR030W | *met10Δ* |  |  | 3.121416689 |  |  | 3.121416689 |
| YFL001W | *deg1Δ* |  |  |  |  | 3.100537935 | 3.100537935 |
| YGL129C | *rsm23Δ* |  | 2.150722267 | 2.321952099 |  | 3.094932823 | 3.094932823 |
| YNL215W | *ies2Δ* | 3.091323329 |  |  |  |  | 3.091323329 |
| YMR031W-A | *ymr031w-aΔ* | 3.08997833 |  |  |  |  | 3.08997833 |
| YOR001W | *rrp6Δ* | 3.078632688 |  |  |  | 2.837432435 | 3.078632688 |
| YOL141W | *ppm2Δ* |  |  | 3.058235715 |  |  | 3.058235715 |
| YJR050W | *isy1Δ* | 3.038648671 |  | 2.367734359 |  |  | 3.038648671 |
| YML102W | *cac2Δ* |  |  | 3.029305646 |  |  | 3.029305646 |
| YDR195W | *ref2Δ* |  |  |  |  | 3.023721934 | 3.023721934 |
| YLR292C | *sec72Δ* |  |  | 2.898961002 |  | 3.019570292 | 3.019570292 |
| YOR128C | *ade2Δ* |  | 2.138222864 | 3.014509216 |  | 2.804728228 | 3.014509216 |
| YLR392C | *ylr392cΔ* | 3.006348539 |  |  |  |  | 3.006348539 |
| YKR081C | *rpf2-ts* | 2.993832396 |  |  |  |  | 2.993832396 |
| YOR150W | *mrpl23Δ* |  | 2.220877277 | 2.988455116 |  | 2.66836564 | 2.988455116 |
| YOR275C | *rim20Δ* |  |  | 2.980788499 |  |  | 2.980788499 |
| YDR083W | *rrp8Δ* |  |  |  |  | 2.967043141 | 2.967043141 |
| YLR062C | *bud28Δ* | 2.962598691 |  |  |  |  | 2.962598691 |
| YKR082W | *nup133Δ* |  |  |  | 2.956584752 | 2.274576811 | 2.956584752 |
| YGR072W | *upf3Δ* |  |  | 2.956548544 |  | 2.385241022 | 2.956548544 |
| YOL137W | *bsc6Δ* |  |  | 2.947741629 |  |  | 2.947741629 |
| YDR067C | *oca6Δ* |  |  | 2.946098371 |  | 2.306793472 | 2.946098371 |
| YJL116C | *nca3Δ* |  |  | 2.941236968 |  |  | 2.941236968 |
| YNL133C | *fyv6Δ* | 2.644834445 | 2.927176666 |  | 2.54358968 |  | 2.927176666 |
| YDR363W-A | *sem1Δ* | 2.564574075 |  |  |  | 2.90955476 | 2.90955476 |
| YAL067W-A | *yal067w-aΔ* |  |  | 2.906548274 |  |  | 2.906548274 |
| YBR231C | *swc5Δ* | 2.901538478 |  |  |  | 2.026739839 | 2.901538478 |
| YIL104C | *shq1-ts* | 2.899139248 |  |  |  |  | 2.899139248 |
| YCR062W | *ycr062wΔ* |  | 2.895229755 | 2.740910634 |  |  | 2.895229755 |
| YPL066W | *ypl066wΔ* |  | 2.184825014 | 2.888836417 |  |  | 2.888836417 |
| YJL161W | *fmp33Δ* | 2.885645618 |  |  |  | 2.335920862 | 2.885645618 |
| YPR159W | *kre6Δ* |  |  |  | 2.881486401 |  | 2.881486401 |
| YPL061W | *ald6Δ* |  |  | 2.880612384 |  |  | 2.880612384 |
| YHL039W | *yhl039wΔ* | 2.86987706 |  |  |  |  | 2.86987706 |
| YJL047C | *rtt101Δ* |  |  | 2.860445634 |  |  | 2.860445634 |
| YPL155C | *kip2Δ* | 2.859851196 |  |  |  |  | 2.859851196 |
| YOR143C | *thi80-ts* |  |  | 2.856286911 |  |  | 2.856286911 |
| YCR067C | *sed4Δ* |  |  | 2.842378103 |  |  | 2.842378103 |
| YAL021C | *ccr4Δ* |  |  | 2.840629348 |  |  | 2.840629348 |
| YGL066W | *sgf73Δ* | 2.838273668 |  |  |  |  | 2.838273668 |
| YDL116W | *nup84Δ* | 2.821381119 |  |  |  | 2.742993534 | 2.821381119 |
| YML084W | *yml084wΔ* |  |  | 2.157320451 | 2.452774147 | 2.801373352 | 2.801373352 |
| YNL055C | *por1Δ* |  |  | 2.800568778 |  |  | 2.800568778 |
| YFR050C | *pre4-ts* |  |  |  |  | 2.798960127 | 2.798960127 |
| YLR222C | *utp13-ts* |  |  | 2.794314601 |  |  | 2.794314601 |
| YGL214W | *ygl214wΔ* |  | 2.784501503 |  |  |  | 2.784501503 |
| YDR485C | *vps72Δ* | 2.782236832 |  |  |  |  | 2.782236832 |
| YLR394W | *cst9Δ* |  |  | 2.773048317 |  |  | 2.773048317 |
| YBL058W | *shp1Δ* | 2.756248787 |  |  |  |  | 2.756248787 |
| YLR424W | *spp382-ts* | 2.754538687 |  |  |  |  | 2.754538687 |
| YOL004W | *sin3Δ* |  |  | 2.748878221 |  | 2.741497323 | 2.748878221 |
| YPR049C | *atg11Δ* |  |  |  |  | 2.730959763 | 2.730959763 |
| YLR048W | *rps0bΔ* | 2.729760543 |  |  |  |  | 2.729760543 |
| YGL173C | *kem1Δ* |  |  | 2.543591801 | 2.551289076 | 2.727078797 | 2.727078797 |
| YDL030W | *prp9-1* | 2.726127408 | 2.024936181 |  | 2.209572623 | 2.334473094 | 2.726127408 |
| YLR244C | *map1Δ* |  |  |  | 2.717643286 |  | 2.717643286 |
| YPL189W | *gup2Δ* | 2.709326256 |  |  |  |  | 2.709326256 |
| YCR048W | *are1Δ* |  |  | 2.708893744 |  |  | 2.708893744 |
| YPL129W | *taf14Δ* | 2.704294051 |  |  |  |  | 2.704294051 |
| YNL177C | *mrpl22Δ* |  | 2.309928082 | 2.206022905 | 2.701500276 | 2.284057772 | 2.701500276 |
| YPR154W | *pin3Δ* | 2.701095703 |  |  |  |  | 2.701095703 |
| YJL046W | *aim22Δ* |  |  | 2.699326668 |  |  | 2.699326668 |
| YKL057C | *nup120Δ* |  |  |  | 2.624524378 | 2.698186824 | 2.698186824 |
| YOL079W | *yol079wΔ* | 2.696866637 |  |  |  |  | 2.696866637 |
| YOR160W | *mtr10-ts* |  |  |  |  | 2.693682665 | 2.693682665 |
| YLR391W | *ylr391wΔ* |  |  |  |  | 2.68999916 | 2.68999916 |
| YLR153C | *acs2-ts* |  |  |  | 2.681488568 |  | 2.681488568 |
| YDR074W | *tps2Δ* |  | 2.667116078 | 2.501809271 |  |  | 2.667116078 |
| YDR068W | *dos2Δ* |  |  | 2.665659835 |  | 2.155887122 | 2.665659835 |
| YLR311C | *ylr311cΔ* |  |  | 2.664521574 |  |  | 2.664521574 |
| YDR341C | *ydr341c-ts* | 2.659663017 |  |  |  |  | 2.659663017 |
| YBR072C-A | *ybr072c-aΔ* |  |  | 2.656470819 |  |  | 2.656470819 |
| YER175W-A | *yer175w-aΔ* |  |  | 2.073158942 |  | 2.647099125 | 2.647099125 |
| YLR204W | *qri5Δ* |  |  | 2.641252603 |  | 2.61009893 | 2.641252603 |
| YLR417W | *vps36Δ* |  |  | 2.640380136 |  |  | 2.640380136 |
| YDL047W | *sit4Δ* |  |  | 2.639484316 |  | 2.209077921 | 2.639484316 |
| YBL033C | *rib1Δ* |  |  | 2.634665398 |  |  | 2.634665398 |
| YFR031C-A | *rpl2aΔ* |  |  |  |  | 2.632034266 | 2.632034266 |
| YHR041C | *srb2Δ* |  |  | 2.630343102 |  |  | 2.630343102 |
| YCL026C-A | *frm2Δ* |  |  | 2.620668284 |  |  | 2.620668284 |
| YLR132C | *ylr132c-ts* | 2.602211921 |  |  |  |  | 2.602211921 |
| YOL138C | *rtc1Δ* |  |  | 2.597063659 |  |  | 2.597063659 |
| YLR174W | *idp2Δ* |  |  | 2.577777406 |  | 2.155203896 | 2.577777406 |
| YPR045C | *ypr045cΔ* |  |  | 2.577096722 |  |  | 2.577096722 |
| YGL213C | *ski8Δ* |  | 2.573632501 |  |  |  | 2.573632501 |
| YLR185W | *rpl37aΔ* |  |  | 2.572060429 |  | 2.260251241 | 2.572060429 |
| YLR017W | *meu1Δ* | 2.568380948 |  |  |  |  | 2.568380948 |
| YMR071C | *tvp18Δ* |  |  | 2.562322193 |  |  | 2.562322193 |
| YML041C | *vps71Δ* | 2.558798949 |  |  |  |  | 2.558798949 |
| YPL064C | *cwc27Δ* |  |  | 2.555913976 |  | 2.417655911 | 2.555913976 |
| YEL044W | *ies6Δ* | 2.483551486 |  | 2.20232173 |  | 2.53857113 | 2.53857113 |
| YBR128C | *atg14Δ* |  |  | 2.537228755 |  |  | 2.537228755 |
| YPL137C | *gip3Δ* |  |  | 2.526703457 |  |  | 2.526703457 |
| YLR418C | *cdc73Δ* | 2.521742401 |  |  |  |  | 2.521742401 |
| YMR032W | *hof1Δ* |  |  | 2.099328511 |  | 2.513480685 | 2.513480685 |
| YBR289W | *snf5Δ* |  |  |  | 2.512678372 |  | 2.512678372 |
| YBR177C | *eht1Δ* |  |  | 2.378821499 |  | 2.511133944 | 2.511133944 |
| YLR219W | *msc3Δ* |  |  | 2.508583789 |  | 2.472031462 | 2.508583789 |
| YAL053W | *flc2Δ* |  |  | 2.501267055 |  | 2.049160675 | 2.501267055 |
| YER175C | *tmt1Δ* |  |  |  |  | 2.491465416 | 2.491465416 |
| YAL049C | *aim2Δ* |  |  | 2.28355054 |  | 2.487417511 | 2.487417511 |
| YPL052W | *oaz1Δ* |  | 2.194573386 | 2.486324087 |  |  | 2.486324087 |
| YPL133C | *rds2Δ* | 2.483304185 |  |  |  |  | 2.483304185 |
| YJL095W | *bck1Δ* |  |  | 2.480138138 |  |  | 2.480138138 |
| YBR129C | *opy1Δ* | 2.476991964 |  |  |  |  | 2.476991964 |
| YKL046C | *dcw1Δ* |  |  | 2.039232711 |  | 2.472786305 | 2.472786305 |
| YLR175W | *cbf5-ts* | 2.470021327 |  |  |  |  | 2.470021327 |
| YGL043W | *dst1Δ* | 2.460418196 |  |  |  |  | 2.460418196 |
| YBR189W | *rps9bΔ* |  |  | 2.459739634 |  |  | 2.459739634 |
| YPL116W | *hos3Δ* | 2.458632847 |  |  |  |  | 2.458632847 |
| YHR077C | *nmd2Δ* |  |  | 2.456815758 |  |  | 2.456815758 |
| YLL007C | *yll007cΔ* | 2.456606591 |  |  |  |  | 2.456606591 |
| YGL091C | *nbp35-ts* | 2.453614879 |  |  |  |  | 2.453614879 |
| YLR200W | *yke2Δ* |  |  | 2.105685921 |  | 2.450374178 | 2.450374178 |
| YAL016C-B | *yal016c-bΔ* |  | 2.077287188 | 2.449802607 |  |  | 2.449802607 |
| YGR118W | *rps23aΔ* | 2.443805909 |  |  |  |  | 2.443805909 |
| YDR240C | *snu56-ts* | 2.436568479 |  |  |  | 2.066871103 | 2.436568479 |
| YJL206C-A | *yjl206c-aΔ* |  |  |  |  | 2.435051207 | 2.435051207 |
| YOR187W | *tuf1Δ* | 2.430737277 |  |  |  |  | 2.430737277 |
| YHR064C | *ssz1Δ* |  |  | 2.430658938 |  |  | 2.430658938 |
| YIL076W | *sec28Δ* |  | 2.191257106 |  | 2.426754246 |  | 2.426754246 |
| YLR422W | *ylr422wΔ* |  |  | 2.415262903 |  |  | 2.415262903 |
| YHR193C | *egd2Δ* | 2.413112106 |  |  |  |  | 2.413112106 |
| YNL225C | *cnm67Δ* |  |  |  | 2.411834404 |  | 2.411834404 |
| YMR218C | *trs130-ts* | 2.411314199 |  |  |  |  | 2.411314199 |
| YDL069C | *cbs1Δ* |  |  |  |  | 2.410305086 | 2.410305086 |
| YEL053C | *mak10Δ* |  |  |  |  | 2.40994653 | 2.40994653 |
| YNL227C | *jjj1Δ* | 2.409516778 |  |  |  |  | 2.409516778 |
| YGR056W | *rsc1Δ* | 2.398608213 |  |  |  |  | 2.398608213 |
| YBR301W | *dan3Δ* |  |  | 2.395268525 |  |  | 2.395268525 |
| YGL186C | *tpn1Δ* |  |  | 2.391286919 |  | 2.261390779 | 2.391286919 |
| YKR022C | *ntr2-ts* | 2.389693177 |  |  |  |  | 2.389693177 |
| YNL315C | *atp11Δ* |  |  | 2.38942185 |  |  | 2.38942185 |
| YBR127C | *vma2Δ* |  |  | 2.386522066 |  |  | 2.386522066 |
| YHR150W | *pex28Δ* | 2.383771399 |  |  |  |  | 2.383771399 |
| YBR173C | *ump1Δ* |  |  |  |  | 2.382680456 | 2.382680456 |
| YJL006C | *ctk2Δ* |  | 2.380811531 |  |  |  | 2.380811531 |
| YJL102W | *mef2Δ* | 2.378150494 |  |  |  |  | 2.378150494 |
| YGR281W | *yor1Δ* |  |  | 2.37428597 |  |  | 2.37428597 |
| YBR196C-A | *ybr196c-aΔ* |  |  |  | 2.370950024 |  | 2.370950024 |
| YML007C-A | *yml007c-aΔ* |  |  | 2.27695331 |  | 2.369496171 | 2.369496171 |
| YPR152C | *urn1Δ* |  |  |  |  | 2.362515083 | 2.362515083 |
| YEL022W | *gea2Δ* |  |  |  |  | 2.361683722 | 2.361683722 |
| YDR472W | *trs31-ts* |  |  |  | 2.361552383 |  | 2.361552383 |
| YPL194W | *ddc1Δ* |  |  | 2.360548721 |  |  | 2.360548721 |
| YIL006W | *yia6Δ* |  |  | 2.358972547 |  |  | 2.358972547 |
| YDR261C | *exg2Δ* |  |  | 2.358178062 |  |  | 2.358178062 |
| YOR089C | *vps21Δ* |  |  |  |  | 2.356215951 | 2.356215951 |
| YPR030W | *csr2Δ* |  |  |  |  | 2.350976964 | 2.350976964 |
| YGL146C | *rrt6Δ* |  |  | 2.216348174 |  | 2.349945588 | 2.349945588 |
| YER161C | *spt2Δ* |  |  |  |  | 2.347397313 | 2.347397313 |
| YDR433W | *ydr433wΔ* |  |  | 2.076625356 |  | 2.341768043 | 2.341768043 |
| YOL160W | *yol160wΔ* | 2.340891186 |  |  |  |  | 2.340891186 |
| YFL033C | *rim15Δ* |  |  |  |  | 2.335325098 | 2.335325098 |
| YER155C | *bem2Δ* |  |  | 2.335174658 |  |  | 2.335174658 |
| YMR172C-A | *ymr172c-aΔ* | 2.330568533 |  |  |  |  | 2.330568533 |
| YOL052C-A | *ddr2Δ* |  |  |  |  | 2.32777904 | 2.32777904 |
| YMR035W | *imp2Δ* |  |  |  |  | 2.324881747 | 2.324881747 |
| YNL300W | *tos6Δ* |  |  |  |  | 2.321696378 | 2.321696378 |
| YER187W | *yer187wΔ* |  |  | 2.16162016 |  | 2.318057568 | 2.318057568 |
| YDR475C | *jip4Δ* | 2.314508423 |  |  |  |  | 2.314508423 |
| YLR371W | *rom2Δ* |  |  | 2.207886336 |  | 2.314497788 | 2.314497788 |
| YPR191W | *qcr2Δ* |  |  | 2.313790138 |  | 2.100262814 | 2.313790138 |
| YLR309C | *imh1Δ* |  | 2.306847128 |  |  |  | 2.306847128 |
| YDL077C | *vam6Δ* |  |  | 2.303716226 |  |  | 2.303716226 |
| YBL046W | *psy4Δ* |  |  |  |  | 2.303038101 | 2.303038101 |
| YJL151C | *sna3Δ* | 2.298862809 |  |  |  |  | 2.298862809 |
| YOR288C | *mpd1Δ* |  |  |  |  | 2.296981015 | 2.296981015 |
| YHR120W | *msh1Δ* | 2.294454219 |  |  |  |  | 2.294454219 |
| YOR076C | *ski7Δ* |  | 2.292473798 |  |  |  | 2.292473798 |
| YGR047C | *tfc4-ts* | 2.289942208 |  |  |  |  | 2.289942208 |
| YHL005C | *yhl005cΔ* |  |  |  |  | 2.289702013 | 2.289702013 |
| YPR106W | *isr1Δ* | 2.288819949 |  |  |  |  | 2.288819949 |
| YLR354C | *tal1Δ* |  |  | 2.288714046 |  |  | 2.288714046 |
| YLR012C | *ylr012cΔ* |  |  |  |  | 2.288375029 | 2.288375029 |
| YPL222W | *fmp40Δ* |  |  |  | 2.288301483 |  | 2.288301483 |
| YGL045W | *rim8Δ* |  |  | 2.286794523 |  | 2.180360869 | 2.286794523 |
| YOR099W | *ktr1Δ* | 2.286792471 |  |  |  |  | 2.286792471 |
| YFR033C | *qcr6Δ* | 2.282540366 |  |  |  |  | 2.282540366 |
| YNL293W | *msb3Δ* |  |  | 2.267336438 |  |  | 2.267336438 |
| YMR316W | *dia1Δ* |  | 2.25942415 |  |  |  | 2.25942415 |
| YDR099W | *bmh2Δ* |  |  |  |  | 2.258933945 | 2.258933945 |
| YBL003C | *hta2Δ* |  |  | 2.258782944 |  | 2.146614309 | 2.258782944 |
| YDR148C | *kgd2Δ* |  | 2.257948754 |  |  |  | 2.257948754 |
| YMR214W | *scj1Δ* | 2.255223459 |  |  |  |  | 2.255223459 |
| YEL067C | *yel067cΔ* |  |  | 2.254494031 |  | 2.089268504 | 2.254494031 |
| YGL258W | *vel1Δ* | 2.250637596 |  |  |  |  | 2.250637596 |
| YGR121W-A | *ygr121w-aΔ* |  |  |  |  | 2.250058749 | 2.250058749 |
| YLR262C-A | *tma7Δ* |  |  | 2.248563673 |  | 2.065804622 | 2.248563673 |
| YER123W | *yck3Δ* |  |  |  |  | 2.243943225 | 2.243943225 |
| YHR049C-A | *yhr049c-aΔ* |  |  | 2.242125716 |  |  | 2.242125716 |
| YJL117W | *pho86Δ* |  |  | 2.238224544 |  |  | 2.238224544 |
| YBL038W | *mrpl16Δ* |  |  | 2.235099311 |  |  | 2.235099311 |
| YLR129W | *dip2-ts* |  |  | 2.231568372 |  |  | 2.231568372 |
| YOL152W | *fre7Δ* |  |  | 2.229144361 |  | 2.052186586 | 2.229144361 |
| YKR096W | *ykr096wΔ* | 2.226344574 |  |  |  |  | 2.226344574 |
| YDL088C | *asm4Δ* |  |  | 2.226307955 |  |  | 2.226307955 |
| YOR274W | *mod5Δ* |  |  | 2.225047262 |  |  | 2.225047262 |
| YGL199C | *ygl199cΔ* |  |  | 2.108933711 |  | 2.219407031 | 2.219407031 |
| YHR147C | *mrpl6Δ* |  | 2.21902438 |  |  |  | 2.21902438 |
| YGL262W | *ygl262wΔ* | 2.218009463 |  |  |  |  | 2.218009463 |
| YOR153W | *pdr5Δ* |  |  | 2.217025119 |  | 2.169520235 | 2.217025119 |
| YLR173W | *ylr173wΔ* |  | 2.215601254 |  |  |  | 2.215601254 |
| YBL091C-A | *scs22Δ* |  |  |  |  | 2.215200223 | 2.215200223 |
| YIL077C | *yil077cΔ* |  |  | 2.213272347 |  |  | 2.213272347 |
| YMR111C | *ymr111cΔ* | 2.213008256 |  |  |  |  | 2.213008256 |
| YPL018W | *ctf19Δ* |  |  |  |  | 2.212733885 | 2.212733885 |
| YOL096C | *coq3Δ* |  |  | 2.211966094 |  |  | 2.211966094 |
| YIL072W | *hop1Δ* |  |  |  |  | 2.208242119 | 2.208242119 |
| YPR051W | *mak3Δ* |  |  |  |  | 2.207768065 | 2.207768065 |
| YLL044W | *yll044wΔ* | 2.206610285 |  |  |  |  | 2.206610285 |
| YBR085W | *aac3Δ* |  |  | 2.205146575 |  |  | 2.205146575 |
| YNL032W | *siw14Δ* |  |  | 2.202844053 |  |  | 2.202844053 |
| YBL005W | *pdr3Δ* |  |  | 2.202148236 |  |  | 2.202148236 |
| YLL057C | *jlp1Δ* |  |  | 2.201163166 |  |  | 2.201163166 |
| YER030W | *chz1Δ* |  |  |  |  | 2.1987568 | 2.1987568 |
| YDR478W | *snm1-ts* |  |  |  |  | 2.197383491 | 2.197383491 |
| YER110C | *kap123Δ* |  |  | 2.195484481 |  | 2.088914939 | 2.195484481 |
| YFR025C | *his2Δ* |  |  | 2.192595924 |  |  | 2.192595924 |
| YOL016C | *cmk2Δ* |  |  | 2.190492377 |  | 2.035388637 | 2.190492377 |
| YBR094W | *pby1Δ* | 2.190008781 |  |  |  |  | 2.190008781 |
| YLR226W | *bur2Δ* |  |  |  |  | 2.185938619 | 2.185938619 |
| YDR286C | *ydr286cΔ* |  |  |  |  | 2.180675715 | 2.180675715 |
| YKL033W-A | *ykl033w-aΔ* |  |  | 2.179067456 |  |  | 2.179067456 |
| YBR015C | *mnn2Δ* |  |  | 2.177582672 |  |  | 2.177582672 |
| YDR043C | *nrg1Δ* |  | 2.176245792 |  |  |  | 2.176245792 |
| YPR164W | *mms1Δ* |  |  | 2.175525931 |  |  | 2.175525931 |
| YJL206C | *yjl206cΔ* |  |  |  |  | 2.17450151 | 2.17450151 |
| YMR179W | *spt21Δ* |  |  |  | 2.174199812 |  | 2.174199812 |
| YGL005C | *cog7Δ* | 2.173874755 |  |  |  |  | 2.173874755 |
| YBR065C | *ecm2Δ* | 2.17255342 |  |  |  |  | 2.17255342 |
| YCL057C-A | *ycl057c-aΔ* |  |  | 2.168865718 |  |  | 2.168865718 |
| YOL093W | *trm10Δ* |  |  |  | 2.168269051 | 2.063217009 | 2.168269051 |
| YDL056W | *mbp1Δ* |  |  |  |  | 2.166694905 | 2.166694905 |
| YGL175C | *sae2Δ* |  |  |  |  | 2.16515297 | 2.16515297 |
| YLR340W | *rpp0-ts* | 2.164579804 |  |  |  |  | 2.164579804 |
| YNL096C | *rps7bΔ* |  |  | 2.164328133 |  |  | 2.164328133 |
| YOR138C | *rup1Δ* |  | 2.156776091 |  |  |  | 2.156776091 |
| YKL064W | *mnr2Δ* |  | 2.156759568 |  |  |  | 2.156759568 |
| YLR398C | *ski2Δ* |  | 2.156261276 |  |  |  | 2.156261276 |
| YFL016C | *mdj1Δ* |  |  |  |  | 2.150342374 | 2.150342374 |
| YNL162W | *rpl42aΔ* |  |  | 2.107187226 |  | 2.150194246 | 2.150194246 |
| YKL063C | *ykl063cΔ* |  | 2.148224586 |  |  |  | 2.148224586 |
| YKL062W | *msn4Δ* |  |  |  |  | 2.142073443 | 2.142073443 |
| YLR189C | *atg26Δ* |  |  | 2.140514984 |  |  | 2.140514984 |
| YDR447C | *rps17bΔ* |  |  | 2.13568154 |  |  | 2.13568154 |
| YER011W | *tir1Δ* |  |  |  |  | 2.129145111 | 2.129145111 |
| YAL036C | *rbg1Δ* |  |  |  |  | 2.128698459 | 2.128698459 |
| YDR296W | *mhr1Δ* |  |  |  |  | 2.123766282 | 2.123766282 |
| YKL184W | *spe1Δ* |  | 2.121596512 |  |  |  | 2.121596512 |
| YDR200C | *vps64Δ* |  |  |  |  | 2.120067021 | 2.120067021 |
| YPR188C | *mlc2Δ* |  | 2.119737883 | 2.083168315 |  |  | 2.119737883 |
| YLR218C | *ylr218cΔ* |  |  | 2.11441872 |  |  | 2.11441872 |
| YBR284W | *ybr284wΔ* |  |  |  |  | 2.105856334 | 2.105856334 |
| YIR028W | *dal4Δ* |  |  | 2.10355162 |  |  | 2.10355162 |
| YGL046W | *ygl046wΔ* |  |  | 2.102245895 |  |  | 2.102245895 |
| YPL223C | *gre1Δ* |  |  |  | 2.099527585 |  | 2.099527585 |
| YDR273W | *don1Δ* |  |  |  | 2.098907179 |  | 2.098907179 |
| YKL093W | *mbr1Δ* |  |  | 2.095276451 |  | 2.011063508 | 2.095276451 |
| YDR271C | *ydr271cΔ* |  |  |  |  | 2.094777218 | 2.094777218 |
| YBR126C | *tps1Δ* |  | 2.092052569 |  |  |  | 2.092052569 |
| YJR040W | *gef1Δ* |  |  | 2.090415904 |  |  | 2.090415904 |
| YNL099C | *oca1Δ* |  |  | 2.086598095 |  |  | 2.086598095 |
| YHR010W | *rpl27aΔ* |  |  |  |  | 2.086104488 | 2.086104488 |
| YPL204W | *hrr25-ts* |  |  | 2.084272942 |  |  | 2.084272942 |
| YGR035W-A | *ygr035w-aΔ* |  |  | 2.082639702 |  |  | 2.082639702 |
| YJL056C | *zap1Δ* |  |  |  |  | 2.08022091 | 2.08022091 |
| YDL011C | *ydl011cΔ* |  |  | 2.079761076 |  |  | 2.079761076 |
| YLR382C | *nam2Δ* |  | 2.07799729 |  |  |  | 2.07799729 |
| YGL026C | *trp5Δ* |  |  | 2.075963395 |  |  | 2.075963395 |
| YKL218C | *sry1Δ* |  |  | 2.07519679 |  |  | 2.07519679 |
| YBR145W | *adh5Δ* |  |  | 2.072295422 |  |  | 2.072295422 |
| YKL200C | *ykl200cΔ* |  |  | 2.06895179 |  |  | 2.06895179 |
| YGR254W | *eno1Δ* |  |  |  |  | 2.068140815 | 2.068140815 |
| YLR119W | *srn2Δ* |  |  |  | 2.066298043 |  | 2.066298043 |
| YNL176C | *ynl176cΔ* |  |  | 2.065304361 |  |  | 2.065304361 |
| YOL122C | *smf1Δ* |  |  |  |  | 2.062653331 | 2.062653331 |
| YGL108C | *ygl108cΔ* |  |  |  |  | 2.062343278 | 2.062343278 |
| YJL189W | *rpl39Δ* |  |  | 2.058822181 |  |  | 2.058822181 |
| YIL112W | *hos4Δ* |  | 2.054492546 |  |  |  | 2.054492546 |
| YER038W-A | *yer038w-aΔ* |  |  | 2.053157791 |  |  | 2.053157791 |
| YDR363W | *esc2Δ* |  |  |  |  | 2.050850635 | 2.050850635 |
| YPL158C | *aim44Δ* |  |  | 2.05055417 |  |  | 2.05055417 |
| YDR246W | *trs23-ts* |  |  |  |  | 2.047125989 | 2.047125989 |
| YGR042W | *ygr042wΔ* |  |  | 2.04650701 |  |  | 2.04650701 |
| YNL279W | *prm1Δ* |  |  | 2.046160096 |  |  | 2.046160096 |
| YDR272W | *glo2Δ* |  |  |  |  | 2.037133802 | 2.037133802 |
| YIL133C | *rpl16aΔ* |  |  | 2.033652858 |  |  | 2.033652858 |
| YEL071W | *dld3Δ* |  |  | 2.033286763 |  |  | 2.033286763 |
| YER176W | *ecm32Δ* |  |  | 2.031992973 |  |  | 2.031992973 |
| YDR260C | *swm1Δ* |  |  | 2.031671307 |  |  | 2.031671307 |
| YLR251W | *sym1Δ* |  |  | 2.028660476 |  | 2.016649526 | 2.028660476 |
| YBL051C | *pin4Δ* |  |  | 2.022076038 |  | 2.000612867 | 2.022076038 |
| YDR245W | *mnn10Δ* |  |  |  | 2.017427714 |  | 2.017427714 |
| YIL059C | *yil059cΔ* |  |  | 2.015372823 |  |  | 2.015372823 |
| YPL055C | *lge1Δ* |  |  |  |  | 2.012110649 | 2.012110649 |
| YDR009W | *gal3Δ* |  |  | 2.006305768 |  |  | 2.006305768 |
| YGL028C | *scw11Δ* |  |  |  |  | 2.003134887 | 2.003134887 |
| YHR189W | *pth1Δ* |  |  | 2.000193232 |  |  | 2.000193232 |
