# Supplementary material for: A Quantitative, High-Throughput Reverse Genetic Screen Reveals Novel Connections between Pre–mRNA Splicing and 5′ and 3′ End Transcript Determinants
Source: PLoS Genet. 2012 Mar 29;8(3):e1002530. doi: 10.1371/journal.pgen.1002530 (PMC3315463; doi:10.1371/journal.pgen.1002530)
Supplement: Table S3 — The complete list of the strains used in this study. The systematic names of all genes which were either deleted or contained a temperature sensitive mutation are noted, as well as their mutations and a reference to their sources. The sources are a = Giaver et al., 2002; b = Ben-Aroya et al., 2008; c = from the Guthrie lab collection. (DOCX) [file pgen.1002530.s012.docx]

| **Systematic name** | **Mutation** | **Source** |
| --- | --- | --- |
| YAL002W | *vps8Δ* | a |
| YAL004W | *yal004wΔ* | a |
| YAL005C | *ssa1Δ* | a |
| YAL007C | *erp2Δ* | a |
| YAL008W | *fun14Δ* | a |
| YAL009W | *spo7Δ* | a |
| YAL010C | *mdm10Δ* | a |
| YAL011W | *swc3Δ* | a |
| YAL012W | *cys3Δ* | a |
| YAL013W | *dep1Δ* | a |
| YAL014C | *syn8Δ* | a |
| YAL015C | *ntg1Δ* | a |
| YAL016C-B | *yal016c-bΔ* | a |
| YAL016W | *tpd3Δ* | a |
| YAL017W | *psk1Δ* | a |
| YAL018C | *yal018cΔ* | a |
| YAL019W | *fun30Δ* | a |
| YAL020C | *ats1Δ* | a |
| YAL021C | *ccr4Δ* | a |
| YAL021C | *ccr4Δ* | a |
| YAL022C | *fun26Δ* | a |
| YAL023C | *pmt2Δ* | a |
| YAL024C | *lte1Δ* | a |
| YAL025C | *mak16-ts* | b |
| YAL026C | *drs2Δ* | a |
| YAL027W | *saw1Δ* | a |
| YAL028W | *frt2Δ* | a |
| YAL029C | *myo4Δ* | a |
| YAL030W | *snc1Δ* | a |
| YAL031C | *gip4Δ* | a |
| YAL034C | *fun19Δ* | a |
| YAL035W | *fun12Δ* | a |
| YAL036C | *rbg1Δ* | a |
| YAL037C-A | *yal037c-aΔ* | a |
| YAL037W | *yal037wΔ* | a |
| YAL039C | *cyc3Δ* | a |
| YAL040C | *cln3Δ* | a |
| YAL042W | *erv46Δ* | a |
| YAL043C | *pta1-ts* | b |
| YAL043C-a | *yal043c-aΔ* | a |
| YAL044C | *gcv3Δ* | a |
| YAL044W-A | *yal044w-aΔ* | a |
| YAL045C | *yal045cΔ* | a |
| YAL046C | *aim1Δ* | a |
| YAL047C | *spc72Δ* | a |
| YAL048C | *gem1Δ* | a |
| YAL049C | *aim2Δ* | a |
| YAL049C | *aim2Δ* | a |
| YAL051W | *oaf1Δ* | a |
| YAL053W | *flc2Δ* | a |
| YAL054C | *acs1Δ* | a |
| YAL055W | *pex22Δ* | a |
| YAL056W | *gpb2Δ* | a |
| YAL058C-A | *yal058c-aΔ* | a |
| YAL058W | *cne1Δ* | a |
| YAL059W | *ecm1Δ* | a |
| YAL060W | *bdh1Δ* | a |
| YAL061W | *bdh2Δ* | a |
| YAL062W | *gdh3Δ* | a |
| YAL064C-A | *yal064c-aΔ* | a |
| YAL065C | *yal065cΔ* | a |
| YAL066W | *yal066wΔ* | a |
| YAL067C | *seo1Δ* | a |
| YAL067W-A | *yal067w-aΔ* | a |
| YAL068C | *yal068cΔ* | a |
| YAR002C-A | *erp1Δ* | a |
| YAR002W | *nup60Δ* | a |
| YAR003W | *swd1Δ* | a |
| YAR008W | *sen34-ts* | b |
| YAR014C | *bud14Δ* | a |
| YAR015W | *ade1Δ* | a |
| YAR018C | *kin3Δ* | a |
| YAR020C | *pau7Δ* | a |
| YAR023C | *yar023cΔ* | a |
| YAR027W | *uip3Δ* | a |
| YAR028W | *yar028wΔ* | a |
| YAR029W | *yar029wΔ* | a |
| YAR030C | *yar030cΔ* | a |
| YAR031W | *prm9Δ* | a |
| YAR035C-A | *yar035c-aΔ* | a |
| YAR035W | *yat1Δ* | a |
| YAR037W | *yar037wΔ* | a |
| YAR040C | *yar040cΔ* | a |
| YAR042W | *swh1Δ* | a |
| YAR042W | *swh1Δ* | a |
| YAR043C | *yar043cΔ* | a |
| YAR044W | *yar044wΔ* | a |
| YAR047C | *yar047cΔ* | a |
| YAR050W | *flo1Δ* | a |
| YBL001C | *ecm15Δ* | a |
| YBL002W | *htb2Δ* | a |
| YBL003C | *hta2Δ* | a |
| YBL005W | *pdr3Δ* | a |
| YBL006C | *ldb7Δ* | a |
| YBL007C | *sla1Δ* | a |
| YBL008W | *hir1Δ* | a |
| YBL008W-A | *ybl008w-aΔ* | a |
| YBL009W | *alk2Δ* | a |
| YBL010C | *ybl010cΔ* | a |
| YBL011W | *sct1Δ* | a |
| YBL012C | *ybl012cΔ* | a |
| YBL013W | *fmt1Δ* | a |
| YBL015W | *ach1Δ* | a |
| YBL016W | *fus3Δ* | a |
| YBL017C | *pep1Δ* | a |
| YBL019W | *apn2Δ* | a |
| YBL021C | *hap3Δ* | a |
| YBL022C | *pim1Δ* | a |
| YBL024W | *ncl1Δ* | a |
| YBL025W | *rrn10Δ* | a |
| YBL026W | *lsm2-ts* | b |
| YBL027W | *rpl19bΔ* | a |
| YBL028C | *ybl028cΔ* | a |
| YBL029C-A | *ybl029c-aΔ* | a |
| YBL029W | *ybl029wΔ* | a |
| YBL031W | *she1Δ* | a |
| YBL032W | *hek2Δ* | a |
| YBL033C | *rib1Δ* | a |
| YBL036C | *ybl036cΔ* | a |
| YBL037W | *apl3Δ* | a |
| YBL038W | *mrpl16Δ* | a |
| YBL039C | *ura7Δ* | a |
| YBL039W-A | *ybl039w-aΔ* | a |
| YBL040C | *erd2-ts* | b |
| YBL041W | *pre7-ts* | b |
| YBL042C | *fui1Δ* | a |
| YBL043W | *ecm13Δ* | a |
| YBL044W | *ybl044wΔ* | a |
| YBL045C | *cor1Δ* | a |
| YBL046W | *psy4Δ* | a |
| YBL047C | *ede1Δ* | a |
| YBL048W | *rrt1Δ* | a |
| YBL049W | *moh1Δ* | a |
| YBL051C | *pin4Δ* | a |
| YBL052C | *sas3Δ* | a |
| YBL053W | *ybl053wΔ* | a |
| YBL054W | *tod6Δ* | a |
| YBL055C | *ybl055cΔ* | a |
| YBL056W | *ptc3Δ* | a |
| YBL057C | *pth2Δ* | a |
| YBL058W | *shp1Δ* | a |
| YBL059W | *ybl059wΔ* | a |
| YBL060W | *yel1Δ* | a |
| YBL061C | *skt5Δ* | a |
| YBL062W | *ybl062wΔ* | a |
| YBL063W | *kip1Δ* | a |
| YBL064C | *prx1Δ* | a |
| YBL065W | *ybl065wΔ* | a |
| YBL066C | *sef1Δ* | a |
| YBL067C | *ubp13Δ* | a |
| YBL068W | *prs4Δ* | a |
| YBL069W | *ast1Δ* | a |
| YBL070C | *ybl070cΔ* | a |
| YBL071C | *ybl071cΔ* | a |
| YBL071C-B | *ybl071c-bΔ* | a |
| YBL071W-A | *kti11Δ* | a |
| YBL072C | *rps8aΔ* | a |
| YBL074C | *aar2-ts* | b |
| YBL075C | *ssa3Δ* | a |
| YBL078C | *atg8Δ* | a |
| YBL079W | *nup170Δ* | a |
| YBL080C | *pet112Δ* | a |
| YBL081W | *ybl081wΔ* | a |
| YBL082C | *alg3Δ* | a |
| YBL083C | *ybl083cΔ* | a |
| YBL085W | *boi1Δ* | a |
| YBL086C | *ybl086cΔ* | a |
| YBL087C | *rpl23aΔ* | a |
| YBL088C | *tel1Δ* | a |
| YBL089W | *avt5Δ* | a |
| YBL090W | *mrp21Δ* | a |
| YBL091C | *map2Δ* | a |
| YBL091C-A | *scs22Δ* | a |
| YBL091C-A | *scs22Δ* | a |
| YBL093C | *rox3Δ* | a |
| YBL094C | *ybl094cΔ* | a |
| YBL095W | *ybl095wΔ* | a |
| YBL096C | *ybl096cΔ* | a |
| YBL098W | *bna4Δ* | a |
| YBL099W | *atp1Δ* | a |
| YBL100C | *ybl100cΔ* | a |
| YBL101C | *ecm21Δ* | a |
| YBL101W-C | *ybl101w-cΔ* | a |
| YBL102W | *sft2Δ* | a |
| YBL103C | *rtg3Δ* | a |
| YBL104C | *ybl104cΔ* | a |
| YBL104C | *ybl104cΔ* | a |
| YBL106C | *sro77Δ* | a |
| YBL107C | *ybl107cΔ* | a |
| YBR001C | *nth2Δ* | a |
| YBR003W | *coq1Δ* | a |
| YBR004C | *gpi18-ts* | b |
| YBR005W | *rcr1Δ* | a |
| YBR006W | *uga2Δ* | a |
| YBR007C | *dsf2Δ* | a |
| YBR008C | *flr1Δ* | a |
| YBR009C | *hhf1Δ* | a |
| YBR010W | *hht1Δ* | a |
| YBR011C | *ipp1-ts* | b |
| YBR012C | *ybr012cΔ* | a |
| YBR013C | *ybr013cΔ* | a |
| YBR014C | *grx7Δ* | a |
| YBR015C | *mnn2Δ* | a |
| YBR016W | *ybr016wΔ* | a |
| YBR018C | *gal7Δ* | a |
| YBR019C | *gal10Δ* | a |
| YBR020W | *gal1Δ* | a |
| YBR020W | *gal1Δ* | a |
| YBR021W | *fur4Δ* | a |
| YBR022W | *poa1Δ* | a |
| YBR023C | *chs3Δ* | a |
| YBR024W | *sco2Δ* | a |
| YBR025C | *ola1Δ* | a |
| YBR026C | *etr1Δ* | a |
| YBR027C | *ybr027cΔ* | a |
| YBR028C | *ybr028cΔ* | a |
| YBR029C | *cds1-ts* | b |
| YBR030W | *rkm3Δ* | a |
| YBR031W | *rpl4aΔ* | a |
| YBR032W | *ybr032wΔ* | a |
| YBR033W | *eds1Δ* | a |
| YBR034C | *hmt1Δ* | a |
| YBR035C | *pdx3Δ* | a |
| YBR036C | *csg2Δ* | a |
| YBR037C | *sco1Δ* | a |
| YBR040W | *fig1Δ* | a |
| YBR041W | *fat1Δ* | a |
| YBR042C | *cst26Δ* | a |
| YBR043C | *qdr3Δ* | a |
| YBR044C | *tcm62Δ* | a |
| YBR045C | *gip1Δ* | a |
| YBR046C | *zta1Δ* | a |
| YBR047W | *fmp23Δ* | a |
| YBR048W | *rps11bΔ* | a |
| YBR049C | *reb1-ts* | b |
| YBR050C | *reg2Δ* | a |
| YBR051W | *ybr051wΔ* | a |
| YBR052C | *rfs1Δ* | a |
| YBR053C | *ybr053cΔ* | a |
| YBR054W | *yro2Δ* | a |
| YBR055C | *prp6-1* | c |
| YBR056W | *ybr056wΔ* | a |
| YBR056W-A | *ybr056w-aΔ* | a |
| YBR057C | *mum2Δ* | a |
| YBR058C | *ubp14Δ* | a |
| YBR058C-A | *tsc3Δ* | a |
| YBR059C | *akl1Δ* | a |
| YBR061C | *trm7Δ* | a |
| YBR062C | *ybr062cΔ* | a |
| YBR062C | *ybr062cΔ* | a |
| YBR063C | *ybr063cΔ* | a |
| YBR064W | *ybr064wΔ* | a |
| YBR065C | *ecm2Δ* | a |
| YBR066C | *nrg2Δ* | a |
| YBR067C | *tip1Δ* | a |
| YBR068C | *bap2Δ* | a |
| YBR069C | *tat1Δ* | a |
| YBR070C | *alg14-ts* | b |
| YBR071W | *ybr071wΔ* | a |
| YBR072C-A | *ybr072c-aΔ* | a |
| YBR072W | *hsp26Δ* | a |
| YBR073W | *rdh54Δ* | a |
| YBR074W | *ybr074wΔ* | a |
| YBR074W | *ybr074wΔ* | a |
| YBR075W | *ybr075wΔ* | a |
| YBR075W | *ybr075wΔ* | a |
| YBR076W | *ecm8Δ* | a |
| YBR077C | *slm4Δ* | a |
| YBR078W | *ecm33Δ* | a |
| YBR079C | *rpg1-ts* | b |
| YBR081C | *spt7Δ* | a |
| YBR082C | *ubc4Δ* | a |
| YBR083W | *tec1Δ* | a |
| YBR084C-A | *rpl19aΔ* | a |
| YBR084W | *mis1Δ* | a |
| YBR085C-A | *ybr085c-aΔ* | a |
| YBR085W | *aac3Δ* | a |
| YBR088C | *pol30-ts* | b |
| YBR090C | *ybr090cΔ* | a |
| YBR090C-A | *ybr090c-aΔ* | a |
| YBR092C | *pho3Δ* | a |
| YBR093C | *pho5Δ* | a |
| YBR094W | *pby1Δ* | a |
| YBR095C | *rxt2Δ* | a |
| YBR097W | *vps15Δ* | a |
| YBR098W | *mms4Δ* | a |
| YBR098W | *mms4Δ* | a |
| YBR099C | *ybr099cΔ* | a |
| YBR100W | *ybr100wΔ* | a |
| YBR100W | *ybr100wΔ* | a |
| YBR101C | *fes1Δ* | a |
| YBR103W | *sif2Δ* | a |
| YBR104W | *ymc2Δ* | a |
| YBR105C | *vid24Δ* | a |
| YBR105C | *vid24Δ* | a |
| YBR106W | *pho88Δ* | a |
| YBR107C | *iml3Δ* | a |
| YBR108W | *aim3Δ* | a |
| YBR111C | *ysa1Δ* | a |
| YBR111W-A | *sus1Δ* | a |
| YBR112C | *cyc8Δ* | a |
| YBR113W | *ybr113wΔ* | a |
| YBR114W | *rad16Δ* | a |
| YBR115C | *lys2Δ* | a |
| YBR116C | *ybr116cΔ* | a |
| YBR119W | *mud1Δ* | a |
| YBR120C | *cbp6Δ* | a |
| YBR121C | *grs1Δ* | a |
| YBR121C | *grs1-ts* | b |
| YBR122C | *mrpl36Δ* | a |
| YBR122C | *mrpl36Δ* | a |
| YBR125C | *ptc4Δ* | a |
| YBR126C | *tps1Δ* | a |
| YBR127C | *vma2Δ* | a |
| YBR128C | *atg14Δ* | a |
| YBR129C | *opy1Δ* | a |
| YBR130C | *she3Δ* | a |
| YBR131W | *ccz1Δ* | a |
| YBR132C | *agp2Δ* | a |
| YBR133C | *hsl7Δ* | a |
| YBR133C | *hsl7Δ* | a |
| YBR134W | *ybr134wΔ* | a |
| YBR137W | *ybr137wΔ* | a |
| YBR138C | *ybr138cΔ* | a |
| YBR139W | *ybr139wΔ* | a |
| YBR141C | *ybr141cΔ* | a |
| YBR142W | *mak5-ts* | b |
| YBR144C | *ybr144cΔ* | a |
| YBR145W | *adh5Δ* | a |
| YBR146W | *mrps9Δ* | a |
| YBR147W | *rtc2Δ* | a |
| YBR148W | *ysw1Δ* | a |
| YBR149W | *ara1Δ* | a |
| YBR150C | *tbs1Δ* | a |
| YBR151W | *apd1Δ* | a |
| YBR152W | *spp381-ts* | b |
| YBR153W | *rib7-ts* | b |
| YBR154C | *rpb5-ts* | b |
| YBR156C | *sli15Δ* | a |
| YBR157C | *ics2Δ* | a |
| YBR157C | *ics2Δ* | a |
| YBR158W | *amn1Δ* | a |
| YBR159W | *ifa38Δ* | a |
| YBR161W | *csh1Δ* | a |
| YBR162C | *tos1Δ* | a |
| YBR162W-A | *ysy6Δ* | a |
| YBR163W | *dem1Δ* | a |
| YBR164C | *arl1Δ* | a |
| YBR165W | *ubs1Δ* | a |
| YBR166C | *tyr1Δ* | a |
| YBR167C | *pop7-ts* | b |
| YBR168W | *pex32Δ* | a |
| YBR169C | *sse2Δ* | a |
| YBR170C | *npl4Δ* | a |
| YBR171W | *sec66Δ* | a |
| YBR172C | *smy2Δ* | a |
| YBR173C | *ump1Δ* | a |
| YBR174C | *ybr174cΔ* | a |
| YBR175W | *swd3Δ* | a |
| YBR176W | *ecm31Δ* | a |
| YBR177C | *eht1Δ* | a |
| YBR178W | *ybr178wΔ* | a |
| YBR179C | *fzo1Δ* | a |
| YBR180W | *dtr1Δ* | a |
| YBR181C | *rps6bΔ* | a |
| YBR182C | *smp1Δ* | a |
| YBR182C-A | *ybr182c-aΔ* | a |
| YBR183W | *ypc1Δ* | a |
| YBR184W | *ybr184wΔ* | a |
| YBR185C | *mba1Δ* | a |
| YBR186W | *pch2Δ* | a |
| YBR187W | *gdt1Δ* | a |
| YBR188C | *ntc20Δ* | a |
| YBR189W | *rps9bΔ* | a |
| YBR191W | *rpl21aΔ* | a |
| YBR194W | *aim4Δ* | a |
| YBR195C | *msi1Δ* | a |
| YBR196C-A | *ybr196c-aΔ* | a |
| YBR196C-B | *ybr196c-bΔ* | a |
| YBR197C | *ybr197cΔ* | a |
| YBR199W | *ktr4Δ* | a |
| YBR200W | *bem1Δ* | a |
| YBR200W-A | *ybr200w-aΔ* | a |
| YBR201W | *der1Δ* | a |
| YBR201W | *der1Δ* | a |
| YBR203W | *cos111Δ* | a |
| YBR204C | *ybr204cΔ* | a |
| YBR205W | *ktr3Δ* | a |
| YBR206W | *ybr206wΔ* | a |
| YBR207W | *fth1Δ* | a |
| YBR208C | *dur1,2Δ* | a |
| YBR209W | *ybr209wΔ* | a |
| YBR210W | *erv15Δ* | a |
| YBR212W | *ngr1Δ* | a |
| YBR213W | *met8Δ* | a |
| YBR214W | *sds24Δ* | a |
| YBR215W | *hpc2Δ* | a |
| YBR216C | *ybp1Δ* | a |
| YBR217W | *atg12Δ* | a |
| YBR218C | *pyc2Δ* | a |
| YBR219C | *ybr219cΔ* | a |
| YBR220C | *ybr220cΔ* | a |
| YBR221C | *pdb1Δ* | a |
| YBR221W-A | *ybr221w-aΔ* | a |
| YBR222C | *pcs60Δ* | a |
| YBR223C | *tdp1Δ* | a |
| YBR224W | *ybr224wΔ* | a |
| YBR225W | *ybr225wΔ* | a |
| YBR226C | *ybr226cΔ* | a |
| YBR227C | *mcx1Δ* | a |
| YBR228W | *slx1Δ* | a |
| YBR229C | *rot2Δ* | a |
| YBR230C | *om14Δ* | a |
| YBR230W-A | *ybr230w-aΔ* | a |
| YBR231C | *swc5Δ* | a |
| YBR232C | *ybr232cΔ* | a |
| YBR233W | *pbp2Δ* | a |
| YBR234C | *arc40-ts* | b |
| YBR235W | *ybr235wΔ* | a |
| YBR237W | *prp5-1* | c |
| YBR238C | *ybr238cΔ* | a |
| YBR239C | *ybr239cΔ* | a |
| YBR240C | *thi2Δ* | a |
| YBR241C | *ybr241cΔ* | a |
| YBR242W | *ybr242wΔ* | a |
| YBR244W | *gpx2Δ* | a |
| YBR245C | *isw1Δ* | a |
| YBR246W | *rrt2Δ* | a |
| YBR248C | *his7Δ* | a |
| YBR249C | *aro4Δ* | a |
| YBR250W | *spo23Δ* | a |
| YBR251W | *mrps5Δ* | a |
| YBR253W | *srb6-ts* | b |
| YBR254C | *trs20-ts* | b |
| YBR255W | *mtc4Δ* | a |
| YBR256C | *rib5-ts* | b |
| YBR257W | *pop4-ts* | b |
| YBR258C | *shg1Δ* | a |
| YBR259W | *ybr259wΔ* | a |
| YBR260C | *rgd1Δ* | a |
| YBR261C | *tae1Δ* | a |
| YBR262C | *aim5Δ* | a |
| YBR263W | *shm1Δ* | a |
| YBR264C | *ypt10Δ* | a |
| YBR266C | *slm6Δ* | a |
| YBR267W | *rei1Δ* | a |
| YBR268W | *mrpl37Δ* | a |
| YBR269C | *fmp21Δ* | a |
| YBR270C | *bit2Δ* | a |
| YBR271W | *ybr271wΔ* | a |
| YBR272C | *hsm3Δ* | a |
| YBR273C | *ubx7Δ* | a |
| YBR274W | *chk1Δ* | a |
| YBR274W | *chk1Δ* | a |
| YBR275C | *rif1Δ* | a |
| YBR276C | *pps1Δ* | a |
| YBR277C | *ybr277cΔ* | a |
| YBR278W | *dpb3Δ* | a |
| YBR279W | *paf1Δ* | a |
| YBR280C | *saf1Δ* | a |
| YBR281C | *dug2Δ* | a |
| YBR282W | *mrpl27Δ* | a |
| YBR283C | *ssh1Δ* | a |
| YBR284W | *ybr284wΔ* | a |
| YBR285W | *ybr285wΔ* | a |
| YBR286W | *ape3Δ* | a |
| YBR287W | *ybr287wΔ* | a |
| YBR288C | *apm3Δ* | a |
| YBR289W | *snf5Δ* | a |
| YBR290W | *bsd2Δ* | a |
| YBR291C | *ctp1Δ* | a |
| YBR292C | *ybr292cΔ* | a |
| YBR293W | *vba2Δ* | a |
| YBR294W | *sul1Δ* | a |
| YBR295W | *pca1Δ* | a |
| YBR296C | *pho89Δ* | a |
| YBR296C-A | *ybr296c-aΔ* | a |
| YBR297W | *mal33Δ* | a |
| YBR298C | *mal31Δ* | a |
| YBR299W | *mal32Δ* | a |
| YBR300C | *ybr300cΔ* | a |
| YBR301W | *dan3Δ* | a |
| YCL001W | *rer1Δ* | a |
| YCL001W-A | *ycl001w-aΔ* | a |
| YCL001W-B | *ycl001w-bΔ* | a |
| YCL002C | *ycl002cΔ* | a |
| YCL002C | *ycl002cΔ* | a |
| YCL004W | *pgs1-ts* | b |
| YCL005W | *ldb16Δ* | a |
| YCL005W-A | *vma9Δ* | a |
| YCL006C | *ycl006cΔ* | a |
| YCL006C | *ycl006cΔ* | a |
| YCL007C | *ycl007cΔ* | a |
| YCL008C | *stp22Δ* | a |
| YCL009C | *ilv6Δ* | a |
| YCL010C | *sgf29Δ* | a |
| YCL011C | *gbp2Δ* | a |
| YCL012C | *ycl012cΔ* | a |
| YCL012W | *ycl012wΔ* | a |
| YCL013W | *ycl013wΔ* | a |
| YCL014W | *bud3Δ* | a |
| YCL014W | *bud3Δ* | a |
| YCL016C | *dcc1Δ* | a |
| YCL021W-A | *ycl021w-aΔ* | a |
| YCL022C | *ycl022cΔ* | a |
| YCL023C | *ycl023cΔ* | a |
| YCL023C | *ycl023cΔ* | a |
| YCL024W | *kcc4Δ* | a |
| YCL025C | *agp1Δ* | a |
| YCL026C | *ycl026cΔ* | a |
| YCL026C-A | *frm2Δ* | a |
| YCL026C-B | *hbn1Δ* | a |
| YCL027W | *fus1Δ* | a |
| YCL028W | *rnq1Δ* | a |
| YCL029C | *bik1Δ* | a |
| YCL030C | *his4Δ* | a |
| YCL032W | *ste50Δ* | a |
| YCL033C | *mxr2Δ* | a |
| YCL034W | *lsb5Δ* | a |
| YCL035C | *grx1Δ* | a |
| YCL036W | *gfd2Δ* | a |
| YCL037C | *sro9Δ* | a |
| YCL038C | *atg22Δ* | a |
| YCL038C | *atg22Δ* | a |
| YCL039W | *gid7Δ* | a |
| YCL040W | *glk1Δ* | a |
| YCL042W | *ycl042wΔ* | a |
| YCL044C | *mgr1Δ* | a |
| YCL045C | *emc1Δ* | a |
| YCL046W | *ycl046wΔ* | a |
| YCL047C | *ycl047cΔ* | a |
| YCL048W | *sps22Δ* | a |
| YCL049C | *ycl049cΔ* | a |
| YCL050C | *apa1Δ* | a |
| YCL051W | *lre1Δ* | a |
| YCL052C | *pbn1-ts* | b |
| YCL055W | *kar4Δ* | a |
| YCL056C | *ycl056cΔ* | a |
| YCL057C-A | *ycl057c-aΔ* | a |
| YCL057W | *prd1Δ* | a |
| YCL058C | *fyv5Δ* | a |
| YCL060C | *ycl060cΔ* | a |
| YCL061C | *mrc1Δ* | a |
| YCL061C | *mrc1Δ* | a |
| YCL062W | *ycl062wΔ* | a |
| YCL063W | *vac17Δ* | a |
| YCL064C | *cha1Δ* | a |
| YCL069W | *vba3Δ* | a |
| YCL074W | *ycl074wΔ* | a |
| YCL075W | *ycl075wΔ* | a |
| YCL076W | *ycl076wΔ* | a |
| YCR001W | *ycr001wΔ* | a |
| YCR002C | *cdc10Δ* | a |
| YCR003W | *mrpl32Δ* | a |
| YCR004C | *ycp4Δ* | a |
| YCR005C | *cit2Δ* | a |
| YCR006C | *ycr006cΔ* | a |
| YCR007C | *ycr007cΔ* | a |
| YCR008W | *sat4Δ* | a |
| YCR009C | *rvs161Δ* | a |
| YCR010C | *ady2Δ* | a |
| YCR011C | *adp1Δ* | a |
| YCR014C | *pol4Δ* | a |
| YCR015C | *ycr015cΔ* | a |
| YCR016W | *ycr016wΔ* | a |
| YCR017C | *cwh43Δ* | a |
| YCR019W | *mak32Δ* | a |
| YCR020C | *pet18Δ* | a |
| YCR020C-A | *mak31Δ* | a |
| YCR020W-B | *htl1Δ* | a |
| YCR021C | *hsp30Δ* | a |
| YCR022C | *ycr022cΔ* | a |
| YCR023C | *ycr023cΔ* | a |
| YCR024C | *slm5Δ* | a |
| YCR024C-A | *pmp1Δ* | a |
| YCR025C | *ycr025cΔ* | a |
| YCR026C | *npp1Δ* | a |
| YCR027C | *rhb1Δ* | a |
| YCR028C | *fen2Δ* | a |
| YCR028C-A | *rim1Δ* | a |
| YCR030C | *syp1Δ* | a |
| YCR031C | *rps14aΔ* | a |
| YCR032W | *bph1Δ* | a |
| YCR033W | *snt1Δ* | a |
| YCR034W | *fen1Δ* | a |
| YCR036W | *rbk1Δ* | a |
| YCR037C | *pho87Δ* | a |
| YCR043C | *ycr043cΔ* | a |
| YCR044C | *per1Δ* | a |
| YCR045C | *rrt12Δ* | a |
| YCR046C | *img1Δ* | a |
| YCR047C | *bud23Δ* | a |
| YCR048W | *are1Δ* | a |
| YCR049C | *ycr049cΔ* | a |
| YCR050C | *ycr050cΔ* | a |
| YCR051W | *ycr051wΔ* | a |
| YCR052W | *rsc6-ts* | b |
| YCR053W | *thr4Δ* | a |
| YCR057C | *pwp2-ts* | b |
| YCR059C | *yih1Δ* | a |
| YCR060W | *tah1Δ* | a |
| YCR061W | *ycr061wΔ* | a |
| YCR061W | *ycr061wΔ* | a |
| YCR061W | *ycr061wΔ* | a |
| YCR062W | *ycr062wΔ* | a |
| YCR063W | *bud31Δ* | a |
| YCR065W | *hcm1Δ* | a |
| YCR066W | *rad18Δ* | a |
| YCR067C | *sed4Δ* | a |
| YCR068W | *atg15Δ* | a |
| YCR069W | *cpr4Δ* | a |
| YCR071C | *img2Δ* | a |
| YCR072C | *rsa4-ts* | b |
| YCR073C | *ssk22Δ* | a |
| YCR073W-A | *sol2Δ* | a |
| YCR075C | *ers1Δ* | a |
| YCR075W-A | *ycr075w-aΔ* | a |
| YCR076C | *ycr076cΔ* | a |
| YCR077C | *pat1Δ* | a |
| YCR079W | *ptc6Δ* | a |
| YCR081W | *srb8Δ* | a |
| YCR082W | *ahc2Δ* | a |
| YCR083W | *trx3Δ* | a |
| YCR084C | *tup1Δ* | a |
| YCR085W | *ycr085wΔ* | a |
| YCR086W | *csm1Δ* | a |
| YCR087C-A | *ycr087c-aΔ* | a |
| YCR087W | *ycr087wΔ* | a |
| YCR088W | *abp1Δ* | a |
| YCR089W | *fig2Δ* | a |
| YCR090C | *ycr090cΔ* | a |
| YCR091W | *kin82Δ* | a |
| YCR092C | *msh3Δ* | a |
| YCR094W | *cdc50Δ* | a |
| YCR095C | *oca4Δ* | a |
| YCR095W-A | *ycr095w-aΔ* | a |
| YCR098C | *git1Δ* | a |
| YCR099C | *ycr099cΔ* | a |
| YCR100C | *ycr100cΔ* | a |
| YCR101C | *ycr101cΔ* | a |
| YCR102C | *ycr102cΔ* | a |
| YCR102W-A | *ycr102w-aΔ* | a |
| YCR105W | *adh7Δ* | a |
| YCR106W | *rds1Δ* | a |
| YCR107W | *aad3Δ* | a |
| YCRO54C | *ycro54c-ts* | b |
| YDL001W | *rmd1Δ* | a |
| YDL002C | *nhp10Δ* | a |
| YDL003W | *mcd1-ts* | b |
| YDL006W | *ptc1Δ* | a |
| YDL009C | *ydl009cΔ* | a |
| YDL010W | *grx6Δ* | a |
| YDL011C | *ydl011cΔ* | a |
| YDL012C | *ydl012cΔ* | a |
| YDL013W | *slx5Δ* | a |
| YDL015C | *tsc13-ts* | b |
| YDL018C | *erp3Δ* | a |
| YDL019C | *osh2Δ* | a |
| YDL020C | *rpn4Δ* | a |
| YDL021W | *gpm2Δ* | a |
| YDL022W | *gpd1Δ* | a |
| YDL023C | *ydl023cΔ* | a |
| YDL024C | *dia3Δ* | a |
| YDL025C | *ydl025cΔ* | a |
| YDL026W | *ydl026wΔ* | a |
| YDL026W | *ydl026wΔ* | a |
| YDL027C | *ydl027cΔ* | a |
| YDL030W | *prp9-1* | c |
| YDL032W | *ydl032wΔ* | a |
| YDL033C | *slm3Δ* | a |
| YDL034W | *ydl034wΔ* | a |
| YDL035C | *gpr1Δ* | a |
| YDL036C | *pus9Δ* | a |
| YDL036C | *pus9Δ* | a |
| YDL037C | *bsc1Δ* | a |
| YDL038C | *ydl038cΔ* | a |
| YDL039C | *prm7Δ* | a |
| YDL040C | *nat1Δ* | a |
| YDL041W | *ydl041wΔ* | a |
| YDL042C | *sir2Δ* | a |
| YDL043C | *prp11-1* | c |
| YDL044C | *mtf2Δ* | a |
| YDL045C | *fad1-ts* | b |
| YDL045W-A | *mrp10Δ* | a |
| YDL046W | *npc2Δ* | a |
| YDL047W | *sit4Δ* | a |
| YDL048C | *stp4Δ* | a |
| YDL049C | *knh1Δ* | a |
| YDL050C | *ydl050cΔ* | a |
| YDL051W | *lhp1Δ* | a |
| YDL052C | *slc1Δ* | a |
| YDL053C | *pbp4Δ* | a |
| YDL054C | *mch1Δ* | a |
| YDL056W | *mbp1Δ* | a |
| YDL057W | *ydl057wΔ* | a |
| YDL059C | *rad59Δ* | a |
| YDL061C | *rps29bΔ* | a |
| YDL062W | *ydl062wΔ* | a |
| YDL063C | *ydl063cΔ* | a |
| YDL065C | *pex19Δ* | a |
| YDL066W | *idp1Δ* | a |
| YDL067C | *cox9Δ* | a |
| YDL068W | *ydl068wΔ* | a |
| YDL069C | *cbs1Δ* | a |
| YDL069C | *cbs1Δ* | a |
| YDL070W | *bdf2Δ* | a |
| YDL071C | *ydl071cΔ* | a |
| YDL072C | *yet3Δ* | a |
| YDL073W | *ydl073wΔ* | a |
| YDL074C | *bre1Δ* | a |
| YDL075W | *rpl31aΔ* | a |
| YDL076C | *rxt3Δ* | a |
| YDL077C | *vam6Δ* | a |
| YDL077C | *vam6Δ* | a |
| YDL078C | *mdh3Δ* | a |
| YDL079C | *mrk1Δ* | a |
| YDL080C | *thi3Δ* | a |
| YDL081C | *rpp1aΔ* | a |
| YDL082W | *rpl13aΔ* | a |
| YDL083C | *rps16bΔ* | a |
| YDL084W | *sub2-T113V* | c |
| YDL085C-A | *ydl085c-aΔ* | a |
| YDL085W | *nde2Δ* | a |
| YDL086W | *ydl086wΔ* | a |
| YDL088C | *asm4Δ* | a |
| YDL089W | *nur1Δ* | a |
| YDL090C | *ram1Δ* | a |
| YDL091C | *ubx3Δ* | a |
| YDL093W | *pmt5Δ* | a |
| YDL094C | *ydl094cΔ* | a |
| YDL095W | *pmt1Δ* | a |
| YDL096C | *opi6Δ* | a |
| YDL097C | *rpn6-ts* | b |
| YDL098C | *snu23-ts* | b |
| YDL099W | *bug1Δ* | a |
| YDL100C | *get3Δ* | a |
| YDL101C | *dun1Δ* | a |
| YDL103C | *qri1-ts* | b |
| YDL104C | *qri7Δ* | a |
| YDL105W | *nse4-ts* | b |
| YDL106C | *pho2Δ* | a |
| YDL107W | *mss2Δ* | a |
| YDL109C | *ydl109cΔ* | a |
| YDL110C | *tma17Δ* | a |
| YDL111C | *rrp42-ts* | b |
| YDL112W | *trm3Δ* | a |
| YDL113C | *atg20Δ* | a |
| YDL114W | *ydl114wΔ* | a |
| YDL115C | *iwr1Δ* | a |
| YDL116W | *nup84Δ* | a |
| YDL117W | *cyk3Δ* | a |
| YDL118W | *ydl118wΔ* | a |
| YDL119C | *ydl119cΔ* | a |
| YDL121C | *ydl121cΔ* | a |
| YDL122W | *ubp1Δ* | a |
| YDL123W | *sna4Δ* | a |
| YDL124W | *ydl124wΔ* | a |
| YDL125C | *hnt1Δ* | a |
| YDL127W | *pcl2Δ* | a |
| YDL128W | *vcx1Δ* | a |
| YDL129W | *ydl129wΔ* | a |
| YDL130W | *rpp1bΔ* | a |
| YDL130W-A | *stf1Δ* | a |
| YDL131W | *lys21Δ* | a |
| YDL132W | *cdc53-ts* | b |
| YDL133C-A | *rpl41bΔ* | a |
| YDL133W | *ydl133wΔ* | a |
| YDL134C | *pph21Δ* | a |
| YDL134C-A | *ydl134c-aΔ* | a |
| YDL135C | *rdi1Δ* | a |
| YDL136W | *rpl35bΔ* | a |
| YDL137W | *arf2Δ* | a |
| YDL138W | *rgt2Δ* | a |
| YDL141W | *bpl1-ts* | b |
| YDL142C | *crd1Δ* | a |
| YDL144C | *ydl144cΔ* | a |
| YDL146W | *ldb17Δ* | a |
| YDL147W | *rpn5-ts* | b |
| YDL148C | *nop14-ts* | b |
| YDL149W | *atg9Δ* | a |
| YDL151C | *bud30Δ* | a |
| YDL154W | *msh5Δ* | a |
| YDL155W | *clb3Δ* | a |
| YDL156W | *ydl156wΔ* | a |
| YDL157C | *ydl157cΔ* | a |
| YDL159W | *ste7Δ* | a |
| YDL159W-A | *ydl159w-aΔ* | a |
| YDL160C | *dhh1Δ* | a |
| YDL160C-A | *ydl160c-aΔ* | a |
| YDL161W | *ent1Δ* | a |
| YDL162C | *ydl162cΔ* | a |
| YDL166C | *fap7-ts* | b |
| YDL167C | *nrp1Δ* | a |
| YDL168W | *sfa1Δ* | a |
| YDL169C | *ugx2Δ* | a |
| YDL170W | *uga3Δ* | a |
| YDL171C | *glt1Δ* | a |
| YDL172C | *ydl172cΔ* | a |
| YDL173W | *ydl173wΔ* | a |
| YDL174C | *dld1Δ* | a |
| YDL175C | *air2Δ* | a |
| YDL176W | *ydl176wΔ* | a |
| YDL177C | *ydl177cΔ* | a |
| YDL178W | *dld2Δ* | a |
| YDL179W | *pcl9Δ* | a |
| YDL180W | *ydl180wΔ* | a |
| YDL181W | *inh1Δ* | a |
| YDL182W | *lys20Δ* | a |
| YDL183C | *ydl183cΔ* | a |
| YDL184C | *rpl41aΔ* | a |
| YDL185W | *tfp1Δ* | a |
| YDL186W | *ydl186wΔ* | a |
| YDL187C | *ydl187cΔ* | a |
| YDL188C | *pph22Δ* | a |
| YDL189W | *rbs1Δ* | a |
| YDL190C | *ufd2Δ* | a |
| YDL191W | *rpl35aΔ* | a |
| YDL192W | *arf1Δ* | a |
| YDL193W | *nus1-ts* | b |
| YDL194W | *snf3Δ* | a |
| YDL197C | *asf2Δ* | a |
| YDL198C | *ggc1Δ* | a |
| YDL199C | *ydl199cΔ* | a |
| YDL200C | *mgt1Δ* | a |
| YDL201W | *trm8Δ* | a |
| YDL202W | *mrpl11Δ* | a |
| YDL203C | *ack1Δ* | a |
| YDL204W | *rtn2Δ* | a |
| YDL206W | *ydl206wΔ* | a |
| YDL208W | *nhp2-ts* | b |
| YDL209C | *cwc2-ts* | b |
| YDL210W | *uga4Δ* | a |
| YDL211C | *ydl211cΔ* | a |
| YDL213C | *nop6Δ* | a |
| YDL214C | *prr2Δ* | a |
| YDL215C | *gdh2Δ* | a |
| YDL216C | *rri1Δ* | a |
| YDL218W | *ydl218wΔ* | a |
| YDL219W | *dtd1Δ* | a |
| YDL222C | *fmp45Δ* | a |
| YDL223C | *hbt1Δ* | a |
| YDL224C | *whi4Δ* | a |
| YDL225W | *shs1Δ* | a |
| YDL226C | *gcs1Δ* | a |
| YDL227C | *hoΔ* | a |
| YDL229W | *ssb1Δ* | a |
| YDL230W | *ptp1Δ* | a |
| YDL231C | *bre4Δ* | a |
| YDL232W | *ost4Δ* | a |
| YDL233W | *ydl233wΔ* | a |
| YDL234C | *gyp7Δ* | a |
| YDL236W | *pho13Δ* | a |
| YDL237W | *aim6Δ* | a |
| YDL238C | *gud1Δ* | a |
| YDL239C | *ady3Δ* | a |
| YDL240W | *lrg1Δ* | a |
| YDL241W | *ydl241wΔ* | a |
| YDL242W | *ydl242wΔ* | a |
| YDL243C | *aad4Δ* | a |
| YDR001C | *nth1Δ* | a |
| YDR003W | *rcr2Δ* | a |
| YDR003W-A | *ydr003w-aΔ* | a |
| YDR004W | *rad57Δ* | a |
| YDR005C | *maf1Δ* | a |
| YDR006C | *sok1Δ* | a |
| YDR007W | *trp1Δ* | a |
| YDR008C | *ydr008cΔ* | a |
| YDR009W | *gal3Δ* | a |
| YDR010C | *ydr010cΔ* | a |
| YDR011W | *snq2Δ* | a |
| YDR014W | *rad61Δ* | a |
| YDR015C | *ydr015cΔ* | a |
| YDR016C | *dad1-ts* | b |
| YDR017C | *kcs1Δ* | a |
| YDR018C | *ydr018cΔ* | a |
| YDR019C | *gcv1Δ* | a |
| YDR020C | *das2Δ* | a |
| YDR022C | *cis1Δ* | a |
| YDR024W | *fyv1Δ* | a |
| YDR025W | *rps11aΔ* | a |
| YDR026C | *ydr026cΔ* | a |
| YDR027C | *vps54Δ* | a |
| YDR028C | *reg1Δ* | a |
| YDR029W | *ydr029wΔ* | a |
| YDR030C | *rad28Δ* | a |
| YDR031W | *mic14Δ* | a |
| YDR032C | *pst2Δ* | a |
| YDR033W | *mrh1Δ* | a |
| YDR034C | *lys14Δ* | a |
| YDR034W-B | *ydr034w-bΔ* | a |
| YDR035W | *aro3Δ* | a |
| YDR036C | *ehd3Δ* | a |
| YDR042C | *ydr042cΔ* | a |
| YDR043C | *nrg1Δ* | a |
| YDR044W | *hem13-ts* | b |
| YDR045C | *rpc11-ts* | b |
| YDR046C | *bap3Δ* | a |
| YDR048C | *ydr048cΔ* | a |
| YDR049W | *ydr049wΔ* | a |
| YDR050C | *tpi1Δ* | a |
| YDR051C | *det1Δ* | a |
| YDR055W | *pst1Δ* | a |
| YDR056C | *ydr056cΔ* | a |
| YDR057W | *yos9Δ* | a |
| YDR058C | *tgl2Δ* | a |
| YDR059C | *ubc5Δ* | a |
| YDR061W | *ydr061wΔ* | a |
| YDR063W | *aim7Δ* | a |
| YDR064W | *rps13-ts* | b |
| YDR065W | *ydr065wΔ* | a |
| YDR066C | *rtr2Δ* | a |
| YDR067C | *oca6Δ* | a |
| YDR068W | *dos2Δ* | a |
| YDR069C | *doa4Δ* | a |
| YDR070C | *fmp16Δ* | a |
| YDR071C | *paa1Δ* | a |
| YDR072C | *ipt1Δ* | a |
| YDR073W | *snf11Δ* | a |
| YDR074W | *tps2Δ* | a |
| YDR075W | *pph3Δ* | a |
| YDR076W | *rad55Δ* | a |
| YDR077W | *sed1Δ* | a |
| YDR078C | *shu2Δ* | a |
| YDR079C-A | *tfb5Δ* | a |
| YDR079W | *pet100Δ* | a |
| YDR080W | *vps41Δ* | a |
| YDR081C | *pdc2-ts* | b |
| YDR083W | *rrp8Δ* | a |
| YDR084C | *tvp23Δ* | a |
| YDR085C | *afr1Δ* | a |
| YDR086C | *sss1-ts* | b |
| YDR089W | *ydr089wΔ* | a |
| YDR090C | *ydr090cΔ* | a |
| YDR090C | *ydr090cΔ* | a |
| YDR091C | *rli1-ts* | b |
| YDR092W | *ubc13Δ* | a |
| YDR092W | *ubc13Δ* | a |
| YDR093W | *dnf2Δ* | a |
| YDR094W | *ydr094wΔ* | a |
| YDR095C | *ydr095cΔ* | a |
| YDR096W | *gis1Δ* | a |
| YDR097C | *msh6Δ* | a |
| YDR098C | *grx3Δ* | a |
| YDR099W | *bmh2Δ* | a |
| YDR100W | *tvp15Δ* | a |
| YDR101C | *arx1Δ* | a |
| YDR102C | *ydr102cΔ* | a |
| YDR103W | *ste5Δ* | a |
| YDR104C | *spo71Δ* | a |
| YDR105C | *tms1Δ* | a |
| YDR107C | *tmn2Δ* | a |
| YDR108W | *gsg1Δ* | a |
| YDR109C | *ydr109cΔ* | a |
| YDR110W | *fob1Δ* | a |
| YDR111C | *alt2Δ* | a |
| YDR112W | *irc2Δ* | a |
| YDR114C | *ydr114cΔ* | a |
| YDR115W | *ydr115wΔ* | a |
| YDR116C | *mrpl1Δ* | a |
| YDR117C | *tma64Δ* | a |
| YDR118W | *apc4-ts* | b |
| YDR119W | *vba4Δ* | a |
| YDR120C | *trm1Δ* | a |
| YDR121W | *dpb4Δ* | a |
| YDR122W | *kin1Δ* | a |
| YDR123C | *ino2Δ* | a |
| YDR124W | *ydr124wΔ* | a |
| YDR125C | *ecm18Δ* | a |
| YDR126W | *swf1Δ* | a |
| YDR127W | *aro1Δ* | a |
| YDR128W | *mtc5Δ* | a |
| YDR129C | *sac6Δ* | a |
| YDR130C | *fin1Δ* | a |
| YDR131C | *ydr131cΔ* | a |
| YDR132C | *ydr132cΔ* | a |
| YDR133C | *ydr133cΔ* | a |
| YDR134C | *ydr134cΔ* | a |
| YDR135C | *ycf1Δ* | a |
| YDR136C | *vps61Δ* | a |
| YDR137W | *rgp1Δ* | a |
| YDR138W | *hpr1Δ* | a |
| YDR139C | *rub1Δ* | a |
| YDR140W | *mtq2Δ* | a |
| YDR142C | *pex7Δ* | a |
| YDR143C | *san1Δ* | a |
| YDR144C | *mkc7Δ* | a |
| YDR146C | *swi5Δ* | a |
| YDR147W | *eki1Δ* | a |
| YDR147W | *eki1Δ* | a |
| YDR148C | *kgd2Δ* | a |
| YDR149C | *ydr149cΔ* | a |
| YDR150W | *num1Δ* | a |
| YDR151C | *cth1Δ* | a |
| YDR152W | *gir2Δ* | a |
| YDR153C | *ent5Δ* | a |
| YDR154C | *ydr154cΔ* | a |
| YDR155C | *cpr1Δ* | a |
| YDR156W | *rpa14Δ* | a |
| YDR157W | *ydr157wΔ* | a |
| YDR158W | *hom2Δ* | a |
| YDR159W | *sac3Δ* | a |
| YDR161W | *ydr161wΔ* | a |
| YDR162C | *nbp2Δ* | a |
| YDR163W | *cwc15Δ* | a |
| YDR165W | *trm82Δ* | a |
| YDR169C | *stb3Δ* | a |
| YDR169C-A | *ydr169c-aΔ* | a |
| YDR171W | *hsp42Δ* | a |
| YDR173C | *arg82Δ* | a |
| YDR174W | *hmo1Δ* | a |
| YDR175C | *rsm24Δ* | a |
| YDR176W | *ngg1Δ* | a |
| YDR178W | *sdh4Δ* | a |
| YDR179C | *csn9Δ* | a |
| YDR179W-A | *ydr179w-aΔ* | a |
| YDR179W-A | *ydr179w-aΔ* | a |
| YDR180w | *ydr180w-ts* | b |
| YDR181C | *sas4Δ* | a |
| YDR182W-A | *ydr182w-aΔ* | a |
| YDR183W | *plp1Δ* | a |
| YDR184C | *atc1Δ* | a |
| YDR185C | *ydr185cΔ* | a |
| YDR186C | *ydr186cΔ* | a |
| YDR191W | *hst4Δ* | a |
| YDR192C | *nup42Δ* | a |
| YDR193W | *ydr193wΔ* | a |
| YDR194C | *mss116Δ* | a |
| YDR194W-A | *ydr194w-aΔ* | a |
| YDR195W | *ref2Δ* | a |
| YDR196C | *cab5-ts* | b |
| YDR197W | *cbs2Δ* | a |
| YDR198C | *rkm2Δ* | a |
| YDR199W | *ydr199wΔ* | a |
| YDR200C | *vps64Δ* | a |
| YDR201W | *spc19-ts* | b |
| YDR202C | *rav2Δ* | a |
| YDR203W | *ydr203wΔ* | a |
| YDR204W | *coq4Δ* | a |
| YDR205W | *msc2Δ* | a |
| YDR206W | *ebs1Δ* | a |
| YDR207C | *ume6Δ* | a |
| YDR209C | *ydr209cΔ* | a |
| YDR210W | *ydr210wΔ* | a |
| YDR213W | *upc2Δ* | a |
| YDR214W | *aha1Δ* | a |
| YDR215C | *ydr215cΔ* | a |
| YDR216W | *adr1Δ* | a |
| YDR217C | *rad9Δ* | a |
| YDR218C | *spr28Δ* | a |
| YDR219C | *mfb1Δ* | a |
| YDR220C | *ydr220cΔ* | a |
| YDR221W | *gtb1Δ* | a |
| YDR222W | *ydr222wΔ* | a |
| YDR223W | *crf1Δ* | a |
| YDR225W | *hta1Δ* | a |
| YDR226W | *adk1Δ* | a |
| YDR227W | *sir4Δ* | a |
| YDR229W | *ivy1Δ* | a |
| YDR230W | *ydr230wΔ* | a |
| YDR231C | *cox20Δ* | a |
| YDR232W | *hem1-ts* | b |
| YDR233C | *rtn1Δ* | a |
| YDR234W | *lys4Δ* | a |
| YDR235W | *prp42-ts* | b |
| YDR236C | *fmn1-ts* | b |
| YDR237W | *mrpl7Δ* | a |
| YDR239C | *ydr239cΔ* | a |
| YDR240C | *snu56-ts* | b |
| YDR241W | *bud26Δ* | a |
| YDR242W | *amd2Δ* | a |
| YDR243C | *prp28-1* | c |
| YDR244W | *pex5Δ* | a |
| YDR245W | *mnn10Δ* | a |
| YDR246W | *trs23-ts* | b |
| YDR246W-A | *ydr246w-aΔ* | a |
| YDR247W | *vhs1Δ* | a |
| YDR248C | *ydr248cΔ* | a |
| YDR249C | *ydr249cΔ* | a |
| YDR250C | *ydr250cΔ* | a |
| YDR251W | *pam1Δ* | a |
| YDR252W | *btt1Δ* | a |
| YDR253C | *met32Δ* | a |
| YDR254W | *chl4Δ* | a |
| YDR255C | *rmd5Δ* | a |
| YDR256C | *cta1Δ* | a |
| YDR257C | *rkm4Δ* | a |
| YDR258C | *hsp78Δ* | a |
| YDR259C | *yap6Δ* | a |
| YDR260C | *swm1Δ* | a |
| YDR261C | *exg2Δ* | a |
| YDR262W | *ydr262wΔ* | a |
| YDR263C | *din7Δ* | a |
| YDR264C | *akr1Δ* | a |
| YDR265W | *pex10Δ* | a |
| YDR266C | *ydr266cΔ* | a |
| YDR267C | *cia1-ts* | b |
| YDR268W | *msw1Δ* | a |
| YDR269C | *ydr269cΔ* | a |
| YDR270W | *ccc2Δ* | a |
| YDR271C | *ydr271cΔ* | a |
| YDR272W | *glo2Δ* | a |
| YDR273W | *don1Δ* | a |
| YDR274C | *ydr274cΔ* | a |
| YDR275W | *bsc2Δ* | a |
| YDR276C | *pmp3Δ* | a |
| YDR277C | *mth1Δ* | a |
| YDR278C | *ydr278cΔ* | a |
| YDR279W | *rnh202Δ* | a |
| YDR280W | *rrp45-ts* | b |
| YDR281C | *phm6Δ* | a |
| YDR282C | *ydr282cΔ* | a |
| YDR283C | *gcn2Δ* | a |
| YDR284C | *dpp1Δ* | a |
| YDR285W | *zip1Δ* | a |
| YDR286C | *ydr286cΔ* | a |
| YDR287W | *inm2Δ* | a |
| YDR288W | *nse3-ts* | b |
| YDR289C | *rtt103Δ* | a |
| YDR290W | *ydr290wΔ* | a |
| YDR291W | *hrq1Δ* | a |
| YDR293C | *ssd1Δ* | a |
| YDR294C | *dpl1Δ* | a |
| YDR295C | *hda2Δ* | a |
| YDR296W | *mhr1Δ* | a |
| YDR297W | *sur2Δ* | a |
| YDR298C | *atp5Δ* | a |
| YDR299W | *bfr2-ts* | b |
| YDR300C | *pro1Δ* | a |
| YDR302W | *gpi11-ts* | b |
| YDR304C | *cpr5Δ* | a |
| YDR305C | *hnt2Δ* | a |
| YDR306C | *ydr306cΔ* | a |
| YDR307W | *ydr307wΔ* | a |
| YDR309C | *gic2Δ* | a |
| YDR310C | *sum1Δ* | a |
| YDR312W | *ssf2Δ* | a |
| YDR313C | *pib1Δ* | a |
| YDR314C | *rad34Δ* | a |
| YDR315C | *ipk1Δ* | a |
| YDR315C | *ipk1Δ* | a |
| YDR316W | *oms1Δ* | a |
| YDR317W | *him1Δ* | a |
| YDR318W | *mcm21Δ* | a |
| YDR318W | *mcm21Δ* | a |
| YDR319C | *ydr319cΔ* | a |
| YDR320C | *swa2Δ* | a |
| YDR321W | *asp1Δ* | a |
| YDR322C-A | *tim11Δ* | a |
| YDR322W | *mrpl35Δ* | a |
| YDR323C | *pep7Δ* | a |
| YDR326C | *ysp2Δ* | a |
| YDR329C | *pex3Δ* | a |
| YDR330W | *ubx5Δ* | a |
| YDR331W | *gpi8-ts* | b |
| YDR332W | *irc3Δ* | a |
| YDR333C | *ydr333cΔ* | a |
| YDR334W | *swr1Δ* | a |
| YDR335W | *msn5Δ* | a |
| YDR336W | *ydr336wΔ* | a |
| YDR337W | *mrps28Δ* | a |
| YDR338C | *ydr338cΔ* | a |
| YDR339C | *fcf1-ts* | b |
| YDR340W | *ydr340wΔ* | a |
| YDR341C | *ydr341c-ts* | b |
| YDR344C | *ydr344cΔ* | a |
| YDR345C | *hxt3Δ* | a |
| YDR346C | *svf1Δ* | a |
| YDR347W | *mrp1Δ* | a |
| YDR348C | *ydr348cΔ* | a |
| YDR349C | *yps7Δ* | a |
| YDR350C | *atp22Δ* | a |
| YDR351W | *sbe2Δ* | a |
| YDR352W | *ydr352wΔ* | a |
| YDR354W | *trp4Δ* | a |
| YDR357C | *ydr357cΔ* | a |
| YDR358W | *gga1Δ* | a |
| YDR359C | *eaf1Δ* | a |
| YDR360W | *opi7Δ* | a |
| YDR363W | *esc2Δ* | a |
| YDR363W-A | *sem1Δ* | a |
| YDR364C | *prp17-1* | c |
| YDR364C | *cdc40Δ* | a |
| YDR367W | *ydr367w-ts* | b |
| YDR368W | *ypr1Δ* | a |
| YDR369C | *xrs2Δ* | a |
| YDR370C | *ydr370cΔ* | a |
| YDR371W | *cts2Δ* | a |
| YDR372C | *vps74Δ* | a |
| YDR374C | *ydr374cΔ* | a |
| YDR375C | *bcs1Δ* | a |
| YDR377W | *atp17Δ* | a |
| YDR378C | *lsm6Δ* | a |
| YDR379C-A | *ydr379c-aΔ* | a |
| YDR379W | *rga2Δ* | a |
| YDR380W | *aro10Δ* | a |
| YDR382W | *rpp2bΔ* | a |
| YDR383C | *nkp1Δ* | a |
| YDR384C | *ato3Δ* | a |
| YDR385W | *eft2Δ* | a |
| YDR386W | *mus81Δ* | a |
| YDR387C | *ydr387cΔ* | a |
| YDR388W | *rvs167Δ* | a |
| YDR389W | *sac7Δ* | a |
| YDR391C | *ydr391cΔ* | a |
| YDR392W | *spt3Δ* | a |
| YDR393W | *she9Δ* | a |
| YDR395W | *sxm1Δ* | a |
| YDR398W | *utp5-ts* | b |
| YDR399W | *hpt1Δ* | a |
| YDR400W | *urh1Δ* | a |
| YDR401W | *ydr401wΔ* | a |
| YDR402C | *dit2Δ* | a |
| YDR403W | *dit1Δ* | a |
| YDR404C | *rpb7-ts* | b |
| YDR405W | *mrp20Δ* | a |
| YDR406W | *pdr15Δ* | a |
| YDR408C | *ade8Δ* | a |
| YDR409W | *siz1Δ* | a |
| YDR410C | *ste14Δ* | a |
| YDR411C | *dfm1Δ* | a |
| YDR414C | *erd1Δ* | a |
| YDR415C | *ydr415cΔ* | a |
| YDR416W | *syf1-ts* | b |
| YDR417C | *ydr417cΔ* | a |
| YDR417C | *ydr417cΔ* | a |
| YDR418W | *rpl12bΔ* | a |
| YDR419W | *rad30Δ* | a |
| YDR420W | *hkr1Δ* | a |
| YDR421W | *aro80Δ* | a |
| YDR422C | *sip1Δ* | a |
| YDR423C | *cad1Δ* | a |
| YDR424C | *dyn2Δ* | a |
| YDR425W | *snx41Δ* | a |
| YDR426C | *ydr426cΔ* | a |
| YDR428C | *bna7Δ* | a |
| YDR430C | *cym1Δ* | a |
| YDR431W | *ydr431wΔ* | a |
| YDR432W | *npl3-S411A* | c |
| YDR432W | *npl3Δ* | a |
| YDR433W | *ydr433wΔ* | a |
| YDR433W | *ydr433wΔ* | a |
| YDR434W | *gpi17-ts* | b |
| YDR435C | *ppm1Δ* | a |
| YDR436W | *ppz2Δ* | a |
| YDR437W | *gpi19-ts* | b |
| YDR438W | *thi74Δ* | a |
| YDR439W | *lrs4Δ* | a |
| YDR440W | *dot1Δ* | a |
| YDR441C | *apt2Δ* | a |
| YDR442W | *ydr442wΔ* | a |
| YDR443C | *ssn2Δ* | a |
| YDR444W | *ydr444wΔ* | a |
| YDR445C | *ydr445cΔ* | a |
| YDR446W | *ecm11Δ* | a |
| YDR447C | *rps17bΔ* | a |
| YDR448W | *ada2Δ* | a |
| YDR448W | *ada2Δ* | a |
| YDR449C | *utp6-ts* | b |
| YDR450W | *rps18aΔ* | a |
| YDR451C | *yhp1Δ* | a |
| YDR452W | *ppn1Δ* | a |
| YDR453C | *tsa2Δ* | a |
| YDR454C | *guk1-ts* | b |
| YDR455C | *ydr455cΔ* | a |
| YDR456W | *nhx1Δ* | a |
| YDR457W | *tom1Δ* | a |
| YDR458C | *heh2Δ* | a |
| YDR459C | *pfa5Δ* | a |
| YDR461W | *mfa1Δ* | a |
| YDR462W | *mrpl28Δ* | a |
| YDR463W | *stp1Δ* | a |
| YDR465C | *rmt2Δ* | a |
| YDR466W | *pkh3Δ* | a |
| YDR467C | *ydr467cΔ* | a |
| YDR469W | *sdc1Δ* | a |
| YDR470C | *ugo1Δ* | a |
| YDR471W | *rpl27bΔ* | a |
| YDR472W | *trs31-ts* | b |
| YDR473C | *prp3-1* | c |
| YDR474C | *ydr474cΔ* | a |
| YDR475C | *jip4Δ* | a |
| YDR475C | *jip4Δ* | a |
| YDR476C | *ydr476cΔ* | a |
| YDR477W | *snf1Δ* | a |
| YDR478W | *snm1-ts* | b |
| YDR479C | *pex29Δ* | a |
| YDR480W | *dig2Δ* | a |
| YDR481C | *pho8Δ* | a |
| YDR482C | *cwc21Δ* | a |
| YDR483W | *kre2Δ* | a |
| YDR484W | *vps52Δ* | a |
| YDR485C | *vps72Δ* | a |
| YDR485C | *vps72Δ* | a |
| YDR486C | *vps60Δ* | a |
| YDR487C | *rib3-ts* | b |
| YDR488C | *pac11Δ* | a |
| YDR489W | *sld5-ts* | b |
| YDR490C | *pkh1Δ* | a |
| YDR491C | *ydr491cΔ* | a |
| YDR492W | *izh1Δ* | a |
| YDR493W | *aim8Δ* | a |
| YDR494W | *rsm28Δ* | a |
| YDR495C | *vps3Δ* | a |
| YDR496C | *puf6Δ* | a |
| YDR497C | *itr1Δ* | a |
| YDR500C | *rpl37bΔ* | a |
| YDR500C | *rpl37bΔ* | a |
| YDR501W | *plm2Δ* | a |
| YDR501W | *plm2Δ* | a |
| YDR502C | *sam2Δ* | a |
| YDR503C | *lpp1Δ* | a |
| YDR504C | *spg3Δ* | a |
| YDR505C | *psp1Δ* | a |
| YDR506C | *ydr506cΔ* | a |
| YDR506C | *ydr506cΔ* | a |
| YDR507C | *gin4Δ* | a |
| YDR508C | *gnp1Δ* | a |
| YDR509W | *ydr509wΔ* | a |
| YDR511W | *acn9Δ* | a |
| YDR512C | *emi1Δ* | a |
| YDR512C | *emi1Δ* | a |
| YDR513W | *grx2Δ* | a |
| YDR514C | *ydr514cΔ* | a |
| YDR515W | *slf1Δ* | a |
| YDR516C | *emi2Δ* | a |
| YDR517W | *grh1Δ* | a |
| YDR518W | *eug1Δ* | a |
| YDR518W | *eug1Δ* | a |
| YDR519W | *fpr2Δ* | a |
| YDR520C | *urc2Δ* | a |
| YDR521W | *ydr521wΔ* | a |
| YDR522C | *sps2Δ* | a |
| YDR523C | *sps1Δ* | a |
| YDR524C | *age1Δ* | a |
| YDR524C-B | *ydr524c-bΔ* | a |
| YDR524W-A | *ydr524w-aΔ* | a |
| YDR525W | *api2Δ* | a |
| YDR525W-A | *sna2Δ* | a |
| YDR527W | *rba50-ts* | b |
| YDR528W | *hlr1Δ* | a |
| YDR529C | *qcr7Δ* | a |
| YDR530C | *apa2Δ* | a |
| YDR531W | *cab1-ts* | b |
| YDR532C | *ydr532cΔ* | a |
| YDR533C | *hsp31Δ* | a |
| YDR534C | *fit1Δ* | a |
| YDR535C | *ydr535cΔ* | a |
| YDR536W | *stl1Δ* | a |
| YDR537C | *ydr537cΔ* | a |
| YDR538W | *pad1Δ* | a |
| YDR539W | *ydr539wΔ* | a |
| YDR540C | *irc4Δ* | a |
| YDR541C | *ydr541cΔ* | a |
| YEL001C | *irc22Δ* | a |
| YEL003W | *gim4Δ* | a |
| YEL004W | *yea4Δ* | a |
| YEL005C | *vab2Δ* | a |
| YEL006W | *yea6Δ* | a |
| YEL007W | *yel007wΔ* | a |
| YEL008W | *yel008wΔ* | a |
| YEL009C | *gcn4Δ* | a |
| YEL010W | *yel010wΔ* | a |
| YEL011W | *glc3Δ* | a |
| YEL012W | *ubc8Δ* | a |
| YEL013W | *vac8Δ* | a |
| YEL014C | *yel014cΔ* | a |
| YEL015W | *edc3Δ* | a |
| YEL016C | *npp2Δ* | a |
| YEL017C-A | *pmp2Δ* | a |
| YEL017W | *gtt3Δ* | a |
| YEL020C | *yel020cΔ* | a |
| YEL022W | *gea2Δ* | a |
| YEL023C | *yel023cΔ* | a |
| YEL024W | *rip1Δ* | a |
| YEL025C | *yel025cΔ* | a |
| YEL026W | *snu13-ts* | b |
| YEL027W | *cup5Δ* | a |
| YEL028W | *yel028wΔ* | a |
| YEL029C | *bud16Δ* | a |
| YEL030W | *ecm10Δ* | a |
| YEL031W | *spf1Δ* | a |
| YEL033W | *mtc7Δ* | a |
| YEL036C | *anp1Δ* | a |
| YEL037C | *rad23Δ* | a |
| YEL038W | *utr4Δ* | a |
| YEL039C | *cyc7Δ* | a |
| YEL040W | *utr2Δ* | a |
| YEL041W | *yef1Δ* | a |
| YEL041W | *yef1Δ* | a |
| YEL042W | *gda1Δ* | a |
| YEL043W | *yel043wΔ* | a |
| YEL044W | *ies6Δ* | a |
| YEL045C | *yel045cΔ* | a |
| YEL046C | *gly1Δ* | a |
| YEL047C | *yel047cΔ* | a |
| YEL048C | *yel048cΔ* | a |
| YEL049W | *pau2Δ* | a |
| YEL050C | *rml2Δ* | a |
| YEL051W | *vma8Δ* | a |
| YEL052W | *afg1Δ* | a |
| YEL053C | *mak10Δ* | a |
| YEL054C | *rpl12aΔ* | a |
| YEL056W | *hat2Δ* | a |
| YEL057C | *yel057cΔ* | a |
| YEL059C-A | *som1Δ* | a |
| YEL059W | *yel059wΔ* | a |
| YEL060C | *prb1Δ* | a |
| YEL061C | *cin8Δ* | a |
| YEL062W | *npr2Δ* | a |
| YEL063C | *can1Δ* | a |
| YEL064C | *avt2Δ* | a |
| YEL065W | *sit1Δ* | a |
| YEL066W | *hpa3Δ* | a |
| YEL067C | *yel067cΔ* | a |
| YEL068C | *yel068cΔ* | a |
| YEL071W | *dld3Δ* | a |
| YEL072W | *rmd6Δ* | a |
| YER001W | *mnn1Δ* | a |
| YER002W | *nop16Δ* | a |
| YER003C | *pmi40-ts* | b |
| YER004W | *fmp52Δ* | a |
| YER005W | *ynd1Δ* | a |
| YER007C-A | *tma20Δ* | a |
| YER007W | *pac2Δ* | a |
| YER007W | *pac2Δ* | a |
| YER009W | *ntf2-ts* | b |
| YER010C | *yer010cΔ* | a |
| YER011W | *tir1Δ* | a |
| YER012W | *pre1-ts* | b |
| YER013W | *prp22-1* | c |
| YER014C-A | *bud25Δ* | a |
| YER014W | *hem14Δ* | a |
| YER015W | *faa2Δ* | a |
| YER016W | *bim1Δ* | a |
| YER016W | *bim1Δ* | a |
| YER017C | *afg3Δ* | a |
| YER019C-A | *sbh2Δ* | a |
| YER019W | *isc1Δ* | a |
| YER020W | *gpa2Δ* | a |
| YER023W | *pro3-ts* | b |
| YER024W | *yat2Δ* | a |
| YER026C | *cho1Δ* | a |
| YER027C | *gal83Δ* | a |
| YER028C | *mig3Δ* | a |
| YER030W | *chz1Δ* | a |
| YER031C | *ypt31Δ* | a |
| YER032W | *fir1Δ* | a |
| YER033C | *zrg8Δ* | a |
| YER034W | *yer034wΔ* | a |
| YER035W | *edc2Δ* | a |
| YER037W | *phm8Δ* | a |
| YER038W-A | *yer038w-aΔ* | a |
| YER039C | *hvg1Δ* | a |
| YER039C-A | *yer039c-aΔ* | a |
| YER040W | *gln3Δ* | a |
| YER041W | *yen1Δ* | a |
| YER042W | *mxr1Δ* | a |
| YER044C | *erg28Δ* | a |
| YER044C-A | *mei4Δ* | a |
| YER045C | *aca1Δ* | a |
| YER046W | *spo73Δ* | a |
| YER046W-A | *yer046w-aΔ* | a |
| YER047C | *sap1Δ* | a |
| YER048C | *caj1Δ* | a |
| YER049W | *tpa1Δ* | a |
| YER050C | *rsm18Δ* | a |
| YER051W | *jhd1Δ* | a |
| YER052C | *hom3Δ* | a |
| YER053C | *pic2Δ* | a |
| YER053C-A | *yer053c-aΔ* | a |
| YER054C | *gip2Δ* | a |
| YER055C | *his1Δ* | a |
| YER056C | *fcy2Δ* | a |
| YER056C-A | *rpl34aΔ* | a |
| YER057C | *hmf1Δ* | a |
| YER058W | *pet117Δ* | a |
| YER059W | *pcl6Δ* | a |
| YER060W | *fcy21Δ* | a |
| YER060W-A | *fcy22Δ* | a |
| YER061C | *cem1Δ* | a |
| YER062C | *hor2Δ* | a |
| YER063W | *tho1Δ* | a |
| YER064C | *yer064cΔ* | a |
| YER065C | *icl1Δ* | a |
| YER066C-A | *yer066c-aΔ* | a |
| YER066W | *rrt13Δ* | a |
| YER067C-A | *yer067c-aΔ* | a |
| YER067W | *yer067wΔ* | a |
| YER068C-A | *yer068c-aΔ* | a |
| YER068W | *mot2Δ* | a |
| YER069W | *arg5,6Δ* | a |
| YER070W | *rnr1Δ* | a |
| YER071C | *yer071cΔ* | a |
| YER072W | *vtc1Δ* | a |
| YER073W | *ald5Δ* | a |
| YER074W | *rps24aΔ* | a |
| YER075C | *ptp3Δ* | a |
| YER076C | *yer076cΔ* | a |
| YER077C | *yer077cΔ* | a |
| YER078C | *yer078cΔ* | a |
| YER079W | *yer079wΔ* | a |
| YER080W | *aim9Δ* | a |
| YER081W | *ser3Δ* | a |
| YER083C | *get2Δ* | a |
| YER084W | *yer084wΔ* | a |
| YER085C | *yer085cΔ* | a |
| YER086W | *ilv1Δ* | a |
| YER087C-A | *yer087c-aΔ* | a |
| YER087C-B | *sbh1Δ* | a |
| YER087W | *aim10Δ* | a |
| YER088C | *dot6Δ* | a |
| YER089C | *ptc2Δ* | a |
| YER090W | *trp2Δ* | a |
| YER091C | *met6Δ* | a |
| YER091C-A | *yer091c-aΔ* | a |
| YER092W | *ies5Δ* | a |
| YER093C-A | *yer093c-aΔ* | a |
| YER094C | *pup3-ts* | b |
| YER095W | *rad51Δ* | a |
| YER096W | *shc1Δ* | a |
| YER097W | *yer097wΔ* | a |
| YER098W | *ubp9Δ* | a |
| YER099C | *prs2Δ* | a |
| YER101C | *ast2Δ* | a |
| YER103W | *ssa4Δ* | a |
| YER105C | *nup157Δ* | a |
| YER106W | *mam1Δ* | a |
| YER108C | *yer108cΔ* | a |
| YER109C | *flo8Δ* | a |
| YER109C | *flo8Δ* | a |
| YER110C | *kap123Δ* | a |
| YER111C | *swi4Δ* | a |
| YER113C | *tmn3Δ* | a |
| YER114C | *boi2Δ* | a |
| YER115C | *spr6Δ* | a |
| YER116C | *slx8Δ* | a |
| YER117W | *rpl23bΔ* | a |
| YER118C | *sho1Δ* | a |
| YER119C | *avt6Δ* | a |
| YER119C-A | *yer119c-aΔ* | a |
| YER120W | *scs2Δ* | a |
| YER121W | *yer121wΔ* | a |
| YER122C | *glo3Δ* | a |
| YER123W | *yck3Δ* | a |
| YER124C | *dse1Δ* | a |
| YER128W | *yer128wΔ* | a |
| YER129W | *sak1Δ* | a |
| YER130C | *yer130cΔ* | a |
| YER131W | *rps26bΔ* | a |
| YER132C | *pmd1Δ* | a |
| YER133W | *glc7-5-ts* | c |
| YER134C | *yer134cΔ* | a |
| YER135C | *yer135cΔ* | a |
| YER137C | *yer137cΔ* | a |
| YER139C | *rtr1Δ* | a |
| YER140W | *yer140wΔ* | a |
| YER141W | *cox15Δ* | a |
| YER142C | *mag1Δ* | a |
| YER143W | *ddi1Δ* | a |
| YER144C | *ubp5Δ* | a |
| YER145C | *ftr1Δ* | a |
| YER147C | *scc4-ts* | b |
| YER149C | *pea2Δ* | a |
| YER150W | *spi1Δ* | a |
| YER151C | *ubp3Δ* | a |
| YER152C | *yer152cΔ* | a |
| YER153C | *pet122Δ* | a |
| YER154W | *oxa1Δ* | a |
| YER155C | *bem2Δ* | a |
| YER155C | *bem2Δ* | a |
| YER156C | *yer156cΔ* | a |
| YER158C | *yer158cΔ* | a |
| YER161C | *spt2Δ* | a |
| YER162C | *rad4Δ* | a |
| YER163C | *yer163cΔ* | a |
| YER164W | *chd1Δ* | a |
| YER165W | *pab1-ts* | b |
| YER166W | *dnf1Δ* | a |
| YER167W | *bck2Δ* | a |
| YER168C | *cca1-ts* | b |
| YER169W | *rph1Δ* | a |
| YER170W | *adk2Δ* | a |
| YER173W | *rad24Δ* | a |
| YER174C | *grx4Δ* | a |
| YER175C | *tmt1Δ* | a |
| YER175W-A | *yer175w-aΔ* | a |
| YER176W | *ecm32Δ* | a |
| YER177W | *bmh1Δ* | a |
| YER178W | *pda1Δ* | a |
| YER179W | *dmc1Δ* | a |
| YER180C | *isc10Δ* | a |
| YER180C-A | *slo1Δ* | a |
| YER181C | *yer181cΔ* | a |
| YER182W | *fmp10Δ* | a |
| YER183C | *fau1Δ* | a |
| YER184C | *yer184cΔ* | a |
| YER185W | *pug1Δ* | a |
| YER186C | *yer186cΔ* | a |
| YER186W-A | *yer186w-aΔ* | a |
| YER187W | *yer187wΔ* | a |
| YER188W | *yer188wΔ* | a |
| YFL001W | *deg1Δ* | a |
| YFL002C | *spb4-ts* | b |
| YFL003C | *msh4Δ* | a |
| YFL004W | *vtc2Δ* | a |
| YFL006W | *yfl006wΔ* | a |
| YFL007W | *blm10Δ* | a |
| YFL009W | *cdc4-ts* | b |
| YFL010C | *wwm1Δ* | a |
| YFL010W-A | *aua1Δ* | a |
| YFL010W-A | *aua1Δ* | a |
| YFL011W | *hxt10Δ* | a |
| YFL012W | *yfl012wΔ* | a |
| YFL013C | *ies1Δ* | a |
| YFL013W-A | *yfl013w-aΔ* | a |
| YFL014W | *hsp12Δ* | a |
| YFL015C | *yfl015cΔ* | a |
| YFL016C | *mdj1Δ* | a |
| YFL017C | *gna1-ts* | b |
| YFL018C | *lpd1Δ* | a |
| YFL019C | *yfl019cΔ* | a |
| YFL020C | *pau5Δ* | a |
| YFL021W | *gat1Δ* | a |
| YFL022C | *frs2-ts* | b |
| YFL023W | *bud27Δ* | a |
| YFL025C | *bst1Δ* | a |
| YFL026W | *ste2Δ* | a |
| YFL027C | *gyp8Δ* | a |
| YFL028C | *caf16Δ* | a |
| YFL030W | *agx1Δ* | a |
| YFL031W | *hac1Δ* | a |
| YFL031W | *hac1Δ* | a |
| YFL032W | *yfl032wΔ* | a |
| YFL033C | *rim15Δ* | a |
| YFL033C | *rim15Δ* | a |
| YFL034C-A | *rpl22bΔ* | a |
| YFL034W | *yfl034wΔ* | a |
| YFL035C | *yfl035c-ts* | b |
| YFL035C-B | *yfl035c-bΔ* | a |
| YFL036W | *rpo41Δ* | a |
| YFL040W | *yfl040wΔ* | a |
| YFL041W | *fet5Δ* | a |
| YFL041W-A | *yfl041w-aΔ* | a |
| YFL042C | *yfl042cΔ* | a |
| YFL042C | *yfl042cΔ* | a |
| YFL043C | *yfl043cΔ* | a |
| YFL044C | *otu1Δ* | a |
| YFL046W | *fmp32Δ* | a |
| YFL047W | *rgd2Δ* | a |
| YFL048C | *emp47Δ* | a |
| YFL049W | *swp82Δ* | a |
| YFL050C | *alr2Δ* | a |
| YFL051C | *yfl051cΔ* | a |
| YFL052W | *yfl052wΔ* | a |
| YFL053W | *dak2Δ* | a |
| YFL054C | *yfl054cΔ* | a |
| YFL055W | *agp3Δ* | a |
| YFL056C | *aad6Δ* | a |
| YFL063W | *yfl063wΔ* | a |
| YFR001W | *loc1Δ* | a |
| YFR006W | *yfr006wΔ* | a |
| YFR007W | *yfh7Δ* | a |
| YFR008W | *far7Δ* | a |
| YFR009W | *gcn20Δ* | a |
| YFR010W | *ubp6Δ* | a |
| YFR011C | *aim13Δ* | a |
| YFR012W | *yfr012wΔ* | a |
| YFR012W-A | *yfr012w-aΔ* | a |
| YFR013W | *ioc3Δ* | a |
| YFR014C | *cmk1Δ* | a |
| YFR015C | *gsy1Δ* | a |
| YFR016C | *yfr016cΔ* | a |
| YFR017C | *yfr017cΔ* | a |
| YFR018C | *yfr018cΔ* | a |
| YFR019W | *fab1Δ* | a |
| YFR020W | *yfr020wΔ* | a |
| YFR021W | *atg18Δ* | a |
| YFR022W | *rog3Δ* | a |
| YFR023W | *pes4Δ* | a |
| YFR024C | *yfr024cΔ* | a |
| YFR024C-A | *lsb3Δ* | a |
| YFR025C | *his2Δ* | a |
| YFR026C | *uli1Δ* | a |
| YFR030W | *met10Δ* | a |
| YFR031C-A | *rpl2aΔ* | a |
| YFR032C | *rrt5Δ* | a |
| YFR032C-A | *rpl29Δ* | a |
| YFR032C-B | *yfr032c-bΔ* | a |
| YFR033C | *qcr6Δ* | a |
| YFR034C | *pho4Δ* | a |
| YFR035C | *yfr035cΔ* | a |
| YFR036W | *cdc26Δ* | a |
| YFR038W | *irc5Δ* | a |
| YFR038W | *irc5Δ* | a |
| YFR039C | *yfr039cΔ* | a |
| YFR040W | *sap155Δ* | a |
| YFR041C | *erj5Δ* | a |
| YFR042W | *keg1-ts* | b |
| YFR043C | *irc6Δ* | a |
| YFR044C | *dug1Δ* | a |
| YFR045W | *yfr045wΔ* | a |
| YFR045W | *yfr045wΔ* | a |
| YFR046C | *cnn1Δ* | a |
| YFR047C | *bna6Δ* | a |
| YFR048W | *rmd8Δ* | a |
| YFR049W | *ymr31Δ* | a |
| YFR050C | *pre4-ts* | b |
| YFR053C | *hxk1Δ* | a |
| YFR054C | *yfr054cΔ* | a |
| YFR055W | *irc7Δ* | a |
| YFR056C | *yfr056cΔ* | a |
| YFR057W | *yfr057wΔ* | a |
| YGL002W | *erp6Δ* | a |
| YGL003C | *cdh1Δ* | a |
| YGL004C | *rpn14Δ* | a |
| YGL005C | *cog7Δ* | a |
| YGL006W | *pmc1Δ* | a |
| YGL006W-A | *ygl006w-aΔ* | a |
| YGL007C-A | *ygl007c-aΔ* | a |
| YGL007W | *brp1Δ* | a |
| YGL008C | *pma1-ts* | b |
| YGL010W | *ygl010wΔ* | a |
| YGL011C | *scl1-ts* | b |
| YGL012W | *erg4Δ* | a |
| YGL013C | *pdr1Δ* | a |
| YGL014W | *puf4Δ* | a |
| YGL015C | *ygl015cΔ* | a |
| YGL016W | *kap122Δ* | a |
| YGL017W | *ate1Δ* | a |
| YGL019W | *ckb1Δ* | a |
| YGL020C | *get1Δ* | a |
| YGL021W | *alk1Δ* | a |
| YGL023C | *pib2Δ* | a |
| YGL023C | *pib2Δ* | a |
| YGL024W | *ygl024wΔ* | a |
| YGL025C | *pgd1Δ* | a |
| YGL026C | *trp5Δ* | a |
| YGL027C | *cwh41Δ* | a |
| YGL028C | *scw11Δ* | a |
| YGL029W | *cgr1Δ* | a |
| YGL030W | *rpl30-ts* | b |
| YGL031C | *rpl24aΔ* | a |
| YGL032C | *aga2Δ* | a |
| YGL032C | *aga2Δ* | a |
| YGL033W | *hop2Δ* | a |
| YGL033W | *hop2Δ* | a |
| YGL034C | *ygl034cΔ* | a |
| YGL035C | *mig1Δ* | a |
| YGL036W | *ygl036wΔ* | a |
| YGL037C | *pnc1Δ* | a |
| YGL038C | *och1Δ* | a |
| YGL039W | *ygl039wΔ* | a |
| YGL041C | *ygl041cΔ* | a |
| YGL041C-B | *ygl041c-bΔ* | a |
| YGL042C | *ygl042cΔ* | a |
| YGL043W | *dst1Δ* | a |
| YGL044C | *rna15-58* | c |
| YGL045W | *rim8Δ* | a |
| YGL045W | *rim8Δ* | a |
| YGL046W | *ygl046wΔ* | a |
| YGL047W | *alg13-ts* | b |
| YGL049C | *tif4632Δ* | a |
| YGL050W | *tyw3Δ* | a |
| YGL051W | *mst27Δ* | a |
| YGL053W | *prm8Δ* | a |
| YGL054C | *erv14Δ* | a |
| YGL056C | *sds23Δ* | a |
| YGL057C | *gep7Δ* | a |
| YGL058W | *rad6Δ* | a |
| YGL059W | *pkp2Δ* | a |
| YGL060W | *ybp2Δ* | a |
| YGL062W | *pyc1Δ* | a |
| YGL063W | *pus2Δ* | a |
| YGL064C | *mrh4Δ* | a |
| YGL066W | *sgf73Δ* | a |
| YGL067W | *npy1Δ* | a |
| YGL070C | *rpb9Δ* | a |
| YGL071W | *aft1Δ* | a |
| YGL072C | *ygl072cΔ* | a |
| YGL076C | *rpl7aΔ* | a |
| YGL077C | *hnm1Δ* | a |
| YGL078C | *dbp3Δ* | a |
| YGL079W | *ygl079wΔ* | a |
| YGL080W | *fmp37Δ* | a |
| YGL081W | *ygl081wΔ* | a |
| YGL081W | *ygl081wΔ* | a |
| YGL082W | *ygl082wΔ* | a |
| YGL083W | *scy1Δ* | a |
| YGL084C | *gup1Δ* | a |
| YGL085W | *ygl085wΔ* | a |
| YGL086W | *mad1Δ* | a |
| YGL087C | *mms2Δ* | a |
| YGL088W | *ygl088wΔ* | a |
| YGL089C | *mf(alpha)2Δ* | a |
| YGL090W | *lif1Δ* | a |
| YGL091C | *nbp35-ts* | b |
| YGL094C | *pan2Δ* | a |
| YGL095C | *vps45Δ* | a |
| YGL096W | *tos8Δ* | a |
| YGL097W | *prp20-1* | c |
| YGL100W | *seh1Δ* | a |
| YGL101W | *ygl101wΔ* | a |
| YGL101W | *ygl101wΔ* | a |
| YGL104C | *vps73Δ* | a |
| YGL104C | *vps73Δ* | a |
| YGL105W | *arc1Δ* | a |
| YGL107C | *rmd9Δ* | a |
| YGL108C | *ygl108cΔ* | a |
| YGL109W | *ygl109wΔ* | a |
| YGL110C | *cue3Δ* | a |
| YGL111W | *nsa1-ts* | b |
| YGL113W | *sld3-ts* | b |
| YGL114W | *ygl114wΔ* | a |
| YGL115W | *snf4Δ* | a |
| YGL117W | *ygl117wΔ* | a |
| YGL118C | *ygl118cΔ* | a |
| YGL119W | *abc1Δ* | a |
| YGL121C | *gpg1Δ* | a |
| YGL124C | *mon1Δ* | a |
| YGL125W | *met13Δ* | a |
| YGL126W | *scs3Δ* | a |
| YGL127C | *soh1Δ* | a |
| YGL129C | *rsm23Δ* | a |
| YGL131C | *snt2Δ* | a |
| YGL132W | *ygl132wΔ* | a |
| YGL133W | *itc1Δ* | a |
| YGL134W | *pcl10Δ* | a |
| YGL135W | *rpl1bΔ* | a |
| YGL136C | *mrm2Δ* | a |
| YGL138C | *ygl138cΔ* | a |
| YGL139W | *flc3Δ* | a |
| YGL140C | *ygl140cΔ* | a |
| YGL141W | *hul5Δ* | a |
| YGL142C | *gpi10-ts* | b |
| YGL143C | *mrf1Δ* | a |
| YGL144C | *rog1Δ* | a |
| YGL146C | *rrt6Δ* | a |
| YGL147C | *rpl9aΔ* | a |
| YGL148W | *aro2Δ* | a |
| YGL149W | *ygl149wΔ* | a |
| YGL151W | *nut1Δ* | a |
| YGL152C | *ygl152cΔ* | a |
| YGL153W | *pex14Δ* | a |
| YGL154C | *lys5Δ* | a |
| YGL156W | *ams1Δ* | a |
| YGL157W | *ygl157wΔ* | a |
| YGL158W | *rck1Δ* | a |
| YGL159W | *ygl159wΔ* | a |
| YGL160W | *aim14Δ* | a |
| YGL161C | *yip5Δ* | a |
| YGL162W | *sut1Δ* | a |
| YGL163C | *rad54Δ* | a |
| YGL164C | *yrb30Δ* | a |
| YGL165C | *ygl165cΔ* | a |
| YGL166W | *cup2Δ* | a |
| YGL167C | *pmr1Δ* | a |
| YGL168W | *hur1Δ* | a |
| YGL170C | *spo74Δ* | a |
| YGL173C | *kem1Δ* | a |
| YGL174W | *bud13Δ* | a |
| YGL175C | *sae2Δ* | a |
| YGL176C | *ygl176cΔ* | a |
| YGL177W | *ygl177wΔ* | a |
| YGL178W | *mpt5Δ* | a |
| YGL179C | *tos3Δ* | a |
| YGL180W | *atg1Δ* | a |
| YGL181W | *gts1Δ* | a |
| YGL184C | *str3Δ* | a |
| YGL185C | *ygl185cΔ* | a |
| YGL186C | *tpn1Δ* | a |
| YGL188C-A | *ygl188c-aΔ* | a |
| YGL190C | *cdc55Δ* | a |
| YGL191W | *cox13Δ* | a |
| YGL192W | *ime4Δ* | a |
| YGL194C | *hos2Δ* | a |
| YGL195W | *gcn1Δ* | a |
| YGL196W | *dsd1Δ* | a |
| YGL196W | *dsd1Δ* | a |
| YGL197W | *mds3Δ* | a |
| YGL198W | *yip4Δ* | a |
| YGL199C | *ygl199cΔ* | a |
| YGL199C | *ygl199cΔ* | a |
| YGL200C | *emp24Δ* | a |
| YGL201C | *mcm6-ts* | b |
| YGL202W | *aro8Δ* | a |
| YGL202W | *aro8Δ* | a |
| YGL203C | *kex1Δ* | a |
| YGL205W | *pox1Δ* | a |
| YGL206C | *chc1Δ* | a |
| YGL208W | *sip2Δ* | a |
| YGL209W | *mig2Δ* | a |
| YGL210W | *ypt32Δ* | a |
| YGL211W | *ncs6Δ* | a |
| YGL211W | *ncs6Δ* | a |
| YGL212W | *vam7Δ* | a |
| YGL213C | *ski8Δ* | a |
| YGL214W | *ygl214wΔ* | a |
| YGL214W | *ygl214wΔ* | a |
| YGL215W | *clg1Δ* | a |
| YGL216W | *kip3Δ* | a |
| YGL216W | *kip3Δ* | a |
| YGL217C | *ygl217cΔ* | a |
| YGL217C | *ygl217cΔ* | a |
| YGL218W | *ygl218wΔ* | a |
| YGL218W | *ygl218wΔ* | a |
| YGL219C | *mdm34Δ* | a |
| YGL220W | *fra2Δ* | a |
| YGL221C | *nif3Δ* | a |
| YGL222C | *edc1Δ* | a |
| YGL223C | *cog1Δ* | a |
| YGL224C | *sdt1Δ* | a |
| YGL224C | *sdt1Δ* | a |
| YGL226C-A | *ost5Δ* | a |
| YGL226W | *mtc3Δ* | a |
| YGL227W | *vid30Δ* | a |
| YGL228W | *she10Δ* | a |
| YGL229C | *sap4Δ* | a |
| YGL230C | *ygl230cΔ* | a |
| YGL231C | *emc4Δ* | a |
| YGL232W | *tan1Δ* | a |
| YGL234W | *ade5,7Δ* | a |
| YGL235W | *ygl235wΔ* | a |
| YGL235W | *ygl235wΔ* | a |
| YGL236C | *mto1Δ* | a |
| YGL237C | *hap2Δ* | a |
| YGL237C | *hap2Δ* | a |
| YGL238W | *cse1-ts* | b |
| YGL240W | *doc1Δ* | a |
| YGL241W | *kap114Δ* | a |
| YGL242C | *ygl242cΔ* | a |
| YGL243W | *tad1Δ* | a |
| YGL244W | *rtf1Δ* | a |
| YGL245W | *gus1-ts* | b |
| YGL246C | *rai1Δ* | a |
| YGL247W | *brr6-ts* | b |
| YGL248W | *pde1Δ* | a |
| YGL249W | *zip2Δ* | a |
| YGL250W | *rmr1Δ* | a |
| YGL251C | *hfm1Δ* | a |
| YGL252C | *rtg2Δ* | a |
| YGL253W | *hxk2Δ* | a |
| YGL254W | *fzf1Δ* | a |
| YGL255W | *zrt1Δ* | a |
| YGL256W | *adh4Δ* | a |
| YGL257C | *mnt2Δ* | a |
| YGL258W | *vel1Δ* | a |
| YGL259W | *yps5Δ* | a |
| YGL260W | *ygl260wΔ* | a |
| YGL261C | *ygl261cΔ* | a |
| YGL262W | *ygl262wΔ* | a |
| YGL263W | *cos12Δ* | a |
| YGR001C | *ygr001cΔ* | a |
| YGR003W | *cul3Δ* | a |
| YGR004W | *pex31Δ* | a |
| YGR005C | *tfg2-ts* | b |
| YGR006W | *prp18-1* | c |
| YGR006W | *prp18Δ* | a |
| YGR007W | *muq1Δ* | a |
| YGR008C | *stf2Δ* | a |
| YGR010W | *nma2Δ* | a |
| YGR011W | *ygr011wΔ* | a |
| YGR011W | *ygr011wΔ* | a |
| YGR012W | *ygr012wΔ* | a |
| YGR013W | *snu71-ts* | b |
| YGR014W | *msb2Δ* | a |
| YGR015C | *ygr015cΔ* | a |
| YGR016W | *ygr016wΔ* | a |
| YGR017W | *ygr017wΔ* | a |
| YGR018C | *ygr018cΔ* | a |
| YGR018C | *ygr018cΔ* | a |
| YGR019W | *uga1Δ* | a |
| YGR020C | *vma7Δ* | a |
| YGR021W | *ygr021wΔ* | a |
| YGR022C | *ygr022cΔ* | a |
| YGR022C | *ygr022cΔ* | a |
| YGR023W | *mtl1Δ* | a |
| YGR024C | *thg1-ts* | b |
| YGR025W | *ygr025wΔ* | a |
| YGR025W | *ygr025wΔ* | a |
| YGR026W | *ygr026wΔ* | a |
| YGR027C | *rps25aΔ* | a |
| YGR028W | *msp1Δ* | a |
| YGR029W | *erv1-ts* | b |
| YGR031W | *ygr031wΔ* | a |
| YGR032W | *gsc2Δ* | a |
| YGR033C | *tim21Δ* | a |
| YGR034W | *rpl26bΔ* | a |
| YGR035C | *ygr035cΔ* | a |
| YGR035W-A | *ygr035w-aΔ* | a |
| YGR036C | *cax4Δ* | a |
| YGR037C | *acb1Δ* | a |
| YGR037C | *acb1Δ* | a |
| YGR038W | *orm1Δ* | a |
| YGR039W | *ygr039wΔ* | a |
| YGR040W | *kss1Δ* | a |
| YGR041W | *bud9Δ* | a |
| YGR042W | *ygr042wΔ* | a |
| YGR043C | *nqm1Δ* | a |
| YGR044C | *rme1Δ* | a |
| YGR045C | *ygr045cΔ* | a |
| YGR046W | *tam41-ts* | b |
| YGR047C | *tfc4-ts* | b |
| YGR049W | *scm4Δ* | a |
| YGR050C | *ygr050cΔ* | a |
| YGR051C | *ygr051cΔ* | a |
| YGR052W | *fmp48Δ* | a |
| YGR053C | *ygr053cΔ* | a |
| YGR054W | *ygr054wΔ* | a |
| YGR055W | *mup1Δ* | a |
| YGR056W | *rsc1Δ* | a |
| YGR057C | *lst7Δ* | a |
| YGR058W | *pef1Δ* | a |
| YGR059W | *spr3Δ* | a |
| YGR061C | *ade6Δ* | a |
| YGR062C | *cox18Δ* | a |
| YGR062C | *cox18Δ* | a |
| YGR063C | *spt4Δ* | a |
| YGR064W | *ygr064wΔ* | a |
| YGR066C | *ygr066cΔ* | a |
| YGR067C | *ygr067cΔ* | a |
| YGR068C | *art5Δ* | a |
| YGR069W | *ygr069wΔ* | a |
| YGR070W | *rom1Δ* | a |
| YGR071C | *ygr071cΔ* | a |
| YGR072W | *upf3Δ* | a |
| YGR076C | *mrpl25Δ* | a |
| YGR077C | *pex8Δ* | a |
| YGR078C | *pac10Δ* | a |
| YGR079W | *ygr079wΔ* | a |
| YGR080W | *twf1Δ* | a |
| YGR081C | *slx9Δ* | a |
| YGR084C | *mrp13Δ* | a |
| YGR085C | *rpl11bΔ* | a |
| YGR086C | *pil1Δ* | a |
| YGR087C | *pdc6Δ* | a |
| YGR088W | *ctt1Δ* | a |
| YGR089W | *nnf2Δ* | a |
| YGR092W | *dbf2Δ* | a |
| YGR093W | *ygr093wΔ* | a |
| YGR094W | *vas1-ts* | b |
| YGR096W | *tpc1Δ* | a |
| YGR097W | *ask10Δ* | a |
| YGR100W | *mdr1Δ* | a |
| YGR101W | *pcp1Δ* | a |
| YGR102C | *ygr102cΔ* | a |
| YGR104C | *srb5Δ* | a |
| YGR105W | *vma21Δ* | a |
| YGR106C | *voa1Δ* | a |
| YGR107W | *ygr107wΔ* | a |
| YGR108W | *clb1Δ* | a |
| YGR109C | *clb6Δ* | a |
| YGR110W | *cld1Δ* | a |
| YGR111W | *ygr111wΔ* | a |
| YGR112W | *shy1Δ* | a |
| YGR117C | *ygr117cΔ* | a |
| YGR118W | *rps23aΔ* | a |
| YGR119C | *nup57-ts* | b |
| YGR121C | *mep1Δ* | a |
| YGR121W-A | *ygr121w-aΔ* | a |
| YGR122C-A | *ygr122c-aΔ* | a |
| YGR122W | *ygr122wΔ* | a |
| YGR123C | *ppt1Δ* | a |
| YGR124W | *asn2Δ* | a |
| YGR125W | *ygr125wΔ* | a |
| YGR126W | *ygr126wΔ* | a |
| YGR127W | *ygr127wΔ* | a |
| YGR128C | *utp8-ts* | b |
| YGR129W | *syf2Δ* | a |
| YGR130C | *ygr130cΔ* | a |
| YGR131W | *ygr131wΔ* | a |
| YGR132C | *phb1Δ* | a |
| YGR133W | *pex4Δ* | a |
| YGR134W | *caf130Δ* | a |
| YGR135W | *pre9Δ* | a |
| YGR136W | *lsb1Δ* | a |
| YGR137W | *ygr137wΔ* | a |
| YGR138C | *tpo2Δ* | a |
| YGR139W | *ygr139wΔ* | a |
| YGR141W | *vps62Δ* | a |
| YGR142W | *btn2Δ* | a |
| YGR143W | *skn1Δ* | a |
| YGR144W | *thi4Δ* | a |
| YGR145W | *enp2-ts* | b |
| YGR146C | *ygr146cΔ* | a |
| YGR146C-A | *ygr146c-aΔ* | a |
| YGR148C | *rpl24bΔ* | a |
| YGR149W | *ygr149wΔ* | a |
| YGR150C | *ygr150cΔ* | a |
| YGR151C | *ygr151cΔ* | a |
| YGR152C | *rsr1Δ* | a |
| YGR153W | *ygr153wΔ* | a |
| YGR154C | *gto1Δ* | a |
| YGR155W | *cys4Δ* | a |
| YGR157W | *cho2Δ* | a |
| YGR159C | *nsr1Δ* | a |
| YGR160W | *ygr160wΔ* | a |
| YGR161C | *rts3Δ* | a |
| YGR161W-C | *ygr161w-cΔ* | a |
| YGR162W | *tif4631Δ* | a |
| YGR163W | *gtr2Δ* | a |
| YGR164W | *ygr164wΔ* | a |
| YGR165W | *mrps35Δ* | a |
| YGR166W | *kre11Δ* | a |
| YGR167W | *clc1Δ* | a |
| YGR168C | *ygr168cΔ* | a |
| YGR169C | *pus6Δ* | a |
| YGR169C-A | *ygr169c-aΔ* | a |
| YGR170W | *psd2Δ* | a |
| YGR171C | *msm1Δ* | a |
| YGR172C | *yip1-ts* | b |
| YGR173W | *rbg2Δ* | a |
| YGR174C | *cbp4Δ* | a |
| YGR174W-A | *ygr174w-aΔ* | a |
| YGR176W | *ygr176wΔ* | a |
| YGR177C | *atf2Δ* | a |
| YGR178C | *pbp1Δ* | a |
| YGR180C | *rnr4Δ* | a |
| YGR180C | *rnr4Δ* | a |
| YGR181W | *tim13Δ* | a |
| YGR182C | *ygr182cΔ* | a |
| YGR183C | *qcr9Δ* | a |
| YGR184C | *ubr1Δ* | a |
| YGR187C | *hgh1Δ* | a |
| YGR188C | *bub1Δ* | a |
| YGR189C | *crh1Δ* | a |
| YGR192C | *tdh3Δ* | a |
| YGR193C | *pdx1Δ* | a |
| YGR194C | *xks1Δ* | a |
| YGR195W | *ski6-ts* | b |
| YGR196C | *fyv8Δ* | a |
| YGR197C | *sng1Δ* | a |
| YGR199W | *pmt6Δ* | a |
| YGR200C | *elp2Δ* | a |
| YGR201C | *ygr201cΔ* | a |
| YGR202C | *pct1Δ* | a |
| YGR203W | *ygr203wΔ* | a |
| YGR204C-A | *ygr204c-aΔ* | a |
| YGR204W | *ade3Δ* | a |
| YGR205W | *ygr205wΔ* | a |
| YGR206W | *mvb12Δ* | a |
| YGR207C | *ygr207cΔ* | a |
| YGR208W | *ser2Δ* | a |
| YGR209C | *trx2Δ* | a |
| YGR210C | *ygr210cΔ* | a |
| YGR212W | *sli1Δ* | a |
| YGR213C | *rta1Δ* | a |
| YGR214W | *rps0aΔ* | a |
| YGR215W | *rsm27Δ* | a |
| YGR217W | *cch1Δ* | a |
| YGR219W | *ygr219wΔ* | a |
| YGR220C | *mrpl9Δ* | a |
| YGR221C | *tos2Δ* | a |
| YGR222W | *pet54Δ* | a |
| YGR223C | *hsv2Δ* | a |
| YGR224W | *azr1Δ* | a |
| YGR225W | *ama1Δ* | a |
| YGR225W | *ama1Δ* | a |
| YGR226C | *ygr226cΔ* | a |
| YGR227W | *die2Δ* | a |
| YGR228W | *ygr228wΔ* | a |
| YGR229C | *smi1Δ* | a |
| YGR230W | *bns1Δ* | a |
| YGR231C | *phb2Δ* | a |
| YGR232W | *nas6Δ* | a |
| YGR233C | *pho81Δ* | a |
| YGR234W | *yhb1Δ* | a |
| YGR235C | *ygr235cΔ* | a |
| YGR236C | *spg1Δ* | a |
| YGR237C | *ygr237cΔ* | a |
| YGR238C | *kel2Δ* | a |
| YGR239C | *pex21Δ* | a |
| YGR240C | *pfk1Δ* | a |
| YGR241C | *yap1802Δ* | a |
| YGR242W | *ygr242wΔ* | a |
| YGR243W | *fmp43Δ* | a |
| YGR244C | *lsc2Δ* | a |
| YGR244C | *lsc2Δ* | a |
| YGR247W | *cpd1Δ* | a |
| YGR248W | *sol4Δ* | a |
| YGR249W | *mga1Δ* | a |
| YGR250C | *ygr250cΔ* | a |
| YGR252W | *gcn5Δ* | a |
| YGR253C | *pup2-ts* | b |
| YGR254W | *eno1Δ* | a |
| YGR255C | *coq6Δ* | a |
| YGR256W | *gnd2Δ* | a |
| YGR257C | *mtm1Δ* | a |
| YGR258C | *rad2Δ* | a |
| YGR259C | *ygr259cΔ* | a |
| YGR260W | *tna1Δ* | a |
| YGR261C | *apl6Δ* | a |
| YGR262C | *bud32Δ* | a |
| YGR263C | *say1Δ* | a |
| YGR266W | *ygr266wΔ* | a |
| YGR268C | *hua1Δ* | a |
| YGR269W | *ygr269wΔ* | a |
| YGR270W | *yta7Δ* | a |
| YGR271C-A | *efg1Δ* | a |
| YGR271W | *slh1Δ* | a |
| YGR272C | *ygr272cΔ* | a |
| YGR273C | *ygr273cΔ* | a |
| YGR275W | *rtt102Δ* | a |
| YGR276C | *rnh70Δ* | a |
| YGR277C | *cab4-ts* | b |
| YGR278W | *cwc22-ts* | b |
| YGR279C | *scw4Δ* | a |
| YGR281W | *yor1Δ* | a |
| YGR282C | *bgl2Δ* | a |
| YGR283C | *ygr283cΔ* | a |
| YGR284C | *erv29Δ* | a |
| YGR285C | *zuo1Δ* | a |
| YGR286C | *bio2Δ* | a |
| YGR287C | *ygr287cΔ* | a |
| YGR288W | *mal13Δ* | a |
| YGR289C | *mal11Δ* | a |
| YGR290W | *ygr290wΔ* | a |
| YGR291C | *ygr291cΔ* | a |
| YGR292W | *mal12Δ* | a |
| YGR295C | *cos6Δ* | a |
| YHL001W | *rpl14bΔ* | a |
| YHL002W | *hse1Δ* | a |
| YHL003C | *lag1Δ* | a |
| YHL004W | *mrp4Δ* | a |
| YHL005C | *yhl005cΔ* | a |
| YHL006C | *shu1Δ* | a |
| YHL007C | *ste20Δ* | a |
| YHL008C | *yhl008cΔ* | a |
| YHL009C | *yap3Δ* | a |
| YHL010C | *yhl010cΔ* | a |
| YHL011C | *prs3Δ* | a |
| YHL012W | *yhl012wΔ* | a |
| YHL013C | *otu2Δ* | a |
| YHL014C | *ylf2Δ* | a |
| YHL015W-A | *yhl015w-aΔ* | a |
| YHL016C | *dur3Δ* | a |
| YHL017W | *yhl017wΔ* | a |
| YHL019C | *apm2Δ* | a |
| YHL020C | *opi1Δ* | a |
| YHL021C | *aim17Δ* | a |
| YHL022C | *spo11Δ* | a |
| YHL023C | *rmd11Δ* | a |
| YHL024W | *rim4Δ* | a |
| YHL025W | *snf6Δ* | a |
| YHL026C | *yhl026cΔ* | a |
| YHL027W | *rim101Δ* | a |
| YHL028W | *wsc4Δ* | a |
| YHL029C | *oca5Δ* | a |
| YHL030W | *ecm29Δ* | a |
| YHL031C | *gos1Δ* | a |
| YHL032C | *gut1Δ* | a |
| YHL033C | *rpl8aΔ* | a |
| YHL034C | *sbp1Δ* | a |
| YHL035C | *vmr1Δ* | a |
| YHL036W | *mup3Δ* | a |
| YHL037C | *yhl037cΔ* | a |
| YHL038C | *cbp2Δ* | a |
| YHL039W | *yhl039wΔ* | a |
| YHL040C | *arn1Δ* | a |
| YHL041W | *yhl041wΔ* | a |
| YHL042W | *yhl042wΔ* | a |
| YHL043W | *ecm34Δ* | a |
| YHL044W | *yhl044wΔ* | a |
| YHL045W | *yhl045wΔ* | a |
| YHL046C | *yhl046cΔ* | a |
| YHL047C | *arn2Δ* | a |
| YHR001W | *osh7Δ* | a |
| YHR001W-A | *qcr10Δ* | a |
| YHR003C | *yhr003cΔ* | a |
| YHR004C | *nem1Δ* | a |
| YHR005C | *gpa1Δ* | a |
| YHR006W | *stp2Δ* | a |
| YHR007C-A | *yhr007c-aΔ* | a |
| YHR008C | *sod2Δ* | a |
| YHR009C | *yhr009cΔ* | a |
| YHR010W | *rpl27aΔ* | a |
| YHR011W | *dia4Δ* | a |
| YHR012W | *vps29Δ* | a |
| YHR013C | *ard1Δ* | a |
| YHR014W | *spo13Δ* | a |
| YHR015W | *mip6Δ* | a |
| YHR016C | *ysc84Δ* | a |
| YHR017W | *ysc83Δ* | a |
| YHR018C | *arg4Δ* | a |
| YHR019C | *ded81-ts* | b |
| YHR020W | *yhr020w-ts* | b |
| YHR021C | *rps27bΔ* | a |
| YHR021W-A | *ecm12Δ* | a |
| YHR022C | *yhr022cΔ* | a |
| YHR022C-A | *yhr022c-aΔ* | a |
| YHR025W | *thr1Δ* | a |
| YHR026W | *ppa1Δ* | a |
| YHR028C | *dap2Δ* | a |
| YHR029C | *yhi9Δ* | a |
| YHR030C | *slt2Δ* | a |
| YHR031C | *rrm3Δ* | a |
| YHR032W | *yhr032wΔ* | a |
| YHR033W | *yhr033wΔ* | a |
| YHR034C | *pih1Δ* | a |
| YHR035W | *yhr035wΔ* | a |
| YHR036W | *brl1-ts* | b |
| YHR037W | *put2Δ* | a |
| YHR038W | *rrf1Δ* | a |
| YHR039C | *msc7Δ* | a |
| YHR039C-B | *yhr039c-bΔ* | a |
| YHR040W | *bcd1-ts* | b |
| YHR041C | *srb2Δ* | a |
| YHR043C | *dog2Δ* | a |
| YHR044C | *dog1Δ* | a |
| YHR045W | *yhr045wΔ* | a |
| YHR046C | *inm1Δ* | a |
| YHR047C | *aap1Δ* | a |
| YHR048W | *yhk8Δ* | a |
| YHR049C-A | *yhr049c-aΔ* | a |
| YHR049W | *fsh1Δ* | a |
| YHR050W | *smf2Δ* | a |
| YHR050W-A | *yhr050w-aΔ* | a |
| YHR051W | *cox6Δ* | a |
| YHR057C | *cpr2Δ* | a |
| YHR058C | *med6-ts* | b |
| YHR059W | *fyv4Δ* | a |
| YHR060W | *vma22Δ* | a |
| YHR061C | *gic1Δ* | a |
| YHR063C | *pan5Δ* | a |
| YHR064C | *ssz1Δ* | a |
| YHR065C | *rrp3-ts* | b |
| YHR066W | *ssf1Δ* | a |
| YHR067W | *htd2Δ* | a |
| YHR070W | *trm5-ts* | b |
| YHR071W | *pcl5Δ* | a |
| YHR073W | *osh3Δ* | a |
| YHR075C | *ppe1Δ* | a |
| YHR076W | *ptc7Δ* | a |
| YHR077C | *nmd2Δ* | a |
| YHR078W | *yhr078wΔ* | a |
| YHR079C | *ire1Δ* | a |
| YHR079C-B | *yhr079c-bΔ* | a |
| YHR080C | *yhr080cΔ* | a |
| YHR081W | *lrp1Δ* | a |
| YHR082C | *ksp1Δ* | a |
| YHR085W | *ipi1-ts* | b |
| YHR086W | *nam8Δ* | a |
| YHR086W-A | *yhr086w-aΔ* | a |
| YHR087W | *rtc3Δ* | a |
| YHR088W | *rpf1-ts* | b |
| YHR090C | *yng2Δ* | a |
| YHR091C | *msr1Δ* | a |
| YHR091C | *msr1Δ* | a |
| YHR092C | *hxt4Δ* | a |
| YHR093W | *aht1Δ* | a |
| YHR094C | *hxt1Δ* | a |
| YHR095W | *yhr095wΔ* | a |
| YHR096C | *hxt5Δ* | a |
| YHR097C | *yhr097cΔ* | a |
| YHR098C | *sfb3Δ* | a |
| YHR100C | *gep4Δ* | a |
| YHR101C | *big1-ts* | b |
| YHR103W | *sbe22Δ* | a |
| YHR104W | *gre3Δ* | a |
| YHR105W | *ypt35Δ* | a |
| YHR106W | *trr2Δ* | a |
| YHR108W | *gga2Δ* | a |
| YHR109W | *ctm1Δ* | a |
| YHR110W | *erp5Δ* | a |
| YHR111W | *uba4Δ* | a |
| YHR112C | *yhr112cΔ* | a |
| YHR113W | *yhr113wΔ* | a |
| YHR114W | *bzz1Δ* | a |
| YHR115C | *dma1Δ* | a |
| YHR116W | *cox23Δ* | a |
| YHR117W | *tom71Δ* | a |
| YHR118C | *orc6-ts* | b |
| YHR120W | *msh1Δ* | a |
| YHR121W | *lsm12Δ* | a |
| YHR122W | *yhr122w-ts* | b |
| YHR123W | *ept1Δ* | a |
| YHR124W | *ndt80Δ* | a |
| YHR125W | *yhr125wΔ* | a |
| YHR126C | *yhr126cΔ* | a |
| YHR127W | *yhr127wΔ* | a |
| YHR129C | *arp1Δ* | a |
| YHR130C | *yhr130cΔ* | a |
| YHR131C | *yhr131cΔ* | a |
| YHR132C | *ecm14Δ* | a |
| YHR132W-A | *yhr132w-aΔ* | a |
| YHR133C | *nsg1Δ* | a |
| YHR134W | *wss1Δ* | a |
| YHR135C | *yck1Δ* | a |
| YHR136C | *spl2Δ* | a |
| YHR137W | *aro9Δ* | a |
| YHR138C | *yhr138cΔ* | a |
| YHR139C | *sps100Δ* | a |
| YHR139C-A | *yhr139c-aΔ* | a |
| YHR140W | *yhr140wΔ* | a |
| YHR141C | *rpl42bΔ* | a |
| YHR142W | *chs7Δ* | a |
| YHR143W | *dse2Δ* | a |
| YHR146W | *crp1Δ* | a |
| YHR147C | *mrpl6Δ* | a |
| YHR149C | *skg6Δ* | a |
| YHR150W | *pex28Δ* | a |
| YHR151C | *mtc6Δ* | a |
| YHR152W | *spo12Δ* | a |
| YHR153C | *spo16Δ* | a |
| YHR154W | *rtt107Δ* | a |
| YHR155W | *ysp1Δ* | a |
| YHR156C | *lin1Δ* | a |
| YHR157W | *rec104Δ* | a |
| YHR158C | *kel1Δ* | a |
| YHR159W | *yhr159wΔ* | a |
| YHR160C | *pex18Δ* | a |
| YHR161C | *yap1801Δ* | a |
| YHR162W | *yhr162wΔ* | a |
| YHR163W | *sol3Δ* | a |
| YHR165C | *prp8-1* | c |
| YHR167W | *thp2Δ* | a |
| YHR168W | *mtg2Δ* | a |
| YHR171W | *atg7Δ* | a |
| YHR172W | *spc97-ts* | b |
| YHR175W-A | *yhr175w-aΔ* | a |
| YHR176W | *fmo1Δ* | a |
| YHR177W | *yhr177wΔ* | a |
| YHR178W | *stb5Δ* | a |
| YHR179W | *oye2Δ* | a |
| YHR180W | *yhr180wΔ* | a |
| YHR181W | *svp26Δ* | a |
| YHR182W | *yhr182wΔ* | a |
| YHR183W | *gnd1Δ* | a |
| YHR184W | *ssp1Δ* | a |
| YHR185C | *pfs1Δ* | a |
| YHR187W | *iki1Δ* | a |
| YHR188C | *gpi16-ts* | b |
| YHR189W | *pth1Δ* | a |
| YHR190W | *erg9-ts* | b |
| YHR191C | *ctf8Δ* | a |
| YHR192W | *yhr192wΔ* | a |
| YHR193C | *egd2Δ* | a |
| YHR194W | *mdm31Δ* | a |
| YHR195W | *nvj1Δ* | a |
| YHR196W | *utp9-ts* | b |
| YHR197W | *rix1-ts* | b |
| YHR198C | *aim18Δ* | a |
| YHR199C | *aim46Δ* | a |
| YHR200W | *rpn10Δ* | a |
| YHR202W | *yhr202wΔ* | a |
| YHR203C | *rps4bΔ* | a |
| YHR204W | *mnl1Δ* | a |
| YHR205W | *sch9Δ* | a |
| YHR206W | *skn7Δ* | a |
| YHR207C | *set5Δ* | a |
| YHR209W | *crg1Δ* | a |
| YHR210C | *yhr210cΔ* | a |
| YIL001W | *yil001wΔ* | a |
| YIL002C | *inp51Δ* | a |
| YIL002W-A | *yil002w-aΔ* | a |
| YIL005W | *eps1Δ* | a |
| YIL006W | *yia6Δ* | a |
| YIL007C | *nas2Δ* | a |
| YIL008W | *urm1Δ* | a |
| YIL009C-A | *est3Δ* | a |
| YIL009W | *faa3Δ* | a |
| YIL010W | *dot5Δ* | a |
| YIL011W | *tir3Δ* | a |
| YIL012W | *yil012wΔ* | a |
| YIL013C | *pdr11Δ* | a |
| YIL014W | *mnt3Δ* | a |
| YIL015C-A | *yil015c-aΔ* | a |
| YIL015W | *bar1Δ* | a |
| YIL016W | *snl1Δ* | a |
| YIL017C | *vid28Δ* | a |
| YIL018W | *rpl2bΔ* | a |
| YIL020C | *his6Δ* | a |
| YIL022W | *tim44-ts* | b |
| YIL023C | *yke4Δ* | a |
| YIL024C | *yil024cΔ* | a |
| YIL025C | *yil025cΔ* | a |
| YIL027C | *kre27Δ* | a |
| YIL028W | *yil028wΔ* | a |
| YIL029C | *yil029cΔ* | a |
| YIL030C | *ssm4Δ* | a |
| YIL032C | *yil032cΔ* | a |
| YIL034C | *cap2Δ* | a |
| YIL035C | *cka1Δ* | a |
| YIL036W | *cst6Δ* | a |
| YIL037C | *prm2Δ* | a |
| YIL038C | *not3Δ* | a |
| YIL039W | *ted1Δ* | a |
| YIL040W | *apq12Δ* | a |
| YIL041W | *gvp36Δ* | a |
| YIL041W | *gvp36Δ* | a |
| YIL042C | *pkp1Δ* | a |
| YIL043C | *cbr1Δ* | a |
| YIL044C | *age2Δ* | a |
| YIL045W | *pig2Δ* | a |
| YIL046W-A | *yil046w-aΔ* | a |
| YIL047C | *syg1Δ* | a |
| YIL049W | *dfg10Δ* | a |
| YIL050W | *pcl7Δ* | a |
| YIL052C | *rpl34bΔ* | a |
| YIL053W | *rhr2Δ* | a |
| YIL054W | *yil054wΔ* | a |
| YIL055C | *yil055cΔ* | a |
| YIL056W | *vhr1Δ* | a |
| YIL057C | *yil057cΔ* | a |
| YIL058W | *yil058wΔ* | a |
| YIL059C | *yil059cΔ* | a |
| YIL060W | *yil060wΔ* | a |
| YIL061C | *snp1-ts* | b |
| YIL064W | *see1Δ* | a |
| YIL065C | *fis1Δ* | a |
| YIL066C | *rnr3Δ* | a |
| YIL067C | *yil067cΔ* | a |
| YIL069C | *rps24bΔ* | a |
| YIL070C | *mam33Δ* | a |
| YIL071C | *pci8Δ* | a |
| YIL072W | *hop1Δ* | a |
| YIL073C | *spo22Δ* | a |
| YIL074C | *ser33Δ* | a |
| YIL076W | *sec28Δ* | a |
| YIL077C | *yil077cΔ* | a |
| YIL078W | *ths1-ts* | b |
| YIL079C | *air1Δ* | a |
| YIL083C | *cab2-ts* | b |
| YIL084C | *sds3Δ* | a |
| YIL085C | *ktr7Δ* | a |
| YIL086C | *yil086cΔ* | a |
| YIL087C | *aim19Δ* | a |
| YIL088C | *avt7Δ* | a |
| YIL089W | *yil089wΔ* | a |
| YIL090W | *ice2Δ* | a |
| YIL092W | *yil092wΔ* | a |
| YIL092W | *yil092wΔ* | a |
| YIL093C | *rsm25Δ* | a |
| YIL094C | *lys12Δ* | a |
| YIL095W | *prk1Δ* | a |
| YIL096C | *yil096cΔ* | a |
| YIL097W | *fyv10Δ* | a |
| YIL098C | *fmc1Δ* | a |
| YIL099W | *sga1Δ* | a |
| YIL100W | *yil100wΔ* | a |
| YIL101C | *xbp1Δ* | a |
| YIL102C | *yil102cΔ* | a |
| YIL103W | *dph1Δ* | a |
| YIL104C | *shq1-ts* | b |
| YIL105C | *slm1Δ* | a |
| YIL106W | *mob1-ts* | b |
| YIL107C | *pfk26Δ* | a |
| YIL108W | *yil108wΔ* | a |
| YIL110W | *mni1Δ* | a |
| YIL111W | *cox5bΔ* | a |
| YIL112W | *hos4Δ* | a |
| YIL113W | *sdp1Δ* | a |
| YIL114C | *por2Δ* | a |
| YIL116W | *his5Δ* | a |
| YIL117C | *prm5Δ* | a |
| YIL119C | *rpi1Δ* | a |
| YIL120W | *qdr1Δ* | a |
| YIL121W | *qdr2Δ* | a |
| YIL122W | *pog1Δ* | a |
| YIL123W | *sim1Δ* | a |
| YIL124W | *ayr1Δ* | a |
| YIL125W | *kgd1Δ* | a |
| YIL127C | *rrt14Δ* | a |
| YIL128W | *met18Δ* | a |
| YIL130W | *asg1Δ* | a |
| YIL131C | *fkh1Δ* | a |
| YIL132C | *csm2Δ* | a |
| YIL133C | *rpl16aΔ* | a |
| YIL134C-A | *yil134c-aΔ* | a |
| YIL134W | *flx1Δ* | a |
| YIL135C | *vhs2Δ* | a |
| YIL136W | *om45Δ* | a |
| YIL137C | *tma108Δ* | a |
| YIL138C | *tpm2Δ* | a |
| YIL139C | *rev7Δ* | a |
| YIL140W | *axl2Δ* | a |
| YIL141W | *yil141wΔ* | a |
| YIL145C | *pan6Δ* | a |
| YIL146C | *ecm37Δ* | a |
| YIL148W | *rpl40aΔ* | a |
| YIL149C | *mlp2Δ* | a |
| YIL152W | *yil152wΔ* | a |
| YIL153W | *rrd1Δ* | a |
| YIL154C | *imp2'Δ* | a |
| YIL155C | *gut2Δ* | a |
| YIL156W | *ubp7Δ* | a |
| YIL157C | *coa1Δ* | a |
| YIL158W | *aim20Δ* | a |
| YIL159W | *bnr1Δ* | a |
| YIL160C | *pot1Δ* | a |
| YIL161W | *yil161wΔ* | a |
| YIL162W | *suc2Δ* | a |
| YIL163C | *yil163cΔ* | a |
| YIL164C | *nit1Δ* | a |
| YIL165C | *yil165cΔ* | a |
| YIL166C | *yil166cΔ* | a |
| YIL167W | *yil167wΔ* | a |
| YIL168W | *yil168wΔ* | a |
| YIL170W | *yil170wΔ* | a |
| YIL173W | *vth1Δ* | a |
| YIR001C | *sgn1Δ* | a |
| YIR002C | *mph1Δ* | a |
| YIR003W | *aim21Δ* | a |
| YIR004W | *djp1Δ* | a |
| YIR005W | *ist3Δ* | a |
| YIR007W | *yir007wΔ* | a |
| YIR009W | *msl1Δ* | a |
| YIR011C | *sts1-ts* | b |
| YIR013C | *gat4Δ* | a |
| YIR014W | *yir014wΔ* | a |
| YIR016W | *yir016wΔ* | a |
| YIR017C | *met28Δ* | a |
| YIR018C-A | *yir018c-aΔ* | a |
| YIR018W | *yap5Δ* | a |
| YIR019C | *muc1Δ* | a |
| YIR020C | *yir020cΔ* | a |
| YIR020W-B | *yir020w-bΔ* | a |
| YIR021W | *mrs1Δ* | a |
| YIR021W-A | *yir021w-aΔ* | a |
| YIR023W | *dal81Δ* | a |
| YIR024C | *yir024cΔ* | a |
| YIR025W | *mnd2Δ* | a |
| YIR026C | *yvh1Δ* | a |
| YIR027C | *dal1Δ* | a |
| YIR028W | *dal4Δ* | a |
| YIR029W | *dal2Δ* | a |
| YIR030C | *dcg1Δ* | a |
| YIR031C | *dal7Δ* | a |
| YIR032C | *dal3Δ* | a |
| YIR033W | *mga2Δ* | a |
| YIR034C | *lys1Δ* | a |
| YIR035C | *yir035cΔ* | a |
| YIR036C | *irc24Δ* | a |
| YIR037W | *hyr1Δ* | a |
| YIR038C | *gtt1Δ* | a |
| YIR039C | *yps6Δ* | a |
| YIR042C | *yir042cΔ* | a |
| YIR043C | *yir043cΔ* | a |
| YIR044C | *yir044cΔ* | a |
| YJL003W | *cox16Δ* | a |
| YJL004C | *sys1Δ* | a |
| YJL006C | *ctk2Δ* | a |
| YJL007C | *yjl007cΔ* | a |
| YJL010C | *nop9-ts* | b |
| YJL011C | *rpc17-ts* | b |
| YJL012C | *vtc4Δ* | a |
| YJL012C | *vtc4Δ* | a |
| YJL012C-A | *yjl012c-aΔ* | a |
| YJL013C | *mad3Δ* | a |
| YJL016W | *yjl016wΔ* | a |
| YJL016W | *yjl016wΔ* | a |
| YJL017W | *yjl017wΔ* | a |
| YJL020C | *bbc1Δ* | a |
| YJL020C | *bbc1Δ* | a |
| YJL021C | *yjl021cΔ* | a |
| YJL022W | *yjl022wΔ* | a |
| YJL023C | *pet130Δ* | a |
| YJL024C | *aps3Δ* | a |
| YJL025W | *rrn7-ts* | b |
| YJL027C | *yjl027cΔ* | a |
| YJL028W | *yjl028wΔ* | a |
| YJL029C | *vps53Δ* | a |
| YJL030W | *mad2Δ* | a |
| YJL031C | *bet4-ts* | b |
| YJL035C | *tad2-ts* | b |
| YJL036W | *snx4Δ* | a |
| YJL037W | *irc18Δ* | a |
| YJL038C | *loh1Δ* | a |
| YJL042W | *mhp1Δ* | a |
| YJL043W | *yjl043wΔ* | a |
| YJL044C | *gyp6Δ* | a |
| YJL045W | *yjl045wΔ* | a |
| YJL046W | *aim22Δ* | a |
| YJL047C | *rtt101Δ* | a |
| YJL047C-A | *yjl047c-aΔ* | a |
| YJL048C | *ubx6Δ* | a |
| YJL049W | *yjl049wΔ* | a |
| YJL051W | *irc8Δ* | a |
| YJL052W | *tdh1Δ* | a |
| YJL053W | *pep8Δ* | a |
| YJL055W | *yjl055wΔ* | a |
| YJL056C | *zap1Δ* | a |
| YJL057C | *iks1Δ* | a |
| YJL058C | *bit61Δ* | a |
| YJL059W | *yhc3Δ* | a |
| YJL059W | *yhc3Δ* | a |
| YJL060W | *bna3Δ* | a |
| YJL062W | *las21Δ* | a |
| YJL062W-A | *yjl062w-aΔ* | a |
| YJL063C | *mrpl8Δ* | a |
| YJL064W | *yjl064wΔ* | a |
| YJL065C | *dls1Δ* | a |
| YJL066C | *mpm1Δ* | a |
| YJL067W | *yjl067wΔ* | a |
| YJL068C | *yjl068cΔ* | a |
| YJL069C | *utp18-ts* | b |
| YJL070C | *yjl070cΔ* | a |
| YJL071W | *arg2Δ* | a |
| YJL072C | *psf2-ts* | b |
| YJL073W | *jem1Δ* | a |
| YJL075C | *apq13Δ* | a |
| YJL077C | *ics3Δ* | a |
| YJL077W-B | *yjl077w-bΔ* | a |
| YJL078C | *pry3Δ* | a |
| YJL079C | *pry1Δ* | a |
| YJL080C | *scp160Δ* | a |
| YJL082W | *iml2Δ* | a |
| YJL083W | *tax4Δ* | a |
| YJL084C | *aly2Δ* | a |
| YJL087C | *trl1-ts* | b |
| YJL088W | *arg3Δ* | a |
| YJL088W | *arg3Δ* | a |
| YJL089W | *sip4Δ* | a |
| YJL092W | *srs2Δ* | a |
| YJL093C | *tok1Δ* | a |
| YJL094C | *kha1Δ* | a |
| YJL095W | *bck1Δ* | a |
| YJL096W | *mrpl49Δ* | a |
| YJL096W | *mrpl49Δ* | a |
| YJL097W | *phs1-ts* | b |
| YJL098W | *sap185Δ* | a |
| YJL099W | *chs6Δ* | a |
| YJL100W | *lsb6Δ* | a |
| YJL101C | *gsh1Δ* | a |
| YJL102W | *mef2Δ* | a |
| YJL103C | *gsm1Δ* | a |
| YJL105W | *set4Δ* | a |
| YJL106W | *ime2Δ* | a |
| YJL107C | *yjl107cΔ* | a |
| YJL108C | *prm10Δ* | a |
| YJL110C | *gzf3Δ* | a |
| YJL112W | *mdv1Δ* | a |
| YJL115W | *asf1Δ* | a |
| YJL116C | *nca3Δ* | a |
| YJL117W | *pho86Δ* | a |
| YJL118W | *yjl118wΔ* | a |
| YJL119C | *yjl119cΔ* | a |
| YJL120W | *yjl120wΔ* | a |
| YJL121C | *rpe1Δ* | a |
| YJL122W | *alb1Δ* | a |
| YJL123C | *mtc1Δ* | a |
| YJL124C | *lsm1Δ* | a |
| YJL126W | *nit2Δ* | a |
| YJL127C | *spt10Δ* | a |
| YJL127C-B | *yjl127c-bΔ* | a |
| YJL128C | *pbs2Δ* | a |
| YJL129C | *trk1Δ* | a |
| YJL129C | *trk1Δ* | a |
| YJL130C | *ura2Δ* | a |
| YJL131C | *aim23Δ* | a |
| YJL132W | *yjl132wΔ* | a |
| YJL132W | *yjl132wΔ* | a |
| YJL133W | *mrs3Δ* | a |
| YJL134W | *lcb3Δ* | a |
| YJL135W | *yjl135wΔ* | a |
| YJL136C | *rps21bΔ* | a |
| YJL136W-A | *yjl136w-aΔ* | a |
| YJL137C | *glg2Δ* | a |
| YJL138C | *tif2Δ* | a |
| YJL139C | *yur1Δ* | a |
| YJL139C | *yur1Δ* | a |
| YJL140W | *rpb4Δ* | a |
| YJL140W | *rpb4Δ* | a |
| YJL141C | *yak1Δ* | a |
| YJL142C | *irc9Δ* | a |
| YJL144W | *yjl144wΔ* | a |
| YJL145W | *sfh5Δ* | a |
| YJL146W | *ids2Δ* | a |
| YJL147C | *yjl147cΔ* | a |
| YJL148W | *rpa34Δ* | a |
| YJL149W | *yjl149wΔ* | a |
| YJL150W | *yjl150wΔ* | a |
| YJL151C | *sna3Δ* | a |
| YJL151C | *sna3Δ* | a |
| YJL152W | *yjl152wΔ* | a |
| YJL153C | *ino1Δ* | a |
| YJL154C | *vps35Δ* | a |
| YJL155C | *fbp26Δ* | a |
| YJL157C | *far1Δ* | a |
| YJL158C | *cis3Δ* | a |
| YJL159W | *hsp150Δ* | a |
| YJL160C | *yjl160cΔ* | a |
| YJL160C | *yjl160cΔ* | a |
| YJL161W | *fmp33Δ* | a |
| YJL161W | *fmp33Δ* | a |
| YJL162C | *jjj2Δ* | a |
| YJL163C | *yjl163cΔ* | a |
| YJL163C | *yjl163cΔ* | a |
| YJL164C | *tpk1Δ* | a |
| YJL165C | *hal5Δ* | a |
| YJL165C | *hal5Δ* | a |
| YJL166W | *qcr8Δ* | a |
| YJL168C | *set2Δ* | a |
| YJL169W | *yjl169wΔ* | a |
| YJL170C | *asg7Δ* | a |
| YJL171C | *yjl171cΔ* | a |
| YJL172W | *cps1Δ* | a |
| YJL172W | *cps1Δ* | a |
| YJL174W | *kre9-ts* | b |
| YJL175W | *yjl175wΔ* | a |
| YJL176C | *swi3Δ* | a |
| YJL177W | *rpl17bΔ* | a |
| YJL178C | *atg27Δ* | a |
| YJL179W | *pfd1Δ* | a |
| YJL180C | *atp12Δ* | a |
| YJL181W | *yjl181wΔ* | a |
| YJL182C | *yjl182cΔ* | a |
| YJL183W | *mnn11Δ* | a |
| YJL184W | *gon7Δ* | a |
| YJL185C | *yjl185cΔ* | a |
| YJL186W | *mnn5Δ* | a |
| YJL187C | *swe1Δ* | a |
| YJL188C | *bud19Δ* | a |
| YJL189W | *rpl39Δ* | a |
| YJL189W | *rpl39Δ* | a |
| YJL190C | *rps22aΔ* | a |
| YJL191W | *rps14bΔ* | a |
| YJL191W | *rps14bΔ* | a |
| YJL192C | *sop4Δ* | a |
| YJL193W | *yjl193wΔ* | a |
| YJL196C | *elo1Δ* | a |
| YJL196C | *elo1Δ* | a |
| YJL197W | *ubp12Δ* | a |
| YJL198W | *pho90Δ* | a |
| YJL199C | *mbb1Δ* | a |
| YJL200C | *aco2Δ* | a |
| YJL200C | *aco2Δ* | a |
| YJL201W | *ecm25Δ* | a |
| YJL203W | *prp21-1* | c |
| YJL204C | *rcy1Δ* | a |
| YJL206C | *yjl206cΔ* | a |
| YJL206C | *yjl206cΔ* | a |
| YJL206C-A | *yjl206c-aΔ* | a |
| YJL207C | *laa1Δ* | a |
| YJL208C | *nuc1Δ* | a |
| YJL209W | *cbp1Δ* | a |
| YJL210W | *pex2Δ* | a |
| YJL211C | *yjl211cΔ* | a |
| YJL212C | *opt1Δ* | a |
| YJL213W | *yjl213wΔ* | a |
| YJL214W | *hxt8Δ* | a |
| YJL215C | *yjl215cΔ* | a |
| YJL216C | *yjl216cΔ* | a |
| YJL217W | *ree1Δ* | a |
| YJL218W | *yjl218wΔ* | a |
| YJR001W | *avt1Δ* | a |
| YJR002W | *mpp10-ts* | b |
| YJR003C | *yjr003cΔ* | a |
| YJR004C | *sag1Δ* | a |
| YJR005C-A | *yjr005c-aΔ* | a |
| YJR005W | *apl1Δ* | a |
| YJR006W | *pol31-ts* | b |
| YJR007W | *sui2-ts* | b |
| YJR008W | *yjr008wΔ* | a |
| YJR009C | *tdh2Δ* | a |
| YJR010C-A | *spc1Δ* | a |
| YJR010W | *met3Δ* | a |
| YJR011C | *yjr011cΔ* | a |
| YJR014W | *tma22Δ* | a |
| YJR015W | *yjr015wΔ* | a |
| YJR018W | *yjr018wΔ* | a |
| YJR019C | *tes1Δ* | a |
| YJR020W | *yjr020wΔ* | a |
| YJR021C | *rec107Δ* | a |
| YJR024C | *mde1Δ* | a |
| YJR025C | *bna1Δ* | a |
| YJR026W | *yjr026wΔ* | a |
| YJR030C | *yjr030cΔ* | a |
| YJR031C | *gea1Δ* | a |
| YJR032W | *cpr7Δ* | a |
| YJR033C | *rav1Δ* | a |
| YJR034W | *pet191Δ* | a |
| YJR035W | *rad26Δ* | a |
| YJR036C | *hul4Δ* | a |
| YJR037W | *yjr037wΔ* | a |
| YJR038C | *yjr038cΔ* | a |
| YJR039W | *yjr039wΔ* | a |
| YJR040W | *gef1Δ* | a |
| YJR042W | *nup85-ts* | b |
| YJR043C | *pol32Δ* | a |
| YJR044C | *vps55Δ* | a |
| YJR047C | *anb1Δ* | a |
| YJR048W | *cyc1Δ* | a |
| YJR049C | *utr1Δ* | a |
| YJR050W | *isy1Δ* | a |
| YJR051W | *osm1Δ* | a |
| YJR052W | *rad7Δ* | a |
| YJR053W | *bfa1Δ* | a |
| YJR054W | *yjr054wΔ* | a |
| YJR055W | *hit1Δ* | a |
| YJR055W | *hit1Δ* | a |
| YJR055W | *hit1Δ* | a |
| YJR056C | *yjr056cΔ* | a |
| YJR058C | *aps2Δ* | a |
| YJR059W | *ptk2Δ* | a |
| YJR060W | *cbf1Δ* | a |
| YJR060W | *cbf1Δ* | a |
| YJR061W | *yjr061wΔ* | a |
| YJR062C | *nta1Δ* | a |
| YJR063W | *rpa12Δ* | a |
| YJR064W | *cct5-ts* | b |
| YJR066W | *tor1Δ* | a |
| YJR069C | *ham1Δ* | a |
| YJR070C | *lia1Δ* | a |
| YJR072C | *npa3-ts* | b |
| YJR073C | *opi3Δ* | a |
| YJR074W | *mog1Δ* | a |
| YJR075W | *hoc1Δ* | a |
| YJR077C | *mir1Δ* | a |
| YJR078W | *bna2Δ* | a |
| YJR079W | *yjr079wΔ* | a |
| YJR080C | *aim24Δ* | a |
| YJR082C | *eaf6Δ* | a |
| YJR083C | *acf4Δ* | a |
| YJR084W | *csn12Δ* | a |
| YJR085C | *yjr085cΔ* | a |
| YJR086W | *ste18Δ* | a |
| YJR087W | *yjr087wΔ* | a |
| YJR088C | *emc2Δ* | a |
| YJR090C | *grr1Δ* | a |
| YJR091C | *jsn1Δ* | a |
| YJR092W | *bud4Δ* | a |
| YJR093C | *fip1-ts* | b |
| YJR094C | *ime1Δ* | a |
| YJR094W-A | *rpl43bΔ* | a |
| YJR095W | *sfc1Δ* | a |
| YJR096W | *yjr096wΔ* | a |
| YJR097W | *jjj3Δ* | a |
| YJR098C | *yjr098cΔ* | a |
| YJR099W | *yuh1Δ* | a |
| YJR100C | *aim25Δ* | a |
| YJR101W | *rsm26Δ* | a |
| YJR102C | *vps25Δ* | a |
| YJR103W | *ura8Δ* | a |
| YJR104C | *sod1Δ* | a |
| YJR105W | *ado1Δ* | a |
| YJR106W | *ecm27Δ* | a |
| YJR107W | *yjr107wΔ* | a |
| YJR108W | *abm1Δ* | a |
| YJR109C | *cpa2Δ* | a |
| YJR110W | *ymr1Δ* | a |
| YJR111C | *yjr111cΔ* | a |
| YJR112W-A | *yjr112w-aΔ* | a |
| YJR113C | *rsm7Δ* | a |
| YJR114W | *yjr114wΔ* | a |
| YJR115W | *yjr115wΔ* | a |
| YJR116W | *yjr116wΔ* | a |
| YJR117W | *ste24Δ* | a |
| YJR118C | *ilm1Δ* | a |
| YJR119C | *jhd2Δ* | a |
| YJR120W | *yjr120wΔ* | a |
| YJR121W | *atp2Δ* | a |
| YJR122W | *iba57Δ* | a |
| YJR124C | *yjr124cΔ* | a |
| YJR125C | *ent3Δ* | a |
| YJR126C | *vps70Δ* | a |
| YJR127C | *rsf2Δ* | a |
| YJR128W | *yjr128wΔ* | a |
| YJR129C | *yjr129cΔ* | a |
| YJR130C | *str2Δ* | a |
| YJR131W | *mns1Δ* | a |
| YJR133W | *xpt1Δ* | a |
| YJR134C | *sgm1Δ* | a |
| YJR135C | *mcm22Δ* | a |
| YJR135W-A | *tim8Δ* | a |
| YJR137C | *ecm17Δ* | a |
| YJR139C | *hom6Δ* | a |
| YJR140C | *hir3Δ* | a |
| YJR141W | *yjr141w-ts* | b |
| YJR142W | *yjr142wΔ* | a |
| YJR143C | *pmt4Δ* | a |
| YJR144W | *mgm101Δ* | a |
| YJR145C | *rps4aΔ* | a |
| YJR146W | *yjr146wΔ* | a |
| YJR147W | *hms2Δ* | a |
| YJR148W | *bat2Δ* | a |
| YJR149W | *yjr149wΔ* | a |
| YJR150C | *dan1Δ* | a |
| YJR151C | *dan4Δ* | a |
| YJR151W-A | *yjr151w-aΔ* | a |
| YJR152W | *dal5Δ* | a |
| YJR153W | *pgu1Δ* | a |
| YJR154W | *yjr154wΔ* | a |
| YKL001C | *met14Δ* | a |
| YKL002W | *did4Δ* | a |
| YKL002W | *did4Δ* | a |
| YKL003C | *mrp17Δ* | a |
| YKL004W | *aur1-ts* | b |
| YKL005C | *bye1Δ* | a |
| YKL006W | *rpl14aΔ* | a |
| YKL007W | *cap1Δ* | a |
| YKL008C | *lac1Δ* | a |
| YKL009W | *mrt4Δ* | a |
| YKL010C | *ufd4Δ* | a |
| YKL011C | *cce1Δ* | a |
| YKL012W | *prp40-ts* | b |
| YKL013C | *arc19-ts* | b |
| YKL015W | *put3Δ* | a |
| YKL016C | *atp7Δ* | a |
| YKL017C | *hcs1Δ* | a |
| YKL018C-A | *ykl018c-aΔ* | a |
| YKL019W | *ram2-ts* | b |
| YKL020C | *spt23Δ* | a |
| YKL021C | *mak11-ts* | b |
| YKL023W | *ykl023wΔ* | a |
| YKL025C | *pan3Δ* | a |
| YKL026C | *gpx1Δ* | a |
| YKL027W | *ykl027wΔ* | a |
| YKL029C | *mae1Δ* | a |
| YKL030W | *ykl030wΔ* | a |
| YKL031W | *ykl031wΔ* | a |
| YKL032C | *ixr1Δ* | a |
| YKL033W | *tti1-ts* | b |
| YKL033W-A | *ykl033w-aΔ* | a |
| YKL033W-A | *ykl033w-aΔ* | a |
| YKL034W | *tul1Δ* | a |
| YKL035W | *ugp1-ts* | b |
| YKL037W | *aim26Δ* | a |
| YKL038W | *rgt1Δ* | a |
| YKL039W | *ptm1Δ* | a |
| YKL040C | *nfu1Δ* | a |
| YKL041W | *vps24Δ* | a |
| YKL043W | *phd1Δ* | a |
| YKL044W | *ykl044wΔ* | a |
| YKL046C | *dcw1Δ* | a |
| YKL047W | *ykl047wΔ* | a |
| YKL048C | *elm1Δ* | a |
| YKL050C | *ykl050cΔ* | a |
| YKL051W | *sfk1Δ* | a |
| YKL053C-A | *mdm35Δ* | a |
| YKL053W | *ykl053wΔ* | a |
| YKL054C | *def1Δ* | a |
| YKL055C | *oar1Δ* | a |
| YKL056C | *tma19Δ* | a |
| YKL057C | *nup120Δ* | a |
| YKL060C | *fba1-ts* | b |
| YKL061W | *ykl061wΔ* | a |
| YKL062W | *msn4Δ* | a |
| YKL063C | *ykl063cΔ* | a |
| YKL064W | *mnr2Δ* | a |
| YKL065C | *yet1Δ* | a |
| YKL065C | *yet1Δ* | a |
| YKL066W | *ykl066wΔ* | a |
| YKL067W | *ynk1Δ* | a |
| YKL068W | *nup100Δ* | a |
| YKL068W-A | *ykl068w-aΔ* | a |
| YKL069W | *ykl069wΔ* | a |
| YKL070W | *ykl070wΔ* | a |
| YKL071W | *ykl071wΔ* | a |
| YKL072W | *stb6Δ* | a |
| YKL073W | *lhs1Δ* | a |
| YKL074C | *mud2Δ* | a |
| YKL075C | *ykl075cΔ* | a |
| YKL076C | *psy1Δ* | a |
| YKL077W | *ykl077wΔ* | a |
| YKL078W | *dhr2-ts* | b |
| YKL079W | *smy1Δ* | a |
| YKL080W | *vma5Δ* | a |
| YKL081W | *tef4Δ* | a |
| YKL084W | *hot13Δ* | a |
| YKL085W | *mdh1Δ* | a |
| YKL086W | *srx1Δ* | a |
| YKL087C | *cyt2Δ* | a |
| YKL088W | *cab3-ts* | b |
| YKL090W | *cue2Δ* | a |
| YKL091C | *ykl091cΔ* | a |
| YKL092C | *bud2Δ* | a |
| YKL093W | *mbr1Δ* | a |
| YKL094W | *yju3Δ* | a |
| YKL095W | *yju2-ts* | b |
| YKL096C-B | *ykl096c-bΔ* | a |
| YKL096W | *cwp1Δ* | a |
| YKL096W-A | *cwp2Δ* | a |
| YKL097C | *ykl097cΔ* | a |
| YKL098W | *mtc2Δ* | a |
| YKL100C | *ykl100cΔ* | a |
| YKL101W | *hsl1Δ* | a |
| YKL102C | *ykl102cΔ* | a |
| YKL103C | *lap4Δ* | a |
| YKL105C | *ykl105cΔ* | a |
| YKL106C-A | *ykl106c-aΔ* | a |
| YKL106W | *aat1Δ* | a |
| YKL107W | *ykl107wΔ* | a |
| YKL109W | *hap4Δ* | a |
| YKL110C | *kti12Δ* | a |
| YKL113C | *rad27Δ* | a |
| YKL114C | *apn1Δ* | a |
| YKL115C | *ykl115cΔ* | a |
| YKL116C | *prr1Δ* | a |
| YKL117W | *sba1Δ* | a |
| YKL118W | *ykl118wΔ* | a |
| YKL119C | *vph2Δ* | a |
| YKL120W | *oac1Δ* | a |
| YKL121W | *dgr2Δ* | a |
| YKL123W | *ykl123wΔ* | a |
| YKL124W | *ssh4Δ* | a |
| YKL126W | *ypk1Δ* | a |
| YKL127W | *pgm1Δ* | a |
| YKL128C | *pmu1Δ* | a |
| YKL129C | *myo3Δ* | a |
| YKL130C | *she2Δ* | a |
| YKL131W | *ykl131wΔ* | a |
| YKL132C | *rma1Δ* | a |
| YKL133C | *ykl133cΔ* | a |
| YKL134C | *oct1Δ* | a |
| YKL135C | *apl2Δ* | a |
| YKL136W | *ykl136wΔ* | a |
| YKL137W | *cmc1Δ* | a |
| YKL137W | *cmc1Δ* | a |
| YKL138C | *mrpl31Δ* | a |
| YKL139W | *ctk1Δ* | a |
| YKL140W | *tgl1Δ* | a |
| YKL142W | *mrp8Δ* | a |
| YKL143W | *ltv1Δ* | a |
| YKL144C | *rpc25-ts* | b |
| YKL146W | *avt3Δ* | a |
| YKL147C | *ykl147cΔ* | a |
| YKL148C | *sdh1Δ* | a |
| YKL149C | *prp26-1* | c |
| YKL149C | *dbr1Δ* | a |
| YKL150W | *mcr1Δ* | a |
| YKL151C | *ykl151cΔ* | a |
| YKL155C | *rsm22Δ* | a |
| YKL156W | *rps27aΔ* | a |
| YKL157W | *ape2Δ* | a |
| YKL157W | *ape2Δ* | a |
| YKL158W | *ykl158wΔ* | a |
| YKL159C | *rcn1Δ* | a |
| YKL160W | *elf1Δ* | a |
| YKL161C | *ykl161cΔ* | a |
| YKL162C | *ykl162cΔ* | a |
| YKL162C-A | *ykl162c-aΔ* | a |
| YKL163W | *pir3Δ* | a |
| YKL164C | *pir1Δ* | a |
| YKL166C | *tpk3Δ* | a |
| YKL167C | *mrp49Δ* | a |
| YKL168C | *kkq8Δ* | a |
| YKL169C | *ykl169cΔ* | a |
| YKL170W | *mrpl38Δ* | a |
| YKL171W | *ykl171wΔ* | a |
| YKL173W | *snu114-12* | c |
| YKL174C | *tpo5Δ* | a |
| YKL175W | *zrt3Δ* | a |
| YKL176C | *lst4Δ* | a |
| YKL177W | *ykl177wΔ* | a |
| YKL178C | *ste3Δ* | a |
| YKL179C | *coy1Δ* | a |
| YKL181W | *prp1-1* | c |
| YKL183W | *lot5Δ* | a |
| YKL184W | *spe1Δ* | a |
| YKL185W | *ash1Δ* | a |
| YKL187C | *ykl187cΔ* | a |
| YKL188C | *pxa2Δ* | a |
| YKL190W | *cnb1Δ* | a |
| YKL191W | *dph2Δ* | a |
| YKL194C | *mst1Δ* | a |
| YKL195W | *mia40-ts* | b |
| YKL196C | *ykt6-ts* | b |
| YKL197C | *pex1Δ* | a |
| YKL198C | *ptk1Δ* | a |
| YKL198C | *ptk1Δ* | a |
| YKL199C | *ykl199cΔ* | a |
| YKL200C | *ykl200cΔ* | a |
| YKL201C | *mnn4Δ* | a |
| YKL201C | *mnn4Δ* | a |
| YKL202W | *ykl202wΔ* | a |
| YKL204W | *eap1Δ* | a |
| YKL205W | *los1Δ* | a |
| YKL206C | *add66Δ* | a |
| YKL207W | *aim27Δ* | a |
| YKL208W | *cbt1Δ* | a |
| YKL211C | *trp3Δ* | a |
| YKL212W | *sac1Δ* | a |
| YKL213C | *doa1Δ* | a |
| YKL214C | *yra2Δ* | a |
| YKL215C | *ykl215cΔ* | a |
| YKL216W | *ura1Δ* | a |
| YKL217W | *jen1Δ* | a |
| YKL218C | *sry1Δ* | a |
| YKL220C | *fre2Δ* | a |
| YKL221W | *mch2Δ* | a |
| YKL222C | *ykl222cΔ* | a |
| YKR001C | *vps1Δ* | a |
| YKR002W | *pap1-23* | c |
| YKR003W | *osh6Δ* | a |
| YKR004C-A | *ykr004c-aΔ* | a |
| YKR005C | *ykr005cΔ* | a |
| YKR006C | *mrpl13Δ* | a |
| YKR007W | *meh1Δ* | a |
| YKR009C | *fox2Δ* | a |
| YKR010C | *tof2Δ* | a |
| YKR011C | *ykr011cΔ* | a |
| YKR012C | *ykr012cΔ* | a |
| YKR013W | *pry2Δ* | a |
| YKR014C | *ypt52Δ* | a |
| YKR015C | *ykr015cΔ* | a |
| YKR016W | *aim28Δ* | a |
| YKR017C | *ykr017cΔ* | a |
| YKR018C | *ykr018cΔ* | a |
| YKR019C | *irs4Δ* | a |
| YKR020W | *vps51Δ* | a |
| YKR021W | *aly1Δ* | a |
| YKR022C | *ntr2-ts* | b |
| YKR023W | *ykr023wΔ* | a |
| YKR024C | *dbp7Δ* | a |
| YKR025W | *rpc37-ts* | b |
| YKR026C | *gcn3Δ* | a |
| YKR027W | *bch2Δ* | a |
| YKR028W | *sap190Δ* | a |
| YKR029C | *set3Δ* | a |
| YKR030W | *gmh1Δ* | a |
| YKR031C | *spo14Δ* | a |
| YKR032W | *ykr032wΔ* | a |
| YKR033C | *ykr033cΔ* | a |
| YKR034W | *dal80Δ* | a |
| YKR035C | *opi8Δ* | a |
| YKR035W-A | *did2Δ* | a |
| YKR036C | *caf4Δ* | a |
| YKR039W | *gap1Δ* | a |
| YKR040C | *ykr040cΔ* | a |
| YKR041W | *ykr041wΔ* | a |
| YKR042W | *uth1Δ* | a |
| YKR043C | *ykr043cΔ* | a |
| YKR044W | *uip5Δ* | a |
| YKR045C | *ykr045cΔ* | a |
| YKR046C | *pet10Δ* | a |
| YKR047W | *ykr047wΔ* | a |
| YKR048C | *nap1Δ* | a |
| YKR049C | *fmp46Δ* | a |
| YKR050W | *trk2Δ* | a |
| YKR051W | *ykr051wΔ* | a |
| YKR052C | *mrs4Δ* | a |
| YKR053C | *ysr3Δ* | a |
| YKR054C | *dyn1Δ* | a |
| YKR054C | *dyn1Δ* | a |
| YKR055W | *rho4Δ* | a |
| YKR056W | *trm2Δ* | a |
| YKR057W | *rps21aΔ* | a |
| YKR058W | *glg1Δ* | a |
| YKR059W | *tif1Δ* | a |
| YKR060W | *utp30Δ* | a |
| YKR061W | *ktr2Δ* | a |
| YKR063C | *las1-ts* | b |
| YKR064W | *oaf3Δ* | a |
| YKR065C | *pam17Δ* | a |
| YKR066C | *ccp1Δ* | a |
| YKR067W | *gpt2Δ* | a |
| YKR068C | *bet3-ts* | b |
| YKR069W | *met1Δ* | a |
| YKR070W | *ykr070wΔ* | a |
| YKR071C | *dre2-ts* | b |
| YKR072C | *sis2Δ* | a |
| YKR073C | *ykr073cΔ* | a |
| YKR074W | *aim29Δ* | a |
| YKR075C | *ykr075cΔ* | a |
| YKR076W | *ecm4Δ* | a |
| YKR077W | *msa2Δ* | a |
| YKR078W | *ykr078wΔ* | a |
| YKR080W | *mtd1Δ* | a |
| YKR081C | *rpf2-ts* | b |
| YKR082W | *nup133Δ* | a |
| YKR084C | *hbs1Δ* | a |
| YKR085C | *mrpl20Δ* | a |
| YKR086W | *prp16-2* | c |
| YKR087C | *oma1Δ* | a |
| YKR088C | *tvp38Δ* | a |
| YKR089C | *tgl4Δ* | a |
| YKR090W | *pxl1Δ* | a |
| YKR091W | *srl3Δ* | a |
| YKR091W | *srl3Δ* | a |
| YKR092C | *srp40Δ* | a |
| YKR093W | *ptr2Δ* | a |
| YKR094C | *rpl40bΔ* | a |
| YKR095W | *mlp1Δ* | a |
| YKR096W | *ykr096wΔ* | a |
| YKR097W | *pck1Δ* | a |
| YKR098C | *ubp11Δ* | a |
| YKR099C-A | *ykr099c-aΔ* | a |
| YKR099W | *bas1Δ* | a |
| YKR100C | *skg1Δ* | a |
| YKR100C | *skg1Δ* | a |
| YKR101W | *sir1Δ* | a |
| YKR102W | *flo10Δ* | a |
| YKR103W | *nft1Δ* | a |
| YKR104W | *ykr104wΔ* | a |
| YKR105C | *vba5Δ* | a |
| YKR106W | *ykr106wΔ* | a |
| YLL001W | *dnm1Δ* | a |
| YLL002W | *rtt109Δ* | a |
| YLL005C | *spo75Δ* | a |
| YLL006W | *mmm1Δ* | a |
| YLL006W-A | *yll006w-aΔ* | a |
| YLL007C | *yll007cΔ* | a |
| YLL008W | *drs1-ts* | b |
| YLL009C | *cox17Δ* | a |
| YLL010C | *psr1Δ* | a |
| YLL011W | *sof1-ts* | b |
| YLL012W | *yeh1Δ* | a |
| YLL013C | *puf3Δ* | a |
| YLL014W | *emc6Δ* | a |
| YLL015W | *bpt1Δ* | a |
| YLL016W | *yll016wΔ* | a |
| YLL017W | *yll017wΔ* | a |
| YLL018C-A | *cox19Δ* | a |
| YLL019C | *kns1Δ* | a |
| YLL020C | *yll020cΔ* | a |
| YLL021W | *spa2Δ* | a |
| YLL023C | *yll023cΔ* | a |
| YLL024C | *ssa2Δ* | a |
| YLL025W | *pau17Δ* | a |
| YLL026W | *hsp104Δ* | a |
| YLL027W | *isa1Δ* | a |
| YLL028W | *tpo1Δ* | a |
| YLL029W | *fra1Δ* | a |
| YLL030C | *rrt7Δ* | a |
| YLL032C | *yll032cΔ* | a |
| YLL033W | *irc19Δ* | a |
| YLL035W | *grc3-ts* | b |
| YLL036C | *prp19-1* | c |
| YLL038C | *ent4Δ* | a |
| YLL039C | *ubi4Δ* | a |
| YLL040C | *vps13Δ* | a |
| YLL041C | *sdh2Δ* | a |
| YLL042C | *atg10Δ* | a |
| YLL043W | *fps1Δ* | a |
| YLL044W | *yll044wΔ* | a |
| YLL045C | *rpl8bΔ* | a |
| YLL046C | *rnp1Δ* | a |
| YLL047W | *yll047wΔ* | a |
| YLL048C | *ybt1Δ* | a |
| YLL049W | *ldb18Δ* | a |
| YLL051C | *fre6Δ* | a |
| YLL052C | *aqy2Δ* | a |
| YLL053C | *yll053cΔ* | a |
| YLL054C | *yll054cΔ* | a |
| YLL055W | *yct1Δ* | a |
| YLL056C | *yll056cΔ* | a |
| YLL057C | *jlp1Δ* | a |
| YLL058W | *yll058wΔ* | a |
| YLL059C | *yll059cΔ* | a |
| YLL060C | *gtt2Δ* | a |
| YLL061W | *mmp1Δ* | a |
| YLL062C | *mht1Δ* | a |
| YLL063C | *ayt1Δ* | a |
| YLR001C | *ylr001cΔ* | a |
| YLR002C | *noc3-ts* | b |
| YLR003C | *cms1Δ* | a |
| YLR004C | *thi73Δ* | a |
| YLR005W | *ssl1-ts* | b |
| YLR006C | *ssk1Δ* | a |
| YLR007W | *nse1-ts* | b |
| YLR011W | *lot6Δ* | a |
| YLR012C | *ylr012cΔ* | a |
| YLR013W | *gat3Δ* | a |
| YLR014C | *ppr1Δ* | a |
| YLR015W | *bre2Δ* | a |
| YLR016C | *pml1Δ* | a |
| YLR017W | *meu1Δ* | a |
| YLR018C | *pom34Δ* | a |
| YLR019W | *psr2Δ* | a |
| YLR020C | *yeh2Δ* | a |
| YLR021W | *irc25Δ* | a |
| YLR022C | *sdo1-ts* | b |
| YLR023C | *izh3Δ* | a |
| YLR024C | *ubr2Δ* | a |
| YLR025W | *snf7Δ* | a |
| YLR027C | *aat2Δ* | a |
| YLR028C | *ade16Δ* | a |
| YLR029C | *rpl15a-ts* | b |
| YLR030W | *ylr030wΔ* | a |
| YLR031W | *ylr031wΔ* | a |
| YLR032W | *rad5Δ* | a |
| YLR033W | *rsc58-ts* | b |
| YLR034C | *smf3Δ* | a |
| YLR035C | *mlh2Δ* | a |
| YLR036C | *ylr036cΔ* | a |
| YLR037C | *dan2Δ* | a |
| YLR038C | *cox12Δ* | a |
| YLR039C | *ric1Δ* | a |
| YLR040C | *ylr040cΔ* | a |
| YLR041W | *ylr041wΔ* | a |
| YLR042C | *ylr042cΔ* | a |
| YLR043C | *trx1Δ* | a |
| YLR044C | *pdc1Δ* | a |
| YLR046C | *ylr046cΔ* | a |
| YLR047C | *fre8Δ* | a |
| YLR048W | *rps0bΔ* | a |
| YLR049C | *ylr049cΔ* | a |
| YLR050C | *ylr050cΔ* | a |
| YLR052W | *ies3Δ* | a |
| YLR053C | *ylr053cΔ* | a |
| YLR054C | *osw2Δ* | a |
| YLR054C | *osw2Δ* | a |
| YLR055C | *spt8Δ* | a |
| YLR056W | *erg3Δ* | a |
| YLR057W | *ylr057wΔ* | a |
| YLR058C | *shm2Δ* | a |
| YLR059C | *rex2Δ* | a |
| YLR060W | *frs1-ts* | b |
| YLR060W | *frs1-ts* | b |
| YLR061W | *rpl22aΔ* | a |
| YLR062C | *bud28Δ* | a |
| YLR063W | *ylr063wΔ* | a |
| YLR064W | *ylr064wΔ* | a |
| YLR065C | *ylr065cΔ* | a |
| YLR067C | *pet309Δ* | a |
| YLR068W | *fyv7Δ* | a |
| YLR069C | *mef1Δ* | a |
| YLR070C | *xyl2Δ* | a |
| YLR072W | *ylr072wΔ* | a |
| YLR073C | *ylr073cΔ* | a |
| YLR074C | *bud20Δ* | a |
| YLR077W | *fmp25Δ* | a |
| YLR079W | *sic1Δ* | a |
| YLR080W | *emp46Δ* | a |
| YLR081W | *gal2Δ* | a |
| YLR082C | *srl2Δ* | a |
| YLR083C | *emp70Δ* | a |
| YLR084C | *rax2Δ* | a |
| YLR084C | *rax2Δ* | a |
| YLR085C | *arp6Δ* | a |
| YLR087C | *csf1Δ* | a |
| YLR089C | *alt1Δ* | a |
| YLR090W | *xdj1Δ* | a |
| YLR091W | *gep5Δ* | a |
| YLR092W | *sul2Δ* | a |
| YLR093C | *nyv1Δ* | a |
| YLR094C | *gis3Δ* | a |
| YLR095C | *ioc2Δ* | a |
| YLR096W | *kin2Δ* | a |
| YLR097C | *hrt3Δ* | a |
| YLR098C | *cha4Δ* | a |
| YLR099C | *ict1Δ* | a |
| YLR100W | *erg27-ts* | b |
| YLR102C | *apc9Δ* | a |
| YLR104W | *ylr104wΔ* | a |
| YLR107W | *rex3Δ* | a |
| YLR108C | *ylr108cΔ* | a |
| YLR109W | *ahp1Δ* | a |
| YLR110C | *ccw12Δ* | a |
| YLR111W | *ylr111wΔ* | a |
| YLR112W | *ylr112wΔ* | a |
| YLR113W | *hog1Δ* | a |
| YLR114C | *avl9Δ* | a |
| YLR115W | *cft2-ts* | b |
| YLR116W | *msl5-ts* | b |
| YLR118C | *ylr118cΔ* | a |
| YLR118C | *ylr118cΔ* | a |
| YLR119W | *srn2Δ* | a |
| YLR120C | *yps1Δ* | a |
| YLR121C | *yps3Δ* | a |
| YLR122C | *ylr122cΔ* | a |
| YLR123C | *ylr123cΔ* | a |
| YLR124W | *ylr124wΔ* | a |
| YLR125W | *ylr125wΔ* | a |
| YLR125W | *ylr125wΔ* | a |
| YLR126C | *ylr126cΔ* | a |
| YLR128W | *dcn1Δ* | a |
| YLR129W | *dip2-ts* | b |
| YLR130C | *zrt2Δ* | a |
| YLR131C | *ace2Δ* | a |
| YLR132C | *ylr132c-ts* | b |
| YLR133W | *cki1Δ* | a |
| YLR134W | *pdc5Δ* | a |
| YLR135W | *slx4Δ* | a |
| YLR136C | *tis11Δ* | a |
| YLR137W | *ylr137wΔ* | a |
| YLR138W | *nha1Δ* | a |
| YLR139C | *sls1Δ* | a |
| YLR141W | *rrn5-ts* | b |
| YLR142W | *put1Δ* | a |
| YLR143W | *ylr143wΔ* | a |
| YLR144C | *acf2Δ* | a |
| YLR145W | *rmp1-ts* | b |
| YLR146C | *spe4Δ* | a |
| YLR147C | *smd3-ts* | b |
| YLR148W | *pep3Δ* | a |
| YLR149C | *ylr149cΔ* | a |
| YLR150W | *stm1Δ* | a |
| YLR151C | *pcd1Δ* | a |
| YLR152C | *ylr152cΔ* | a |
| YLR153C | *acs2-ts* | b |
| YLR154C | *rnh203Δ* | a |
| YLR164W | *ylr164wΔ* | a |
| YLR165C | *pus5Δ* | a |
| YLR168C | *ylr168cΔ* | a |
| YLR169W | *ylr169wΔ* | a |
| YLR170C | *aps1Δ* | a |
| YLR171W | *ylr171wΔ* | a |
| YLR172C | *dph5Δ* | a |
| YLR173W | *ylr173wΔ* | a |
| YLR174W | *idp2Δ* | a |
| YLR175W | *cbf5-ts* | b |
| YLR176C | *rfx1Δ* | a |
| YLR177W | *ylr177wΔ* | a |
| YLR178C | *tfs1Δ* | a |
| YLR179C | *ylr179cΔ* | a |
| YLR180W | *sam1Δ* | a |
| YLR181C | *vta1Δ* | a |
| YLR182W | *swi6Δ* | a |
| YLR183C | *tos4Δ* | a |
| YLR184W | *ylr184wΔ* | a |
| YLR185W | *rpl37aΔ* | a |
| YLR186W | *emg1-ts* | b |
| YLR187W | *skg3Δ* | a |
| YLR188W | *mdl1Δ* | a |
| YLR189C | *atg26Δ* | a |
| YLR190W | *mmr1Δ* | a |
| YLR191W | *pex13Δ* | a |
| YLR192C | *hcr1Δ* | a |
| YLR193C | *ups1Δ* | a |
| YLR194C | *ylr194cΔ* | a |
| YLR194C | *ylr194cΔ* | a |
| YLR196W | *pwp1-ts* | b |
| YLR197W | *nop56-ts* | b |
| YLR199C | *pba1Δ* | a |
| YLR200W | *yke2Δ* | a |
| YLR201C | *coq9Δ* | a |
| YLR202C | *ylr202cΔ* | a |
| YLR203C | *mss51Δ* | a |
| YLR204W | *qri5Δ* | a |
| YLR205C | *hmx1Δ* | a |
| YLR206W | *ent2Δ* | a |
| YLR207W | *hrd3Δ* | a |
| YLR209C | *pnp1Δ* | a |
| YLR210W | *clb4Δ* | a |
| YLR211C | *ylr211cΔ* | a |
| YLR211C | *ylr211cΔ* | a |
| YLR213C | *crr1Δ* | a |
| YLR214W | *fre1Δ* | a |
| YLR216C | *cpr6Δ* | a |
| YLR217W | *ylr217wΔ* | a |
| YLR218C | *ylr218cΔ* | a |
| YLR219W | *msc3Δ* | a |
| YLR220W | *ccc1Δ* | a |
| YLR221C | *rsa3Δ* | a |
| YLR222C | *utp13-ts* | b |
| YLR224W | *ylr224wΔ* | a |
| YLR225C | *ylr225cΔ* | a |
| YLR226W | *bur2Δ* | a |
| YLR227C | *ady4Δ* | a |
| YLR228C | *ecm22Δ* | a |
| YLR231C | *bna5Δ* | a |
| YLR232W | *ylr232wΔ* | a |
| YLR233C | *est1Δ* | a |
| YLR234W | *top3Δ* | a |
| YLR235C | *ylr235cΔ* | a |
| YLR236C | *ylr236cΔ* | a |
| YLR237W | *thi7Δ* | a |
| YLR238W | *far10Δ* | a |
| YLR239C | *lip2Δ* | a |
| YLR240W | *vps34Δ* | a |
| YLR241W | *ylr241wΔ* | a |
| YLR242C | *arv1Δ* | a |
| YLR244C | *map1Δ* | a |
| YLR246W | *erf2Δ* | a |
| YLR247C | *irc20Δ* | a |
| YLR248W | *rck2Δ* | a |
| YLR250W | *ssp120Δ* | a |
| YLR251W | *sym1Δ* | a |
| YLR251W | *sym1Δ* | a |
| YLR252W | *ylr252wΔ* | a |
| YLR253W | *ylr253wΔ* | a |
| YLR254C | *ndl1Δ* | a |
| YLR255C | *ylr255cΔ* | a |
| YLR257W | *ylr257wΔ* | a |
| YLR258W | *gsy2Δ* | a |
| YLR260W | *lcb5Δ* | a |
| YLR261C | *vps63Δ* | a |
| YLR262C | *ypt6Δ* | a |
| YLR262C-A | *tma7Δ* | a |
| YLR263W | *red1Δ* | a |
| YLR264C-A | *ylr264c-aΔ* | a |
| YLR264W | *rps28bΔ* | a |
| YLR265C | *nej1Δ* | a |
| YLR266C | *pdr8Δ* | a |
| YLR267W | *bop2Δ* | a |
| YLR268W | *sec22Δ* | a |
| YLR269C | *ylr269cΔ* | a |
| YLR270W | *dcs1Δ* | a |
| YLR271W | *ylr271wΔ* | a |
| YLR273C | *pig1Δ* | a |
| YLR275W | *smd2-ts* | b |
| YLR276C | *dbp9-ts* | b |
| YLR277C | *ysh1-ts* | b |
| YLR278C | *ylr278cΔ* | a |
| YLR279W | *ylr279wΔ* | a |
| YLR280C | *ylr280cΔ* | a |
| YLR281C | *ylr281cΔ* | a |
| YLR282C | *ylr282cΔ* | a |
| YLR283W | *ylr283wΔ* | a |
| YLR284C | *eci1Δ* | a |
| YLR285C-A | *ylr285c-aΔ* | a |
| YLR285W | *nnt1Δ* | a |
| YLR286C | *cts1Δ* | a |
| YLR287-A | *ylr287-aΔ* | a |
| YLR287C | *ylr287cΔ* | a |
| YLR288C | *mec3Δ* | a |
| YLR289W | *guf1Δ* | a |
| YLR290C | *ylr290cΔ* | a |
| YLR291C | *gcd7-ts* | b |
| YLR292C | *sec72Δ* | a |
| YLR294C | *ylr294cΔ* | a |
| YLR295C | *atp14Δ* | a |
| YLR296W | *ylr296wΔ* | a |
| YLR297W | *ylr297wΔ* | a |
| YLR299W | *ecm38Δ* | a |
| YLR300W | *exg1Δ* | a |
| YLR303W | *met17Δ* | a |
| YLR304C | *aco1Δ* | a |
| YLR306W | *ubc12Δ* | a |
| YLR307C-A | *ylr307c-aΔ* | a |
| YLR307W | *cda1Δ* | a |
| YLR308W | *cda2Δ* | a |
| YLR309C | *imh1Δ* | a |
| YLR311C | *ylr311cΔ* | a |
| YLR312C | *ylr312cΔ* | a |
| YLR312C-B | *ylr312c-bΔ* | a |
| YLR312W-A | *mrpl15Δ* | a |
| YLR313C | *sph1Δ* | a |
| YLR315W | *nkp2Δ* | a |
| YLR316C | *tad3-ts* | b |
| YLR317W | *ylr317w-ts* | b |
| YLR318W | *est2Δ* | a |
| YLR319C | *bud6Δ* | a |
| YLR320W | *mms22Δ* | a |
| YLR322W | *vps65Δ* | a |
| YLR324W | *pex30Δ* | a |
| YLR325C | *rpl38Δ* | a |
| YLR326W | *ylr326wΔ* | a |
| YLR327C | *tma10Δ* | a |
| YLR328W | *nma1Δ* | a |
| YLR329W | *rec102Δ* | a |
| YLR329W | *rec102Δ* | a |
| YLR330W | *chs5Δ* | a |
| YLR331C | *jip3Δ* | a |
| YLR332W | *mid2Δ* | a |
| YLR332W | *mid2Δ* | a |
| YLR333C | *rps25bΔ* | a |
| YLR334C | *ylr334cΔ* | a |
| YLR335W | *nup2Δ* | a |
| YLR337C | *vrp1Δ* | a |
| YLR338W | *opi9Δ* | a |
| YLR340W | *rpp0-ts* | b |
| YLR341W | *spo77Δ* | a |
| YLR342W | *fks1Δ* | a |
| YLR342W-A | *ylr342w-aΔ* | a |
| YLR343W | *gas2Δ* | a |
| YLR344W | *rpl26aΔ* | a |
| YLR345W | *ylr345wΔ* | a |
| YLR346C | *ylr346cΔ* | a |
| YLR348C | *dic1Δ* | a |
| YLR349W | *ylr349wΔ* | a |
| YLR350W | *orm2Δ* | a |
| YLR351C | *nit3Δ* | a |
| YLR352W | *ylr352wΔ* | a |
| YLR353W | *bud8Δ* | a |
| YLR354C | *tal1Δ* | a |
| YLR356W | *ylr356wΔ* | a |
| YLR357W | *rsc2Δ* | a |
| YLR358C | *ylr358cΔ* | a |
| YLR360W | *vps38Δ* | a |
| YLR361C | *dcr2Δ* | a |
| YLR361C-A | *ylr361c-aΔ* | a |
| YLR362W | *ste11Δ* | a |
| YLR363C | *nmd4Δ* | a |
| YLR363W-A | *ylr363w-aΔ* | a |
| YLR364W | *grx8Δ* | a |
| YLR365W | *ylr365wΔ* | a |
| YLR366W | *ylr366wΔ* | a |
| YLR367W | *rps22bΔ* | a |
| YLR368W | *mdm30Δ* | a |
| YLR369W | *ssq1Δ* | a |
| YLR370C | *arc18Δ* | a |
| YLR371W | *rom2Δ* | a |
| YLR371W | *rom2Δ* | a |
| YLR372W | *sur4Δ* | a |
| YLR373C | *vid22Δ* | a |
| YLR374C | *ylr374cΔ* | a |
| YLR375W | *stp3Δ* | a |
| YLR376C | *psy3Δ* | a |
| YLR377C | *fbp1Δ* | a |
| YLR380W | *csr1Δ* | a |
| YLR381W | *ctf3Δ* | a |
| YLR382C | *nam2Δ* | a |
| YLR384C | *iki3Δ* | a |
| YLR385C | *swc7Δ* | a |
| YLR386W | *vac14Δ* | a |
| YLR387C | *reh1Δ* | a |
| YLR388W | *rps29aΔ* | a |
| YLR389C | *ste23Δ* | a |
| YLR390W | *ecm19Δ* | a |
| YLR390W-A | *ccw14Δ* | a |
| YLR391W | *ylr391wΔ* | a |
| YLR392C | *ylr392cΔ* | a |
| YLR393W | *atp10Δ* | a |
| YLR394W | *cst9Δ* | a |
| YLR395C | *cox8Δ* | a |
| YLR396C | *vps33Δ* | a |
| YLR398C | *ski2Δ* | a |
| YLR399C | *bdf1Δ* | a |
| YLR400W | *ylr400wΔ* | a |
| YLR401C | *dus3Δ* | a |
| YLR402W | *ylr402wΔ* | a |
| YLR403W | *sfp1Δ* | a |
| YLR404W | *fld1Δ* | a |
| YLR405W | *dus4Δ* | a |
| YLR406C | *rpl31bΔ* | a |
| YLR406C-A | *ylr406c-aΔ* | a |
| YLR407W | *ylr407wΔ* | a |
| YLR408C | *ylr408cΔ* | a |
| YLR410W | *vip1Δ* | a |
| YLR412C-A | *ylr412c-aΔ* | a |
| YLR412W | *ber1Δ* | a |
| YLR413W | *ylr413wΔ* | a |
| YLR414C | *ylr414cΔ* | a |
| YLR415C | *ylr415cΔ* | a |
| YLR416C | *ylr416cΔ* | a |
| YLR417W | *vps36Δ* | a |
| YLR418C | *cdc73Δ* | a |
| YLR419W | *ylr419wΔ* | a |
| YLR420W | *ura4Δ* | a |
| YLR421C | *rpn13Δ* | a |
| YLR422W | *ylr422wΔ* | a |
| YLR423C | *atg17Δ* | a |
| YLR424W | *spp382-ts* | b |
| YLR425W | *tus1Δ* | a |
| YLR426W | *ylr426wΔ* | a |
| YLR427W | *mag2Δ* | a |
| YLR428C | *ylr428cΔ* | a |
| YLR429W | *crn1Δ* | a |
| YLR431C | *atg23Δ* | a |
| YLR432W | *imd3Δ* | a |
| YLR433C | *cna1Δ* | a |
| YLR434C | *ylr434cΔ* | a |
| YLR435W | *tsr2Δ* | a |
| YLR436C | *ecm30Δ* | a |
| YLR437C | *dif1Δ* | a |
| YLR438W | *car2Δ* | a |
| YLR439W | *mrpl4Δ* | a |
| YLR440C | *sec39-ts* | b |
| YLR441C | *rps1aΔ* | a |
| YLR442C | *sir3Δ* | a |
| YLR443W | *ecm7Δ* | a |
| YLR444C | *ylr444cΔ* | a |
| YLR445W | *ylr445wΔ* | a |
| YLR445W | *ylr445wΔ* | a |
| YLR446W | *ylr446wΔ* | a |
| YLR447C | *vma6Δ* | a |
| YLR448W | *rpl6bΔ* | a |
| YLR449W | *fpr4Δ* | a |
| YLR450W | *hmg2Δ* | a |
| YLR451W | *leu3Δ* | a |
| YLR452C | *sst2Δ* | a |
| YLR453C | *rif2Δ* | a |
| YLR454W | *fmp27Δ* | a |
| YLR455W | *ylr455wΔ* | a |
| YLR456W | *ylr456wΔ* | a |
| YLR460C | *ylr460cΔ* | a |
| YLR461W | *pau4Δ* | a |
| YML001W | *ypt7Δ* | a |
| YML002W | *yml002wΔ* | a |
| YML003W | *yml003wΔ* | a |
| YML004C | *glo1Δ* | a |
| YML005W | *trm12Δ* | a |
| YML006C | *gis4Δ* | a |
| YML007C-A | *yml007c-aΔ* | a |
| YML007W | *yap1Δ* | a |
| YML008C | *erg6Δ* | a |
| YML009c | *yml009cΔ* | a |
| YML009C | *mrpl39Δ* | a |
| YML010C-B | *yml010c-bΔ* | a |
| YML010W-A | *yml010w-aΔ* | a |
| YML011C | *rad33Δ* | a |
| YML012W | *erv25Δ* | a |
| YML013C-A | *yml013c-aΔ* | a |
| YML013W | *ubx2Δ* | a |
| YML014W | *trm9Δ* | a |
| YML016C | *ppz1Δ* | a |
| YML017W | *psp2Δ* | a |
| YML018C | *yml018cΔ* | a |
| YML019W | *ost6Δ* | a |
| YML020W | *yml020wΔ* | a |
| YML021C | *ung1Δ* | a |
| YML022W | *apt1Δ* | a |
| YML023C | *nse5-ts* | b |
| YML024W | *rps17aΔ* | a |
| YML026C | *rps18bΔ* | a |
| YML027W | *yox1Δ* | a |
| YML028W | *tsa1Δ* | a |
| YML029W | *usa1Δ* | a |
| YML030W | *aim31Δ* | a |
| YML032C | *rad52Δ* | a |
| YML033W | *yml033wΔ* | a |
| YML034W | *src1Δ* | a |
| YML034W | *src1Δ* | a |
| YML035C | *amd1Δ* | a |
| YML035C | *amd1Δ* | a |
| YML035C-A | *yml035c-aΔ* | a |
| YML036W | *cgi121Δ* | a |
| YML037C | *yml037cΔ* | a |
| YML038C | *ymd8Δ* | a |
| YML041C | *vps71Δ* | a |
| YML042W | *cat2Δ* | a |
| YML047C | *prm6Δ* | a |
| YML048W | *gsf2Δ* | a |
| YML048W-A | *yml048w-aΔ* | a |
| YML050W | *aim32Δ* | a |
| YML051W | *gal80Δ* | a |
| YML052W | *sur7Δ* | a |
| YML053C | *yml053cΔ* | a |
| YML054C | *cyb2Δ* | a |
| YML054C-A | *yml054c-aΔ* | a |
| YML055W | *spc2Δ* | a |
| YML056C | *imd4Δ* | a |
| YML057W | *cmp2Δ* | a |
| YML058C-A | *yml058c-aΔ* | a |
| YML058W | *sml1Δ* | a |
| YML058W-A | *hug1Δ* | a |
| YML059C | *nte1Δ* | a |
| YML060W | *ogg1Δ* | a |
| YML061C | *pif1Δ* | a |
| YML062C | *mft1Δ* | a |
| YML063W | *rps1bΔ* | a |
| YML065W | *orc1-ts* | b |
| YML066C | *sma2Δ* | a |
| YML067C | *erv41Δ* | a |
| YML068W | *itt1Δ* | a |
| YML070W | *dak1Δ* | a |
| YML071C | *cog8Δ* | a |
| YML072C | *tcb3Δ* | a |
| YML073C | *rpl6aΔ* | a |
| YML074C | *fpr3Δ* | a |
| YML075C | *hmg1Δ* | a |
| YML076C | *war1Δ* | a |
| YML077W | *bet5-ts* | b |
| YML078W | *cpr3Δ* | a |
| YML079W | *yml079wΔ* | a |
| YML080W | *dus1Δ* | a |
| YML081C-A | *atp18Δ* | a |
| YML081W | *yml081wΔ* | a |
| YML082W | *yml082wΔ* | a |
| YML083C | *yml083cΔ* | a |
| YML084W | *yml084wΔ* | a |
| YML086C | *alo1Δ* | a |
| YML086C | *alo1Δ* | a |
| YML087C | *aim33Δ* | a |
| YML088W | *ufo1Δ* | a |
| YML089C | *yml089cΔ* | a |
| YML090W | *yml090wΔ* | a |
| YML092C | *pre8-ts* | b |
| YML094W | *gim5Δ* | a |
| YML095C | *rad10Δ* | a |
| YML095C-A | *yml095c-aΔ* | a |
| YML096W | *yml096wΔ* | a |
| YML097C | *vps9Δ* | a |
| YML099C | *arg81Δ* | a |
| YML100W | *tsl1Δ* | a |
| YML100W-A | *yml100w-aΔ* | a |
| YML101C | *cue4Δ* | a |
| YML102C-A | *yml102c-aΔ* | a |
| YML102W | *cac2Δ* | a |
| YML103C | *nup188Δ* | a |
| YML104C | *mdm1Δ* | a |
| YML104C | *mdm1Δ* | a |
| YML106W | *ura5Δ* | a |
| YML107C | *pml39Δ* | a |
| YML108W | *yml108wΔ* | a |
| YML109W | *zds2Δ* | a |
| YML110C | *coq5Δ* | a |
| YML111W | *bul2Δ* | a |
| YML112W | *ctk3Δ* | a |
| YML113W | *dat1Δ* | a |
| YML115C | *van1Δ* | a |
| YML116W | *atr1Δ* | a |
| YML117W | *nab6Δ* | a |
| YML117W-A | *yml117w-aΔ* | a |
| YML118W | *ngl3Δ* | a |
| YML119W | *yml119wΔ* | a |
| YML120C | *ndi1Δ* | a |
| YML121W | *gtr1Δ* | a |
| YML122C | *yml122cΔ* | a |
| YML123C | *pho84Δ* | a |
| YML124C | *tub3Δ* | a |
| YML125C | *pga3-ts* | b |
| YML126C | *erg13-ts* | b |
| YML127W | *rsc9-ts* | b |
| YML128C | *msc1Δ* | a |
| YML129C | *cox14Δ* | a |
| YML131W | *yml131wΔ* | a |
| YMR001C-A | *ymr001c-aΔ* | a |
| YMR002W | *mic17Δ* | a |
| YMR003W | *aim34Δ* | a |
| YMR004W | *mvp1Δ* | a |
| YMR006C | *plb2Δ* | a |
| YMR007W | *ymr007wΔ* | a |
| YMR008C | *plb1Δ* | a |
| YMR009W | *adi1Δ* | a |
| YMR010W | *ymr010wΔ* | a |
| YMR011W | *hxt2Δ* | a |
| YMR012W | *clu1Δ* | a |
| YMR013W-A | *ymr013w-aΔ* | a |
| YMR014W | *bud22Δ* | a |
| YMR015C | *erg5Δ* | a |
| YMR016C | *sok2Δ* | a |
| YMR017W | *spo20Δ* | a |
| YMR018W | *ymr018wΔ* | a |
| YMR019W | *stb4Δ* | a |
| YMR020W | *fms1Δ* | a |
| YMR021C | *mac1Δ* | a |
| YMR022W | *ubc7Δ* | a |
| YMR023C | *mss1Δ* | a |
| YMR024W | *mrpl3Δ* | a |
| YMR025W | *csi1Δ* | a |
| YMR026C | *pex12Δ* | a |
| YMR027W | *ymr027wΔ* | a |
| YMR029C | *far8Δ* | a |
| YMR030W | *rsf1Δ* | a |
| YMR031C | *ymr031cΔ* | a |
| YMR031W-A | *ymr031w-aΔ* | a |
| YMR032W | *hof1Δ* | a |
| YMR032W | *hof1Δ* | a |
| YMR034C | *ymr034cΔ* | a |
| YMR035W | *imp2Δ* | a |
| YMR036C | *mih1Δ* | a |
| YMR037C | *msn2Δ* | a |
| YMR038C | *ccs1Δ* | a |
| YMR039C | *sub1Δ* | a |
| YMR040W | *yet2Δ* | a |
| YMR041C | *ara2Δ* | a |
| YMR042W | *arg80Δ* | a |
| YMR043W | *mcm1-ts* | b |
| YMR044W | *ioc4Δ* | a |
| YMR048W | *csm3Δ* | a |
| YMR049C | *erb1-ts* | b |
| YMR052C-A | *ymr052c-aΔ* | a |
| YMR052W | *far3Δ* | a |
| YMR053C | *stb2Δ* | a |
| YMR054W | *stv1Δ* | a |
| YMR055C | *bub2Δ* | a |
| YMR056C | *aac1Δ* | a |
| YMR057C | *ymr057cΔ* | a |
| YMR058W | *fet3Δ* | a |
| YMR060C | *sam37Δ* | a |
| YMR061W | *rna14-64* | c |
| YMR061W | *rna14-ts* | b |
| YMR062C | *ecm40Δ* | a |
| YMR063W | *rim9Δ* | a |
| YMR064W | *aep1Δ* | a |
| YMR065W | *kar5Δ* | a |
| YMR066W | *sov1Δ* | a |
| YMR067C | *ubx4Δ* | a |
| YMR068W | *avo2Δ* | a |
| YMR069W | *nat4Δ* | a |
| YMR070W | *mot3Δ* | a |
| YMR071C | *tvp18Δ* | a |
| YMR072W | *abf2Δ* | a |
| YMR073C | *irc21Δ* | a |
| YMR074C | *ymr074cΔ* | a |
| YMR075C-A | *ymr075c-aΔ* | a |
| YMR075W | *rco1Δ* | a |
| YMR077C | *vps20Δ* | a |
| YMR078C | *ctf18Δ* | a |
| YMR080C | *nam7Δ* | a |
| YMR081C | *isf1Δ* | a |
| YMR082C | *ymr082cΔ* | a |
| YMR083W | *adh3Δ* | a |
| YMR084W | *ymr084wΔ* | a |
| YMR085W | *ymr085wΔ* | a |
| YMR086C-A | *ymr086c-aΔ* | a |
| YMR086W | *ymr086wΔ* | a |
| YMR087W | *ymr087wΔ* | a |
| YMR088C | *vba1Δ* | a |
| YMR089C | *yta12Δ* | a |
| YMR090W | *ymr090wΔ* | a |
| YMR091C | *npl6Δ* | a |
| YMR092C | *aip1Δ* | a |
| YMR093W | *utp15-ts* | b |
| YMR095C | *sno1Δ* | a |
| YMR096W | *snz1Δ* | a |
| YMR097C | *mtg1Δ* | a |
| YMR098C | *atp25Δ* | a |
| YMR099C | *ymr099cΔ* | a |
| YMR100W | *mub1Δ* | a |
| YMR101C | *srt1Δ* | a |
| YMR102C | *ymr102cΔ* | a |
| YMR103C | *ymr103cΔ* | a |
| YMR104C | *ypk2Δ* | a |
| YMR105C | *pgm2Δ* | a |
| YMR105W-A | *ymr105w-aΔ* | a |
| YMR106C | *yku80Δ* | a |
| YMR107W | *spg4Δ* | a |
| YMR109W | *myo5Δ* | a |
| YMR110C | *hfd1Δ* | a |
| YMR111C | *ymr111cΔ* | a |
| YMR114C | *ymr114cΔ* | a |
| YMR115W | *mgr3Δ* | a |
| YMR116C | *asc1Δ* | a |
| YMR118C | *ymr118cΔ* | a |
| YMR119W | *asi1Δ* | a |
| YMR119W-A | *ymr119w-aΔ* | a |
| YMR120C | *ade17Δ* | a |
| YMR121C | *rpl15bΔ* | a |
| YMR122C | *ymr122cΔ* | a |
| YMR123W | *pkr1Δ* | a |
| YMR124W | *ymr124wΔ* | a |
| YMR125W | *sto1Δ* | a |
| YMR126C | *dlt1Δ* | a |
| YMR127C | *sas2Δ* | a |
| YMR129W | *pom152Δ* | a |
| YMR130W | *ymr130wΔ* | a |
| YMR132C | *jlp2Δ* | a |
| YMR133W | *rec114Δ* | a |
| YMR134W | *ymr134w-ts* | b |
| YMR135C | *gid8Δ* | a |
| YMR135W-A | *ymr135w-aΔ* | a |
| YMR136W | *gat2Δ* | a |
| YMR137C | *pso2Δ* | a |
| YMR138W | *cin4Δ* | a |
| YMR139W | *rim11Δ* | a |
| YMR140W | *sip5Δ* | a |
| YMR141C | *ymr141cΔ* | a |
| YMR142C | *rpl13bΔ* | a |
| YMR143W | *rps16aΔ* | a |
| YMR143W | *rps16aΔ* | a |
| YMR144W | *ymr144wΔ* | a |
| YMR145C | *nde1Δ* | a |
| YMR146C | *tif34-ts* | b |
| YMR147W | *ymr147wΔ* | a |
| YMR148W | *ymr148wΔ* | a |
| YMR149W | *swp1-ts* | b |
| YMR150C | *imp1Δ* | a |
| YMR151W | *yim2Δ* | a |
| YMR152W | *yim1Δ* | a |
| YMR153C-A | *ymr153c-aΔ* | a |
| YMR153W | *nup53Δ* | a |
| YMR154C | *rim13Δ* | a |
| YMR155W | *ymr155wΔ* | a |
| YMR156C | *tpp1Δ* | a |
| YMR157C | *aim36Δ* | a |
| YMR158C-B | *ymr158c-bΔ* | a |
| YMR158W | *mrps8Δ* | a |
| YMR158W-A | *ymr158w-aΔ* | a |
| YMR159C | *atg16Δ* | a |
| YMR160W | *ymr160wΔ* | a |
| YMR161W | *hlj1Δ* | a |
| YMR162C | *dnf3Δ* | a |
| YMR163C | *inp2Δ* | a |
| YMR164C | *mss11Δ* | a |
| YMR166C | *ymr166cΔ* | a |
| YMR167W | *mlh1Δ* | a |
| YMR169C | *ald3Δ* | a |
| YMR169C | *ald3Δ* | a |
| YMR170C | *ald2Δ* | a |
| YMR171C | *ear1Δ* | a |
| YMR172C-A | *ymr172c-aΔ* | a |
| YMR172W | *hot1Δ* | a |
| YMR173W | *ddr48Δ* | a |
| YMR173W-A | *ymr173w-aΔ* | a |
| YMR174C | *pai3Δ* | a |
| YMR174C | *pai3Δ* | a |
| YMR175W | *sip18Δ* | a |
| YMR175W | *sip18Δ* | a |
| YMR175W-A | *ymr175w-aΔ* | a |
| YMR176W | *ecm5Δ* | a |
| YMR177W | *mmt1Δ* | a |
| YMR178W | *ymr178wΔ* | a |
| YMR179W | *spt21Δ* | a |
| YMR180C | *ctl1Δ* | a |
| YMR181C | *ymr181cΔ* | a |
| YMR182C | *rgm1Δ* | a |
| YMR182W-A | *ymr182w-aΔ* | a |
| YMR183C | *sso2Δ* | a |
| YMR184W | *add37Δ* | a |
| YMR186W | *hsc82Δ* | a |
| YMR187C | *ymr187cΔ* | a |
| YMR188C | *mrps17Δ* | a |
| YMR189W | *gcv2Δ* | a |
| YMR190C | *sgs1Δ* | a |
| YMR191W | *spg5Δ* | a |
| YMR191W | *spg5Δ* | a |
| YMR192W | *gyl1Δ* | a |
| YMR193C-A | *ymr193c-aΔ* | a |
| YMR193W | *mrpl24Δ* | a |
| YMR194C-A | *ymr194c-aΔ* | a |
| YMR194C-B | *ymr194c-bΔ* | a |
| YMR194W | *rpl36aΔ* | a |
| YMR195W | *icy1Δ* | a |
| YMR196W | *ymr196wΔ* | a |
| YMR198W | *cik1Δ* | a |
| YMR198W | *cik1Δ* | a |
| YMR199W | *cln1Δ* | a |
| YMR200W | *rot1-ts* | b |
| YMR201C | *rad14Δ* | a |
| YMR202W | *erg2Δ* | a |
| YMR202W | *erg2Δ* | a |
| YMR204C | *inp1Δ* | a |
| YMR205C | *pfk2Δ* | a |
| YMR206W | *ymr206wΔ* | a |
| YMR207C | *hfa1Δ* | a |
| YMR208W | *erg12-ts* | b |
| YMR209C | *ymr209cΔ* | a |
| YMR210W | *ymr210wΔ* | a |
| YMR211W | *dml1-ts* | b |
| YMR214W | *scj1Δ* | a |
| YMR215W | *gas3Δ* | a |
| YMR216C | *sky1Δ* | a |
| YMR218C | *trs130-ts* | b |
| YMR219W | *esc1Δ* | a |
| YMR221C | *ymr221cΔ* | a |
| YMR222C | *fsh2Δ* | a |
| YMR223W | *ubp8Δ* | a |
| YMR224C | *mre11Δ* | a |
| YMR225C | *mrpl44Δ* | a |
| YMR226C | *ymr226cΔ* | a |
| YMR228W | *mtf1Δ* | a |
| YMR230W | *rps10bΔ* | a |
| YMR230W-A | *ymr230w-aΔ* | a |
| YMR231W | *pep5Δ* | a |
| YMR232W | *fus2Δ* | a |
| YMR233W | *tri1Δ* | a |
| YMR234W | *rnh1Δ* | a |
| YMR237W | *bch1Δ* | a |
| YMR238W | *dfg5Δ* | a |
| YMR241W | *yhm2Δ* | a |
| YMR242C | *rpl20aΔ* | a |
| YMR242W-A | *ymr242w-aΔ* | a |
| YMR243C | *zrc1Δ* | a |
| YMR244C-A | *ymr244c-aΔ* | a |
| YMR244W | *ymr244wΔ* | a |
| YMR245W | *ymr245wΔ* | a |
| YMR246W | *faa4Δ* | a |
| YMR247C | *rkr1Δ* | a |
| YMR247W-A | *ymr247w-aΔ* | a |
| YMR250W | *gad1Δ* | a |
| YMR251W | *gto3Δ* | a |
| YMR251W-A | *hor7Δ* | a |
| YMR252C | *ymr252cΔ* | a |
| YMR253C | *ymr253cΔ* | a |
| YMR254C | *ymr254cΔ* | a |
| YMR255W | *gfd1Δ* | a |
| YMR256C | *cox7Δ* | a |
| YMR257C | *pet111Δ* | a |
| YMR258C | *ymr258cΔ* | a |
| YMR259C | *ymr259cΔ* | a |
| YMR260C | *tif11-ts* | b |
| YMR261C | *tps3Δ* | a |
| YMR262W | *ymr262wΔ* | a |
| YMR263W | *sap30Δ* | a |
| YMR264W | *cue1Δ* | a |
| YMR265C | *ymr265cΔ* | a |
| YMR266W | *rsn1Δ* | a |
| YMR267W | *ppa2Δ* | a |
| YMR268C | *prp24-1* | c |
| YMR269W | *tma23Δ* | a |
| YMR269W | *tma23Δ* | a |
| YMR271C | *ura10Δ* | a |
| YMR272C | *scs7Δ* | a |
| YMR272W-B | *ymr272w-bΔ* | a |
| YMR273C | *zds1Δ* | a |
| YMR274C | *rce1Δ* | a |
| YMR275C | *bul1Δ* | a |
| YMR276W | *dsk2Δ* | a |
| YMR278W | *pgm3Δ* | a |
| YMR279C | *ymr279cΔ* | a |
| YMR280C | *cat8Δ* | a |
| YMR281W | *gpi12-ts* | b |
| YMR282C | *aep2Δ* | a |
| YMR283C | *rit1Δ* | a |
| YMR284W | *yku70Δ* | a |
| YMR285C | *ngl2Δ* | a |
| YMR286W | *mrpl33Δ* | a |
| YMR287C | *dss1Δ* | a |
| YMR288W | *hsh155-ts* | b |
| YMR289W | *abz2Δ* | a |
| YMR290C | *has1-ts* | b |
| YMR291W | *ymr291wΔ* | a |
| YMR292W | *got1Δ* | a |
| YMR293C | *her2Δ* | a |
| YMR294W | *jnm1Δ* | a |
| YMR294W-A | *ymr294w-aΔ* | a |
| YMR295C | *ymr295cΔ* | a |
| YMR297W | *prc1Δ* | a |
| YMR298W | *lip1-ts* | b |
| YMR299C | *dyn3Δ* | a |
| YMR300C | *ade4Δ* | a |
| YMR302C | *yme2Δ* | a |
| YMR303C | *adh2Δ* | a |
| YMR304C-A | *ymr304c-aΔ* | a |
| YMR304W | *ubp15Δ* | a |
| YMR305C | *scw10Δ* | a |
| YMR306C-A | *ymr306c-aΔ* | a |
| YMR306W | *fks3Δ* | a |
| YMR307W | *gas1Δ* | a |
| YMR310C | *ymr310cΔ* | a |
| YMR311C | *glc8Δ* | a |
| YMR312W | *elp6Δ* | a |
| YMR313C | *tgl3Δ* | a |
| YMR314W | *pre5-ts* | b |
| YMR315W | *ymr315wΔ* | a |
| YMR315W-A | *ymr315w-aΔ* | a |
| YMR316C-A | *ymr316c-aΔ* | a |
| YMR316C-B | *ymr316c-bΔ* | a |
| YMR316W | *dia1Δ* | a |
| YMR317W | *ymr317wΔ* | a |
| YMR318C | *adh6Δ* | a |
| YMR319C | *fet4Δ* | a |
| YMR320W | *ymr320wΔ* | a |
| YMR322C | *sno4Δ* | a |
| YMR326C | *ymr326cΔ* | a |
| YNL001W | *dom34Δ* | a |
| YNL002C | *rlp7-ts* | b |
| YNL003C | *pet8Δ* | a |
| YNL004W | *hrb1Δ* | a |
| YNL005C | *mrp7Δ* | a |
| YNL007C | *sis1-ts* | b |
| YNL008C | *asi3Δ* | a |
| YNL009W | *idp3Δ* | a |
| YNL010W | *ynl010wΔ* | a |
| YNL011C | *ynl011cΔ* | a |
| YNL012W | *spo1Δ* | a |
| YNL013C | *ynl013cΔ* | a |
| YNL014W | *hef3Δ* | a |
| YNL015W | *pbi2Δ* | a |
| YNL016W | *pub1Δ* | a |
| YNL020C | *ark1Δ* | a |
| YNL021W | *hda1Δ* | a |
| YNL022C | *ynl022cΔ* | a |
| YNL023C | *fap1Δ* | a |
| YNL024C | *ynl024cΔ* | a |
| YNL025C | *ssn8Δ* | a |
| YNL027W | *crz1Δ* | a |
| YNL028W | *ynl028wΔ* | a |
| YNL029C | *ktr5Δ* | a |
| YNL030W | *hhf2Δ* | a |
| YNL031C | *hht2Δ* | a |
| YNL032W | *siw14Δ* | a |
| YNL034W | *ynl034wΔ* | a |
| YNL035C | *ynl035cΔ* | a |
| YNL037C | *idh1Δ* | a |
| YNL038W | *gpi15-ts* | b |
| YNL040W | *ynl040wΔ* | a |
| YNL041C | *cog6Δ* | a |
| YNL042W-B | *ynl042w-bΔ* | a |
| YNL043C | *ynl043cΔ* | a |
| YNL044W | *yip3Δ* | a |
| YNL045W | *lap2Δ* | a |
| YNL046W | *ynl046wΔ* | a |
| YNL047C | *slm2Δ* | a |
| YNL049C | *sfb2Δ* | a |
| YNL050C | *ynl050cΔ* | a |
| YNL051W | *cog5Δ* | a |
| YNL052W | *cox5aΔ* | a |
| YNL053W | *msg5Δ* | a |
| YNL054W | *vac7Δ* | a |
| YNL055C | *por1Δ* | a |
| YNL056W | *oca2Δ* | a |
| YNL057W | *ynl057wΔ* | a |
| YNL058C | *ynl058cΔ* | a |
| YNL059C | *arp5Δ* | a |
| YNL063W | *mtq1Δ* | a |
| YNL064C | *ydj1Δ* | a |
| YNL065W | *aqr1Δ* | a |
| YNL066W | *sun4Δ* | a |
| YNL067W | *rpl9bΔ* | a |
| YNL067W-B | *ynl067w-bΔ* | a |
| YNL068C | *fkh2Δ* | a |
| YNL069C | *rpl16bΔ* | a |
| YNL069C | *rpl16bΔ* | a |
| YNL070W | *tom7Δ* | a |
| YNL071W | *lat1Δ* | a |
| YNL072W | *rnh201Δ* | a |
| YNL073W | *msk1Δ* | a |
| YNL074C | *mlf3Δ* | a |
| YNL076W | *mks1Δ* | a |
| YNL077W | *apj1Δ* | a |
| YNL078W | *nis1Δ* | a |
| YNL079C | *tpm1Δ* | a |
| YNL080C | *eos1Δ* | a |
| YNL081C | *sws2Δ* | a |
| YNL082W | *pms1Δ* | a |
| YNL083W | *sal1Δ* | a |
| YNL084C | *end3Δ* | a |
| YNL085W | *mkt1Δ* | a |
| YNL086W | *ynl086wΔ* | a |
| YNL087W | *tcb2Δ* | a |
| YNL089C | *ynl089cΔ* | a |
| YNL089C | *ynl089cΔ* | a |
| YNL090W | *rho2Δ* | a |
| YNL090W | *rho2Δ* | a |
| YNL091W | *nst1Δ* | a |
| YNL092W | *ynl092wΔ* | a |
| YNL093W | *ypt53Δ* | a |
| YNL094W | *app1Δ* | a |
| YNL095C | *ynl095cΔ* | a |
| YNL096C | *rps7bΔ* | a |
| YNL096C | *rps7bΔ* | a |
| YNL097C | *pho23Δ* | a |
| YNL097C-A | *ynl097c-aΔ* | a |
| YNL098C | *ras2Δ* | a |
| YNL099C | *oca1Δ* | a |
| YNL100W | *aim37Δ* | a |
| YNL101W | *avt4Δ* | a |
| YNL104C | *leu4Δ* | a |
| YNL105W | *rrt16Δ* | a |
| YNL106C | *inp52Δ* | a |
| YNL107W | *yaf9Δ* | a |
| YNL108C | *ynl108cΔ* | a |
| YNL109W | *ynl109wΔ* | a |
| YNL110C | *nop15-ts* | b |
| YNL111C | *cyb5Δ* | a |
| YNL115C | *ynl115cΔ* | a |
| YNL116W | *dma2Δ* | a |
| YNL117W | *mls1Δ* | a |
| YNL119W | *ncs2Δ* | a |
| YNL120C | *ynl120cΔ* | a |
| YNL121C | *tom70Δ* | a |
| YNL122C | *ynl122cΔ* | a |
| YNL123W | *nma111Δ* | a |
| YNL125C | *esbp6Δ* | a |
| YNL126W | *spc98-ts* | b |
| YNL127W | *far11Δ* | a |
| YNL128W | *tep1Δ* | a |
| YNL129W | *nrk1Δ* | a |
| YNL130C | *cpt1Δ* | a |
| YNL130C-A | *dgr1Δ* | a |
| YNL131W | *tom22-ts* | b |
| YNL132W | *kre33-ts* | b |
| YNL133C | *fyv6Δ* | a |
| YNL134C | *ynl134cΔ* | a |
| YNL135C | *fpr1Δ* | a |
| YNL136W | *eaf7Δ* | a |
| YNL138W | *srv2Δ* | a |
| YNL139C | *tho2Δ* | a |
| YNL140C | *ynl140cΔ* | a |
| YNL141W | *aah1Δ* | a |
| YNL142W | *mep2Δ* | a |
| YNL143C | *ynl143cΔ* | a |
| YNL144C | *ynl144cΔ* | a |
| YNL145W | *mfa2Δ* | a |
| YNL146C-A | *ynl146c-aΔ* | a |
| YNL146W | *ynl146wΔ* | a |
| YNL147W | *lsm7Δ* | a |
| YNL147W | *lsm7Δ* | a |
| YNL148C | *alf1Δ* | a |
| YNL152W | *inn1-ts* | b |
| YNL153C | *gim3Δ* | a |
| YNL154C | *yck2Δ* | a |
| YNL155W | *ynl155wΔ* | a |
| YNL156C | *nsg2Δ* | a |
| YNL157W | *ynl157wΔ* | a |
| YNL158W | *pga1-ts* | b |
| YNL159C | *asi2Δ* | a |
| YNL160W | *ygp1Δ* | a |
| YNL162W | *rpl42aΔ* | a |
| YNL162W | *rpl42aΔ* | a |
| YNL162W-A | *ynl162w-aΔ* | a |
| YNL164C | *ibd2Δ* | a |
| YNL165W | *ynl165wΔ* | a |
| YNL166C | *bni5Δ* | a |
| YNL167C | *sko1Δ* | a |
| YNL168C | *fmp41Δ* | a |
| YNL169C | *psd1Δ* | a |
| YNL170W | *ynl170wΔ* | a |
| YNL171C | *ynl171cΔ* | a |
| YNL173C | *mdg1Δ* | a |
| YNL175C | *nop13Δ* | a |
| YNL176C | *ynl176cΔ* | a |
| YNL177C | *mrpl22Δ* | a |
| YNL179C | *ynl179cΔ* | a |
| YNL181W | *ynl181w-ts* | b |
| YNL182C | *ipi3-ts* | b |
| YNL183C | *npr1Δ* | a |
| YNL184C | *ynl184cΔ* | a |
| YNL187W | *swt21Δ* | a |
| YNL189W | *srp1-ts* | b |
| YNL190W | *ynl190wΔ* | a |
| YNL191W | *dug3Δ* | a |
| YNL192W | *chs1Δ* | a |
| YNL193W | *ynl193wΔ* | a |
| YNL194C | *ynl194cΔ* | a |
| YNL195C | *ynl195cΔ* | a |
| YNL196C | *slz1Δ* | a |
| YNL197C | *whi3Δ* | a |
| YNL198C | *ynl198cΔ* | a |
| YNL199C | *gcr2Δ* | a |
| YNL200C | *ynl200cΔ* | a |
| YNL201C | *psy2Δ* | a |
| YNL202W | *sps19Δ* | a |
| YNL203C | *ynl203cΔ* | a |
| YNL204C | *sps18Δ* | a |
| YNL205C | *ynl205cΔ* | a |
| YNL206C | *rtt106Δ* | a |
| YNL208W | *ynl208wΔ* | a |
| YNL209W | *ssb2Δ* | a |
| YNL211C | *ynl211cΔ* | a |
| YNL212W | *vid27Δ* | a |
| YNL213C | *ynl213cΔ* | a |
| YNL214W | *pex17Δ* | a |
| YNL215W | *ies2Δ* | a |
| YNL217W | *ynl217wΔ* | a |
| YNL218W | *mgs1Δ* | a |
| YNL219C | *alg9Δ* | a |
| YNL220W | *ade12Δ* | a |
| YNL223W | *atg4Δ* | a |
| YNL224C | *sqs1Δ* | a |
| YNL225C | *cnm67Δ* | a |
| YNL226W | *ynl226wΔ* | a |
| YNL227C | *jjj1Δ* | a |
| YNL228W | *ynl228wΔ* | a |
| YNL229C | *ure2Δ* | a |
| YNL230C | *ela1Δ* | a |
| YNL231C | *pdr16Δ* | a |
| YNL232W | *csl4-ts* | b |
| YNL233W | *bni4Δ* | a |
| YNL234W | *ynl234wΔ* | a |
| YNL235C | *ynl235cΔ* | a |
| YNL236W | *sin4Δ* | a |
| YNL237W | *ytp1Δ* | a |
| YNL238W | *kex2Δ* | a |
| YNL239W | *lap3Δ* | a |
| YNL241C | *zwf1Δ* | a |
| YNL242W | *atg2Δ* | a |
| YNL244C | *sui1-ts* | b |
| YNL246W | *vps75Δ* | a |
| YNL247W | *ynl247w-ts* | b |
| YNL248C | *rpa49Δ* | a |
| YNL249C | *mpa43Δ* | a |
| YNL250W | *rad50Δ* | a |
| YNL252C | *mrpl17Δ* | a |
| YNL253W | *tex1Δ* | a |
| YNL254C | *rtc4Δ* | a |
| YNL255C | *gis2Δ* | a |
| YNL256W | *fol1-ts* | b |
| YNL257C | *sip3Δ* | a |
| YNL259C | *atx1Δ* | a |
| YNL260C | *ynl260c-ts* | b |
| YNL264C | *pdr17Δ* | a |
| YNL265C | *ist1Δ* | a |
| YNL266W | *ynl266wΔ* | a |
| YNL268W | *lyp1Δ* | a |
| YNL268W | *lyp1Δ* | a |
| YNL269W | *bsc4Δ* | a |
| YNL270C | *alp1Δ* | a |
| YNL271C | *bni1Δ* | a |
| YNL273W | *tof1Δ* | a |
| YNL274C | *gor1Δ* | a |
| YNL275W | *bor1Δ* | a |
| YNL276C | *ynl276cΔ* | a |
| YNL277W | *met2Δ* | a |
| YNL277W-A | *ynl277w-aΔ* | a |
| YNL278W | *caf120Δ* | a |
| YNL279W | *prm1Δ* | a |
| YNL280C | *erg24Δ* | a |
| YNL280C | *erg24Δ* | a |
| YNL281W | *hch1Δ* | a |
| YNL282W | *pop3-ts* | b |
| YNL283C | *wsc2Δ* | a |
| YNL284C | *mrpl10Δ* | a |
| YNL285W | *ynl285wΔ* | a |
| YNL286W | *cus2Δ* | a |
| YNL288W | *caf40Δ* | a |
| YNL289W | *pcl1Δ* | a |
| YNL291C | *mid1Δ* | a |
| YNL292W | *pus4Δ* | a |
| YNL293W | *msb3Δ* | a |
| YNL294C | *rim21Δ* | a |
| YNL295W | *ynl295wΔ* | a |
| YNL296W | *ynl296wΔ* | a |
| YNL297C | *mon2Δ* | a |
| YNL298W | *cla4Δ* | a |
| YNL299W | *trf5Δ* | a |
| YNL300W | *tos6Δ* | a |
| YNL301C | *rpl18bΔ* | a |
| YNL302C | *rps19bΔ* | a |
| YNL303W | *ynl303wΔ* | a |
| YNL304W | *ypt11Δ* | a |
| YNL305C | *ynl305cΔ* | a |
| YNL307C | *mck1Δ* | a |
| YNL309W | *stb1Δ* | a |
| YNL310C | *zim17-ts* | b |
| YNL311C | *ynl311cΔ* | a |
| YNL312W | *rfa2-ts* | b |
| YNL313C | *ynl313c-ts* | b |
| YNL314W | *dal82Δ* | a |
| YNL315C | *atp11Δ* | a |
| YNL315C | *atp11Δ* | a |
| YNL316C | *pha2Δ* | a |
| YNL318C | *hxt14Δ* | a |
| YNL319W | *ynl319wΔ* | a |
| YNL320W | *ynl320wΔ* | a |
| YNL321W | *vnx1Δ* | a |
| YNL322C | *kre1Δ* | a |
| YNL323W | *lem3Δ* | a |
| YNL324W | *ynl324wΔ* | a |
| YNL325C | *fig4Δ* | a |
| YNL326C | *pfa3Δ* | a |
| YNL327W | *egt2Δ* | a |
| YNL328C | *mdj2Δ* | a |
| YNL329C | *pex6Δ* | a |
| YNL330C | *rpd3Δ* | a |
| YNL332W | *thi12Δ* | a |
| YNL333W | *snz2Δ* | a |
| YNL334C | *sno2Δ* | a |
| YNL335W | *ddi3Δ* | a |
| YNL336W | *cos1Δ* | a |
| YNL338W | *ynl338wΔ* | a |
| YNL339C | *yrf1-6Δ* | a |
| YNR001C | *cit1Δ* | a |
| YNR002C | *ato2Δ* | a |
| YNR004W | *ynr004wΔ* | a |
| YNR005C | *ynr005cΔ* | a |
| YNR006W | *vps27Δ* | a |
| YNR007C | *atg3Δ* | a |
| YNR008W | *lro1Δ* | a |
| YNR009W | *nrm1Δ* | a |
| YNR010W | *cse2Δ* | a |
| YNR011C | *prp2-1* | c |
| YNR012W | *urk1Δ* | a |
| YNR013C | *pho91Δ* | a |
| YNR014W | *ynr014wΔ* | a |
| YNR015W | *smm1Δ* | a |
| YNR017W | *tim23-ts* | b |
| YNR018W | *aim38Δ* | a |
| YNR019W | *are2Δ* | a |
| YNR020C | *atp23Δ* | a |
| YNR021W | *ynr021wΔ* | a |
| YNR022C | *mrpl50Δ* | a |
| YNR024W | *mpp6Δ* | a |
| YNR025C | *ynr025cΔ* | a |
| YNR027W | *bud17Δ* | a |
| YNR028W | *cpr8Δ* | a |
| YNR029C | *ynr029cΔ* | a |
| YNR030W | *alg12Δ* | a |
| YNR031C | *ssk2Δ* | a |
| YNR032C-A | *hub1Δ* | a |
| YNR032W | *ppg1Δ* | a |
| YNR033W | *abz1Δ* | a |
| YNR034W | *sol1Δ* | a |
| YNR036C | *mrps12Δ* | a |
| YNR037C | *rsm19Δ* | a |
| YNR038W | *dbp6-ts* | b |
| YNR039C | *zrg17Δ* | a |
| YNR040W | *ynr040wΔ* | a |
| YNR041C | *coq2Δ* | a |
| YNR042W | *ynr042wΔ* | a |
| YNR043W | *mvd1-ts* | b |
| YNR045W | *pet494Δ* | a |
| YNR047W | *fpk1Δ* | a |
| YNR048W | *ynr048wΔ* | a |
| YNR049C | *mso1Δ* | a |
| YNR050C | *lys9Δ* | a |
| YNR051C | *bre5Δ* | a |
| YNR052C | *pop2Δ* | a |
| YNR052C | *pop2Δ* | a |
| YNR055C | *hol1Δ* | a |
| YNR056C | *bio5Δ* | a |
| YNR057C | *bio4Δ* | a |
| YNR058W | *bio3Δ* | a |
| YNR059W | *mnt4Δ* | a |
| YNR060W | *fre4Δ* | a |
| YNR061C | *ynr061cΔ* | a |
| YNR062C | *ynr062cΔ* | a |
| YNR063W | *ynr063wΔ* | a |
| YNR064C | *ynr064cΔ* | a |
| YNR065C | *ynr065cΔ* | a |
| YNR066C | *ynr066cΔ* | a |
| YNR067C | *dse4Δ* | a |
| YNR068C | *ynr068cΔ* | a |
| YNR069C | *bsc5Δ* | a |
| YNR070W | *ynr070wΔ* | a |
| YNR071C | *ynr071cΔ* | a |
| YNR072W | *hxt17Δ* | a |
| YNR073C | *ynr073cΔ* | a |
| YNR074C | *aif1Δ* | a |
| YNR075W | *cos10Δ* | a |
| YOL001W | *pho80Δ* | a |
| YOL002C | *izh2Δ* | a |
| YOL003C | *pfa4Δ* | a |
| YOL004W | *sin3Δ* | a |
| YOL006C | *top1Δ* | a |
| YOL007C | *csi2Δ* | a |
| YOL008W | *coq10Δ* | a |
| YOL009C | *mdm12Δ* | a |
| YOL011W | *plb3Δ* | a |
| YOL012C | *htz1Δ* | a |
| YOL013C | *hrd1Δ* | a |
| YOL013W-A | *yol013w-aΔ* | a |
| YOL013W-B | *yol013w-bΔ* | a |
| YOL014W | *yol014wΔ* | a |
| YOL015W | *irc10Δ* | a |
| YOL016C | *cmk2Δ* | a |
| YOL017W | *esc8Δ* | a |
| YOL018C | *tlg2Δ* | a |
| YOL019W | *yol019wΔ* | a |
| YOL019W-A | *yol019w-aΔ* | a |
| YOL020W | *tat2Δ* | a |
| YOL021C | *dis3-ts* | b |
| YOL022C | *yol022c-ts* | b |
| YOL023W | *ifm1Δ* | a |
| YOL024W | *yol024wΔ* | a |
| YOL025W | *lag2Δ* | a |
| YOL027C | *mdm38Δ* | a |
| YOL028C | *yap7Δ* | a |
| YOL029C | *yol029cΔ* | a |
| YOL030W | *gas5Δ* | a |
| YOL031C | *sil1Δ* | a |
| YOL032W | *opi10Δ* | a |
| YOL033W | *mse1Δ* | a |
| YOL035C | *yol035cΔ* | a |
| YOL036W | *yol036wΔ* | a |
| YOL037C | *yol037cΔ* | a |
| YOL038C-A | *yol038c-aΔ* | a |
| YOL038W | *pre6-ts* | b |
| YOL039W | *rpp2aΔ* | a |
| YOL041C | *nop12Δ* | a |
| YOL042W | *ngl1Δ* | a |
| YOL043C | *ntg2Δ* | a |
| YOL044W | *pex15Δ* | a |
| YOL045W | *psk2Δ* | a |
| YOL046C | *yol046cΔ* | a |
| YOL047C | *yol047cΔ* | a |
| YOL048C | *rrt8Δ* | a |
| YOL048C | *rrt8Δ* | a |
| YOL049W | *gsh2Δ* | a |
| YOL050C | *yol050cΔ* | a |
| YOL051W | *gal11Δ* | a |
| YOL052C | *spe2Δ* | a |
| YOL052C-A | *ddr2Δ* | a |
| YOL053C-A | *yol053c-aΔ* | a |
| YOL053W | *aim39Δ* | a |
| YOL054W | *psh1Δ* | a |
| YOL055C | *thi20Δ* | a |
| YOL056W | *gpm3Δ* | a |
| YOL057W | *yol057wΔ* | a |
| YOL058W | *arg1Δ* | a |
| YOL059W | *gpd2Δ* | a |
| YOL060C | *mam3Δ* | a |
| YOL061W | *prs5Δ* | a |
| YOL062C | *apm4Δ* | a |
| YOL063C | *crt10Δ* | a |
| YOL064C | *met22Δ* | a |
| YOL065C | *inp54Δ* | a |
| YOL066C | *rib2-ts* | b |
| YOL067C | *rtg1Δ* | a |
| YOL068C | *hst1Δ* | a |
| YOL069W | *nuf2-ts* | b |
| YOL070C | *nba1Δ* | a |
| YOL071W | *emi5Δ* | a |
| YOL072W | *thp1Δ* | a |
| YOL073C | *yol073cΔ* | a |
| YOL075C | *yol075cΔ* | a |
| YOL076W | *mdm20Δ* | a |
| YOL077C | *brx1-ts* | b |
| YOL077W-A | *atp19Δ* | a |
| YOL079W | *yol079wΔ* | a |
| YOL080C | *rex4Δ* | a |
| YOL081W | *ira2Δ* | a |
| YOL082W | *atg19Δ* | a |
| YOL083W | *yol083wΔ* | a |
| YOL084W | *phm7Δ* | a |
| YOL085C | *yol085cΔ* | a |
| YOL086C | *adh1Δ* | a |
| YOL086W-A | *yol086w-aΔ* | a |
| YOL087C | *yol087cΔ* | a |
| YOL088C | *mpd2Δ* | a |
| YOL089C | *hal9Δ* | a |
| YOL090W | *msh2Δ* | a |
| YOL091W | *spo21Δ* | a |
| YOL092W | *yol092wΔ* | a |
| YOL093W | *trm10Δ* | a |
| YOL095C | *hmi1Δ* | a |
| YOL096C | *coq3Δ* | a |
| YOL097W-A | *yol097w-aΔ* | a |
| YOL098C | *yol098cΔ* | a |
| YOL099C | *yol099cΔ* | a |
| YOL100W | *pkh2Δ* | a |
| YOL101C | *izh4Δ* | a |
| YOL102C | *tpt1-ts* | b |
| YOL103W | *itr2Δ* | a |
| YOL104C | *ndj1Δ* | a |
| YOL105C | *wsc3Δ* | a |
| YOL106W | *yol106wΔ* | a |
| YOL107W | *yol107wΔ* | a |
| YOL108C | *ino4Δ* | a |
| YOL109W | *zeo1Δ* | a |
| YOL110W | *shr5Δ* | a |
| YOL111C | *mdy2Δ* | a |
| YOL112W | *msb4Δ* | a |
| YOL113W | *skm1Δ* | a |
| YOL114C | *yol114cΔ* | a |
| YOL115W | *pap2Δ* | a |
| YOL116W | *msn1Δ* | a |
| YOL117W | *rri2Δ* | a |
| YOL118C | *yol118cΔ* | a |
| YOL119C | *mch4Δ* | a |
| YOL121C | *rps19aΔ* | a |
| YOL122C | *smf1Δ* | a |
| YOL123W | *nab4-1* | c |
| YOL124C | *trm11Δ* | a |
| YOL125W | *trm13Δ* | a |
| YOL126C | *mdh2Δ* | a |
| YOL128C | *ygk3Δ* | a |
| YOL129W | *vps68Δ* | a |
| YOL130W | *alr1-ts* | b |
| YOL131W | *yol131wΔ* | a |
| YOL132W | *gas4Δ* | a |
| YOL136C | *pfk27Δ* | a |
| YOL137W | *bsc6Δ* | a |
| YOL138C | *rtc1Δ* | a |
| YOL140W | *arg8Δ* | a |
| YOL141W | *ppm2Δ* | a |
| YOL143C | *rib4Δ* | a |
| YOL144W | *nop8-ts* | b |
| YOL145C | *ctr9Δ* | a |
| YOL146W | *psf3-ts* | b |
| YOL147C | *pex11Δ* | a |
| YOL148C | *spt20Δ* | a |
| YOL149W | *dcp1-ts* | b |
| YOL150C | *yol150cΔ* | a |
| YOL151W | *gre2Δ* | a |
| YOL151W | *gre2Δ* | a |
| YOL152W | *fre7Δ* | a |
| YOL152W | *fre7Δ* | a |
| YOL153C | *yol153cΔ* | a |
| YOL154W | *zps1Δ* | a |
| YOL155C | *hpf1Δ* | a |
| YOL158C | *enb1Δ* | a |
| YOL159C | *yol159cΔ* | a |
| YOL159C-A | *yol159c-aΔ* | a |
| YOL160W | *yol160wΔ* | a |
| YOL162W | *yol162wΔ* | a |
| YOL163W | *yol163wΔ* | a |
| YOL164W | *bds1Δ* | a |
| YOL164W-A | *yol164w-aΔ* | a |
| YOR001W | *rrp6Δ* | a |
| YOR002W | *alg6Δ* | a |
| YOR003W | *ysp3Δ* | a |
| YOR004W | *utp23-ts* | b |
| YOR005C | *dnl4Δ* | a |
| YOR006C | *yor006cΔ* | a |
| YOR007C | *sgt2Δ* | a |
| YOR008C | *slg1Δ* | a |
| YOR008C-A | *yor008c-aΔ* | a |
| YOR009W | *tir4Δ* | a |
| YOR010C | *tir2Δ* | a |
| YOR011W | *aus1Δ* | a |
| YOR012W | *yor012wΔ* | a |
| YOR013W | *irc11Δ* | a |
| YOR014W | *rts1Δ* | a |
| YOR015W | *yor015wΔ* | a |
| YOR016C | *erp4Δ* | a |
| YOR017W | *pet127Δ* | a |
| YOR018W | *rod1Δ* | a |
| YOR019W | *yor019wΔ* | a |
| YOR020W-A | *yor020w-aΔ* | a |
| YOR021C | *yor021cΔ* | a |
| YOR022C | *yor022cΔ* | a |
| YOR023C | *ahc1Δ* | a |
| YOR024W | *yor024wΔ* | a |
| YOR025W | *hst3Δ* | a |
| YOR026W | *bub3Δ* | a |
| YOR026W | *bub3Δ* | a |
| YOR027W | *sti1Δ* | a |
| YOR028C | *cin5Δ* | a |
| YOR029W | *yor029wΔ* | a |
| YOR030W | *dfg16Δ* | a |
| YOR031W | *crs5Δ* | a |
| YOR032C | *hms1Δ* | a |
| YOR033C | *exo1Δ* | a |
| YOR034C | *akr2Δ* | a |
| YOR034C-A | *yor034c-aΔ* | a |
| YOR035C | *she4Δ* | a |
| YOR036W | *pep12Δ* | a |
| YOR037W | *cyc2Δ* | a |
| YOR038C | *hir2Δ* | a |
| YOR039W | *ckb2Δ* | a |
| YOR040W | *glo4Δ* | a |
| YOR041C | *yor041cΔ* | a |
| YOR042W | *cue5Δ* | a |
| YOR043W | *whi2Δ* | a |
| YOR044W | *irc23Δ* | a |
| YOR045W | *tom6Δ* | a |
| YOR046C | *rat8-2* | c |
| YOR047C | *std1Δ* | a |
| YOR049C | *rsb1Δ* | a |
| YOR050C | *yor050cΔ* | a |
| YOR051C | *yor051cΔ* | a |
| YOR052C | *yor052cΔ* | a |
| YOR053W | *yor053wΔ* | a |
| YOR054C | *vhs3Δ* | a |
| YOR055W | *yor055wΔ* | a |
| YOR056C | *nob1-ts* | b |
| YOR058C | *ase1Δ* | a |
| YOR059C | *yor059cΔ* | a |
| YOR060C | *yor060c-ts* | b |
| YOR061W | *cka2Δ* | a |
| YOR062C | *yor062cΔ* | a |
| YOR063W | *rpl3-ts* | b |
| YOR064C | *yng1Δ* | a |
| YOR065W | *cyt1Δ* | a |
| YOR066W | *msa1Δ* | a |
| YOR067C | *alg8Δ* | a |
| YOR068C | *vam10Δ* | a |
| YOR069W | *vps5Δ* | a |
| YOR069W | *vps5Δ* | a |
| YOR070C | *gyp1Δ* | a |
| YOR071C | *nrt1Δ* | a |
| YOR072W | *yor072wΔ* | a |
| YOR073W | *sgo1Δ* | a |
| YOR076C | *ski7Δ* | a |
| YOR078W | *bud21Δ* | a |
| YOR079C | *atx2Δ* | a |
| YOR080W | *dia2Δ* | a |
| YOR081C | *tgl5Δ* | a |
| YOR082C | *yor082cΔ* | a |
| YOR083W | *whi5Δ* | a |
| YOR084W | *lpx1Δ* | a |
| YOR085W | *ost3Δ* | a |
| YOR086C | *tcb1Δ* | a |
| YOR087W | *yvc1Δ* | a |
| YOR087W | *yvc1Δ* | a |
| YOR088W | *yor088wΔ* | a |
| YOR089C | *vps21Δ* | a |
| YOR090C | *ptc5Δ* | a |
| YOR091W | *tma46Δ* | a |
| YOR092W | *ecm3Δ* | a |
| YOR093C | *yor093cΔ* | a |
| YOR094W | *arf3Δ* | a |
| YOR096W | *rps7aΔ* | a |
| YOR097C | *yor097cΔ* | a |
| YOR099W | *ktr1Δ* | a |
| YOR100C | *crc1Δ* | a |
| YOR101W | *ras1Δ* | a |
| YOR103C | *ost2-ts* | b |
| YOR104W | *pin2Δ* | a |
| YOR105W | *yor105wΔ* | a |
| YOR106W | *vam3Δ* | a |
| YOR107W | *rgs2Δ* | a |
| YOR108W | *leu9Δ* | a |
| YOR109W | *inp53Δ* | a |
| YOR111W | *yor111wΔ* | a |
| YOR112W | *cex1Δ* | a |
| YOR113W | *azf1Δ* | a |
| YOR114W | *yor114wΔ* | a |
| YOR115C | *trs33Δ* | a |
| YOR118W | *rtc5Δ* | a |
| YOR120W | *gcy1Δ* | a |
| YOR121C | *yor121cΔ* | a |
| YOR123C | *leo1Δ* | a |
| YOR124C | *ubp2Δ* | a |
| YOR125C | *cat5Δ* | a |
| YOR126C | *iah1Δ* | a |
| YOR127W | *rga1Δ* | a |
| YOR128C | *ade2Δ* | a |
| YOR129C | *afi1Δ* | a |
| YOR130C | *ort1Δ* | a |
| YOR131C | *yor131cΔ* | a |
| YOR132W | *vps17Δ* | a |
| YOR133W | *eft1Δ* | a |
| YOR134W | *bag7Δ* | a |
| YOR135C | *irc14Δ* | a |
| YOR136W | *idh2Δ* | a |
| YOR137C | *sia1Δ* | a |
| YOR138C | *rup1Δ* | a |
| YOR139C | *yor139cΔ* | a |
| YOR140W | *sfl1Δ* | a |
| YOR141C | *arp8Δ* | a |
| YOR142W | *lsc1Δ* | a |
| YOR143C | *thi80-ts* | b |
| YOR144C | *elg1Δ* | a |
| YOR147W | *mdm32Δ* | a |
| YOR148C | *spp2-ts* | b |
| YOR150W | *mrpl23Δ* | a |
| YOR152C | *yor152cΔ* | a |
| YOR153W | *pdr5Δ* | a |
| YOR154W | *slp1Δ* | a |
| YOR155C | *isn1Δ* | a |
| YOR156C | *nfi1Δ* | a |
| YOR158W | *pet123Δ* | a |
| YOR160W | *mtr10-ts* | b |
| YOR161C | *pns1Δ* | a |
| YOR161C-C | *yor161c-cΔ* | a |
| YOR162C | *yrr1Δ* | a |
| YOR163W | *ddp1Δ* | a |
| YOR164C | *get4Δ* | a |
| YOR165W | *sey1Δ* | a |
| YOR166C | *swt1Δ* | a |
| YOR167C | *rps28aΔ* | a |
| YOR168W | *gln4-ts* | b |
| YOR170W | *yor170wΔ* | a |
| YOR171C | *lcb4Δ* | a |
| YOR172W | *yrm1Δ* | a |
| YOR173W | *dcs2Δ* | a |
| YOR175C | *ale1Δ* | a |
| YOR176W | *hem15-ts* | b |
| YOR177C | *mpc54Δ* | a |
| YOR178C | *gac1Δ* | a |
| YOR179C | *syc1Δ* | a |
| YOR180C | *dci1Δ* | a |
| YOR182C | *rps30bΔ* | a |
| YOR183W | *fyv12Δ* | a |
| YOR184W | *ser1Δ* | a |
| YOR185C | *gsp2Δ* | a |
| YOR186W | *yor186wΔ* | a |
| YOR187W | *tuf1Δ* | a |
| YOR188W | *msb1Δ* | a |
| YOR189W | *ies4Δ* | a |
| YOR190W | *spr1Δ* | a |
| YOR191W | *uls1Δ* | a |
| YOR192C | *thi72Δ* | a |
| YOR193W | *pex27Δ* | a |
| YOR194C | *toa1-ts* | b |
| YOR195W | *slk19Δ* | a |
| YOR196C | *lip5Δ* | a |
| YOR197W | *mca1Δ* | a |
| YOR198C | *bfr1Δ* | a |
| YOR199W | *yor199wΔ* | a |
| YOR200W | *yor200wΔ* | a |
| YOR201C | *mrm1Δ* | a |
| YOR202W | *his3Δ* | a |
| YOR205C | *yor205cΔ* | a |
| YOR206W | *noc2-ts* | b |
| YOR208W | *ptp2Δ* | a |
| YOR209C | *npt1Δ* | a |
| YOR211C | *mgm1Δ* | a |
| YOR212W | *ste4Δ* | a |
| YOR213C | *sas5Δ* | a |
| YOR214C | *yor214cΔ* | a |
| YOR215C | *aim41Δ* | a |
| YOR216C | *rud3Δ* | a |
| YOR219C | *ste13Δ* | a |
| YOR220W | *rcn2Δ* | a |
| YOR221C | *mct1Δ* | a |
| YOR222W | *odc2Δ* | a |
| YOR223W | *yor223wΔ* | a |
| YOR224C | *rpb8-ts* | b |
| YOR225W | *yor225wΔ* | a |
| YOR226C | *isu2Δ* | a |
| YOR227W | *her1Δ* | a |
| YOR228C | *yor228cΔ* | a |
| YOR229W | *wtm2Δ* | a |
| YOR230W | *wtm1Δ* | a |
| YOR231W | *mkk1Δ* | a |
| YOR233W | *kin4Δ* | a |
| YOR234C | *rpl33bΔ* | a |
| YOR235W | *irc13Δ* | a |
| YOR237W | *hes1Δ* | a |
| YOR238W | *yor238wΔ* | a |
| YOR239W | *abp140Δ* | a |
| YOR239W | *abp140Δ* | a |
| YOR240W | *yor240wΔ* | a |
| YOR241W | *met7Δ* | a |
| YOR242C | *ssp2Δ* | a |
| YOR243C | *pus7Δ* | a |
| YOR245C | *dga1Δ* | a |
| YOR246C | *yor246cΔ* | a |
| YOR247W | *srl1Δ* | a |
| YOR248W | *yor248wΔ* | a |
| YOR250C | *clp1-ts* | b |
| YOR251C | *tum1Δ* | a |
| YOR252W | *tma16Δ* | a |
| YOR253W | *nat5Δ* | a |
| YOR255W | *osw1Δ* | a |
| YOR258W | *hnt3Δ* | a |
| YOR260W | *gcd1-ts* | b |
| YOR261C | *rpn8-ts* | b |
| YOR262W | *yor262w-ts* | b |
| YOR263C | *yor263cΔ* | a |
| YOR264W | *dse3Δ* | a |
| YOR265W | *rbl2Δ* | a |
| YOR266W | *pnt1Δ* | a |
| YOR267C | *hrk1Δ* | a |
| YOR268C | *yor268cΔ* | a |
| YOR269W | *pac1Δ* | a |
| YOR270C | *vph1Δ* | a |
| YOR271C | *fsf1Δ* | a |
| YOR273C | *tpo4Δ* | a |
| YOR274W | *mod5Δ* | a |
| YOR275C | *rim20Δ* | a |
| YOR276W | *caf20Δ* | a |
| YOR277C | *yor277cΔ* | a |
| YOR279C | *rfm1Δ* | a |
| YOR280C | *fsh3Δ* | a |
| YOR283W | *yor283wΔ* | a |
| YOR284W | *hua2Δ* | a |
| YOR285W | *yor285wΔ* | a |
| YOR286W | *aim42Δ* | a |
| YOR288C | *mpd1Δ* | a |
| YOR289W | *yor289wΔ* | a |
| YOR290C | *snf2Δ* | a |
| YOR291W | *ypk9Δ* | a |
| YOR292C | *yor292cΔ* | a |
| YOR293C-A | *yor293c-aΔ* | a |
| YOR293W | *rps10aΔ* | a |
| YOR295W | *uaf30Δ* | a |
| YOR296W | *yor296wΔ* | a |
| YOR297C | *tim18Δ* | a |
| YOR298C-A | *mbf1Δ* | a |
| YOR298C-A | *mbf1Δ* | a |
| YOR298C-A | *mbf1Δ* | a |
| YOR298W | *mum3Δ* | a |
| YOR299W | *bud7Δ* | a |
| YOR300W | *yor300wΔ* | a |
| YOR300W | *yor300wΔ* | a |
| YOR301W | *rax1Δ* | a |
| YOR302W | *yor302wΔ* | a |
| YOR302W | *yor302wΔ* | a |
| YOR303W | *cpa1Δ* | a |
| YOR303W | *cpa1Δ* | a |
| YOR304C-A | *yor304c-aΔ* | a |
| YOR304W | *isw2Δ* | a |
| YOR305W | *yor305wΔ* | a |
| YOR306C | *mch5Δ* | a |
| YOR306C | *mch5Δ* | a |
| YOR307C | *sly41Δ* | a |
| YOR308C | *snu66Δ* | a |
| YOR309C | *yor309cΔ* | a |
| YOR309C | *yor309cΔ* | a |
| YOR310C | *nop58-ts* | b |
| YOR311C | *dgk1Δ* | a |
| YOR312C | *rpl20bΔ* | a |
| YOR313C | *sps4Δ* | a |
| YOR314W | *yor314wΔ* | a |
| YOR315W | *sfg1Δ* | a |
| YOR316C | *cot1Δ* | a |
| YOR316C-A | *yor316c-aΔ* | a |
| YOR317W | *faa1Δ* | a |
| YOR318C | *yor318cΔ* | a |
| YOR320C | *gnt1Δ* | a |
| YOR321W | *pmt3Δ* | a |
| YOR322C | *ldb19Δ* | a |
| YOR323C | *pro2Δ* | a |
| YOR324C | *frt1Δ* | a |
| YOR325W | *yor325wΔ* | a |
| YOR327C | *snc2Δ* | a |
| YOR328W | *pdr10Δ* | a |
| YOR330C | *mip1Δ* | a |
| YOR331C | *yor331cΔ* | a |
| YOR332W | *vma4Δ* | a |
| YOR333C | *yor333cΔ* | a |
| YOR334W | *mrs2Δ* | a |
| YOR337W | *tea1Δ* | a |
| YOR338W | *yor338wΔ* | a |
| YOR339C | *ubc11Δ* | a |
| YOR342C | *yor342cΔ* | a |
| YOR343C | *yor343cΔ* | a |
| YOR344C | *tye7Δ* | a |
| YOR345C | *yor345cΔ* | a |
| YOR346W | *rev1Δ* | a |
| YOR347C | *pyk2Δ* | a |
| YOR348C | *put4Δ* | a |
| YOR349W | *cin1Δ* | a |
| YOR350C | *mne1Δ* | a |
| YOR351C | *mek1Δ* | a |
| YOR352W | *yor352wΔ* | a |
| YOR353C | *sog2-ts* | b |
| YOR354C | *msc6Δ* | a |
| YOR355W | *gds1Δ* | a |
| YOR356W | *yor356wΔ* | a |
| YOR357C | *snx3Δ* | a |
| YOR358W | *hap5Δ* | a |
| YOR359W | *vts1Δ* | a |
| YOR360C | *pde2Δ* | a |
| YOR362C | *pre10-ts* | b |
| YOR363C | *pip2Δ* | a |
| YOR364W | *yor364wΔ* | a |
| YOR364W | *yor364wΔ* | a |
| YOR365C | *yor365cΔ* | a |
| YOR366W | *yor366wΔ* | a |
| YOR367W | *scp1Δ* | a |
| YOR368W | *rad17Δ* | a |
| YOR369C | *rps12Δ* | a |
| YOR371C | *gpb1Δ* | a |
| YOR374W | *ald4Δ* | a |
| YOR375C | *gdh1Δ* | a |
| YOR376W | *yor376wΔ* | a |
| YOR376W-A | *yor376w-aΔ* | a |
| YOR377W | *atf1Δ* | a |
| YOR378W | *yor378wΔ* | a |
| YOR379C | *yor379cΔ* | a |
| YOR380W | *rdr1Δ* | a |
| YOR381W | *fre3Δ* | a |
| YOR382W | *fit2Δ* | a |
| YOR383C | *fit3Δ* | a |
| YOR384W | *fre5Δ* | a |
| YOR385W | *yor385wΔ* | a |
| YOR386W | *phr1Δ* | a |
| YPL001W | *hat1Δ* | a |
| YPL002C | *snf8Δ* | a |
| YPL003W | *ula1Δ* | a |
| YPL004C | *lsp1Δ* | a |
| YPL005W | *aep3Δ* | a |
| YPL006W | *ncr1Δ* | a |
| YPL007C | *tfc8-ts* | b |
| YPL008W | *chl1Δ* | a |
| YPL009C | *ypl009cΔ* | a |
| YPL013C | *mrps16Δ* | a |
| YPL014W | *ypl014wΔ* | a |
| YPL015C | *hst2Δ* | a |
| YPL017C | *irc15Δ* | a |
| YPL018W | *ctf19Δ* | a |
| YPL019C | *vtc3Δ* | a |
| YPL021W | *ecm23Δ* | a |
| YPL022W | *rad1Δ* | a |
| YPL023C | *met12Δ* | a |
| YPL024W | *rmi1Δ* | a |
| YPL025C | *ypl025cΔ* | a |
| YPL026C | *sks1Δ* | a |
| YPL027W | *sma1Δ* | a |
| YPL029W | *suv3Δ* | a |
| YPL030W | *trm44Δ* | a |
| YPL031C | *pho85Δ* | a |
| YPL032C | *svl3Δ* | a |
| YPL033C | *srl4Δ* | a |
| YPL034W | *ypl034wΔ* | a |
| YPL035C | *ypl035cΔ* | a |
| YPL036W | *pma2Δ* | a |
| YPL037C | *egd1Δ* | a |
| YPL038W | *met31Δ* | a |
| YPL038W-A | *ypl038w-aΔ* | a |
| YPL039W | *ypl039wΔ* | a |
| YPL040C | *ism1Δ* | a |
| YPL041C | *ypl041cΔ* | a |
| YPL042C | *ssn3Δ* | a |
| YPL045W | *vps16Δ* | a |
| YPL046C | *elc1Δ* | a |
| YPL047W | *sgf11Δ* | a |
| YPL048W | *cam1Δ* | a |
| YPL049C | *dig1Δ* | a |
| YPL050C | *mnn9Δ* | a |
| YPL051W | *arl3Δ* | a |
| YPL052W | *oaz1Δ* | a |
| YPL053C | *ktr6Δ* | a |
| YPL054W | *lee1Δ* | a |
| YPL055C | *lge1Δ* | a |
| YPL056C | *ypl056cΔ* | a |
| YPL057C | *sur1Δ* | a |
| YPL058C | *pdr12Δ* | a |
| YPL059W | *grx5Δ* | a |
| YPL060W | *lpe10Δ* | a |
| YPL061W | *ald6Δ* | a |
| YPL062W | *ypl062wΔ* | a |
| YPL063W | *tim50-ts* | b |
| YPL064C | *cwc27Δ* | a |
| YPL065W | *vps28Δ* | a |
| YPL066W | *ypl066wΔ* | a |
| YPL067C | *ypl067cΔ* | a |
| YPL068C | *ypl068cΔ* | a |
| YPL069C | *bts1Δ* | a |
| YPL070W | *muk1Δ* | a |
| YPL071C | *ypl071cΔ* | a |
| YPL072W | *ubp16Δ* | a |
| YPL073C | *ypl073cΔ* | a |
| YPL074W | *yta6Δ* | a |
| YPL075W | *gcr1Δ* | a |
| YPL077C | *ypl077cΔ* | a |
| YPL078C | *atp4Δ* | a |
| YPL078C | *atp4Δ* | a |
| YPL079W | *rpl21bΔ* | a |
| YPL080C | *ypl080cΔ* | a |
| YPL081W | *rps9aΔ* | a |
| YPL084W | *bro1Δ* | a |
| YPL086C | *elp3Δ* | a |
| YPL087W | *ydc1Δ* | a |
| YPL088W | *ypl088wΔ* | a |
| YPL089C | *rlm1Δ* | a |
| YPL090C | *rps6aΔ* | a |
| YPL091W | *glr1Δ* | a |
| YPL092W | *ssu1Δ* | a |
| YPL095C | *eeb1Δ* | a |
| YPL096C-A | *eri1Δ* | a |
| YPL096W | *png1Δ* | a |
| YPL097W | *msy1Δ* | a |
| YPL098C | *mgr2Δ* | a |
| YPL099C | *aim43Δ* | a |
| YPL100W | *atg21Δ* | a |
| YPL101W | *elp4Δ* | a |
| YPL102C | *ypl102cΔ* | a |
| YPL103C | *fmp30Δ* | a |
| YPL104W | *msd1Δ* | a |
| YPL105C | *syh1Δ* | a |
| YPL106C | *sse1Δ* | a |
| YPL107W | *ypl107wΔ* | a |
| YPL108W | *ypl108wΔ* | a |
| YPL109C | *ypl109cΔ* | a |
| YPL110C | *gde1Δ* | a |
| YPL111W | *car1Δ* | a |
| YPL112C | *pex25Δ* | a |
| YPL113C | *ypl113cΔ* | a |
| YPL114W | *ypl114wΔ* | a |
| YPL115C | *bem3Δ* | a |
| YPL116W | *hos3Δ* | a |
| YPL118W | *mrp51Δ* | a |
| YPL119C | *dbp1Δ* | a |
| YPL119C-A | *ypl119c-aΔ* | a |
| YPL120W | *vps30Δ* | a |
| YPL121C | *mei5Δ* | a |
| YPL123C | *rny1Δ* | a |
| YPL125W | *kap120Δ* | a |
| YPL127C | *hho1Δ* | a |
| YPL128C | *tbf1-ts* | b |
| YPL129W | *taf14Δ* | a |
| YPL130W | *spo19Δ* | a |
| YPL131W | *rpl5-ts* | b |
| YPL132W | *cox11Δ* | a |
| YPL133C | *rds2Δ* | a |
| YPL134C | *odc1Δ* | a |
| YPL135W | *isu1Δ* | a |
| YPL136W | *ypl136wΔ* | a |
| YPL137C | *gip3Δ* | a |
| YPL138C | *spp1Δ* | a |
| YPL139C | *ume1Δ* | a |
| YPL140C | *mkk2Δ* | a |
| YPL141C | *ypl141cΔ* | a |
| YPL144W | *poc4Δ* | a |
| YPL145C | *kes1Δ* | a |
| YPL147W | *pxa1Δ* | a |
| YPL148C | *ppt2Δ* | a |
| YPL149W | *atg5Δ* | a |
| YPL150W | *ypl150wΔ* | a |
| YPL151C | *prp46-ts* | b |
| YPL152W | *rrd2Δ* | a |
| YPL152W-A | *ypl152w-aΔ* | a |
| YPL154C | *pep4Δ* | a |
| YPL155C | *kip2Δ* | a |
| YPL156C | *prm4Δ* | a |
| YPL157W | *tgs1Δ* | a |
| YPL158C | *aim44Δ* | a |
| YPL159C | *pet20Δ* | a |
| YPL161C | *bem4Δ* | a |
| YPL162C | *ypl162cΔ* | a |
| YPL163C | *svs1Δ* | a |
| YPL164C | *mlh3Δ* | a |
| YPL165C | *set6Δ* | a |
| YPL165C | *set6Δ* | a |
| YPL166W | *atg29Δ* | a |
| YPL167C | *rev3Δ* | a |
| YPL168W | *ypl168wΔ* | a |
| YPL170W | *dap1Δ* | a |
| YPL171C | *oye3Δ* | a |
| YPL172C | *cox10Δ* | a |
| YPL173W | *mrpl40Δ* | a |
| YPL174C | *nip100Δ* | a |
| YPL176C | *tre1Δ* | a |
| YPL177C | *cup9Δ* | a |
| YPL178W | *cbc2Δ* | a |
| YPL179W | *ppq1Δ* | a |
| YPL180W | *tco89Δ* | a |
| YPL181W | *cti6Δ* | a |
| YPL182C | *ypl182cΔ* | a |
| YPL183C | *rtt10Δ* | a |
| YPL183W | *ypl183wΔ* | a |
| YPL183W-A | *rtc6Δ* | a |
| YPL184C | *mrn1Δ* | a |
| YPL185W | *ypl185wΔ* | a |
| YPL186C | *uip4Δ* | a |
| YPL187W | *mf(alpha)1Δ* | a |
| YPL188W | *pos5Δ* | a |
| YPL189C-A | *coa2Δ* | a |
| YPL189W | *gup2Δ* | a |
| YPL189W | *gup2Δ* | a |
| YPL191C | *ypl191cΔ* | a |
| YPL192C | *prm3Δ* | a |
| YPL193W | *rsa1Δ* | a |
| YPL194W | *ddc1Δ* | a |
| YPL194W | *ddc1Δ* | a |
| YPL195W | *apl5Δ* | a |
| YPL196W | *oxr1Δ* | a |
| YPL197C | *ypl197cΔ* | a |
| YPL198W | *rpl7bΔ* | a |
| YPL199C | *ypl199cΔ* | a |
| YPL200W | *csm4Δ* | a |
| YPL201C | *yig1Δ* | a |
| YPL202C | *aft2Δ* | a |
| YPL203W | *tpk2Δ* | a |
| YPL204W | *hrr25-ts* | b |
| YPL205C | *ypl205cΔ* | a |
| YPL206C | *pgc1Δ* | a |
| YPL207W | *tyw1Δ* | a |
| YPL208W | *rkm1Δ* | a |
| YPL212C | *pus1Δ* | a |
| YPL213W | *lea1Δ* | a |
| YPL214C | *thi6Δ* | a |
| YPL215W | *cbp3Δ* | a |
| YPL216W | *ypl216wΔ* | a |
| YPL219W | *pcl8Δ* | a |
| YPL220W | *rpl1aΔ* | a |
| YPL221W | *flc1Δ* | a |
| YPL222W | *fmp40Δ* | a |
| YPL223C | *gre1Δ* | a |
| YPL224C | *mmt2Δ* | a |
| YPL225W | *ypl225wΔ* | a |
| YPL226W | *new1Δ* | a |
| YPL227C | *alg5Δ* | a |
| YPL229W | *ypl229wΔ* | a |
| YPL230W | *usv1Δ* | a |
| YPL232W | *sso1Δ* | a |
| YPL234C | *tfp3Δ* | a |
| YPL235W | *rvb2-ts* | b |
| YPL236C | *ypl236cΔ* | a |
| YPL239W | *yar1Δ* | a |
| YPL240C | *hsp82Δ* | a |
| YPL241C | *cin2Δ* | a |
| YPL244C | *hut1Δ* | a |
| YPL245W | *ypl245wΔ* | a |
| YPL246C | *rbd2Δ* | a |
| YPL247C | *ypl247cΔ* | a |
| YPL248C | *gal4Δ* | a |
| YPL249C | *gyp5Δ* | a |
| YPL249C-A | *rpl36bΔ* | a |
| YPL250C | *icy2Δ* | a |
| YPL253C | *vik1Δ* | a |
| YPL254W | *hfi1Δ* | a |
| YPL256C | *cln2Δ* | a |
| YPL257W | *ypl257wΔ* | a |
| YPL258C | *thi21Δ* | a |
| YPL259C | *apm1Δ* | a |
| YPL260W | *ypl260wΔ* | a |
| YPL261C | *ypl261cΔ* | a |
| YPL262W | *fum1Δ* | a |
| YPL263C | *kel3Δ* | a |
| YPL264C | *ypl264cΔ* | a |
| YPL265W | *dip5Δ* | a |
| YPL267W | *acm1Δ* | a |
| YPL268W | *plc1Δ* | a |
| YPL269W | *kar9Δ* | a |
| YPL270W | *mdl2Δ* | a |
| YPL271W | *atp15Δ* | a |
| YPL272C | *ypl272cΔ* | a |
| YPL273W | *sam4Δ* | a |
| YPL274W | *sam3Δ* | a |
| YPL277C | *ypl277cΔ* | a |
| YPR001W | *cit3Δ* | a |
| YPR002W | *pdh1Δ* | a |
| YPR003C | *ypr003cΔ* | a |
| YPR004C | *aim45Δ* | a |
| YPR005C | *hal1Δ* | a |
| YPR006C | *icl2Δ* | a |
| YPR007C | *rec8Δ* | a |
| YPR008W | *haa1Δ* | a |
| YPR009W | *sut2Δ* | a |
| YPR011C | *ypr011cΔ* | a |
| YPR012W | *ypr012wΔ* | a |
| YPR013C | *ypr013cΔ* | a |
| YPR014C | *ypr014cΔ* | a |
| YPR015C | *ypr015cΔ* | a |
| YPR016C | *tif6-ts* | b |
| YPR017C | *dss4Δ* | a |
| YPR018W | *rlf2Δ* | a |
| YPR019W | *mcm4-ts* | b |
| YPR020W | *atp20Δ* | a |
| YPR021C | *agc1Δ* | a |
| YPR022C | *ypr022cΔ* | a |
| YPR023C | *eaf3Δ* | a |
| YPR024W | *yme1Δ* | a |
| YPR026W | *ath1Δ* | a |
| YPR027C | *ypr027cΔ* | a |
| YPR028W | *yop1Δ* | a |
| YPR029C | *apl4Δ* | a |
| YPR030W | *csr2Δ* | a |
| YPR031W | *nto1Δ* | a |
| YPR032W | *sro7Δ* | a |
| YPR035W | *gln1-ts* | b |
| YPR036W | *vma13Δ* | a |
| YPR037C | *erv2Δ* | a |
| YPR038W | *irc16Δ* | a |
| YPR039W | *ypr039wΔ* | a |
| YPR040W | *tip41Δ* | a |
| YPR042C | *puf2Δ* | a |
| YPR043W | *rpl43aΔ* | a |
| YPR044C | *opi11Δ* | a |
| YPR045C | *ypr045cΔ* | a |
| YPR046W | *mcm16Δ* | a |
| YPR047W | *msf1Δ* | a |
| YPR049C | *atg11Δ* | a |
| YPR050C | *ypr050cΔ* | a |
| YPR051W | *mak3Δ* | a |
| YPR052C | *nhp6aΔ* | a |
| YPR053C | *ypr053cΔ* | a |
| YPR054W | *smk1Δ* | a |
| YPR057W | *brr1Δ* | a |
| YPR058W | *ymc1Δ* | a |
| YPR059C | *ypr059cΔ* | a |
| YPR060C | *aro7Δ* | a |
| YPR061C | *jid1Δ* | a |
| YPR062W | *fcy1Δ* | a |
| YPR063C | *ypr063cΔ* | a |
| YPR064W | *ypr064wΔ* | a |
| YPR065W | *rox1Δ* | a |
| YPR066W | *uba3Δ* | a |
| YPR067W | *isa2Δ* | a |
| YPR068C | *hos1Δ* | a |
| YPR069C | *spe3Δ* | a |
| YPR070W | *med1Δ* | a |
| YPR071W | *ypr071wΔ* | a |
| YPR072W | *not5Δ* | a |
| YPR073C | *ltp1Δ* | a |
| YPR074C | *tkl1Δ* | a |
| YPR075C | *opy2Δ* | a |
| YPR076W | *ypr076wΔ* | a |
| YPR077C | *ypr077cΔ* | a |
| YPR078C | *ypr078cΔ* | a |
| YPR079W | *mrl1Δ* | a |
| YPR082C | *dib1-ts* | b |
| YPR083W | *mdm36Δ* | a |
| YPR084W | *ypr084wΔ* | a |
| YPR085C | *asa1-ts* | b |
| YPR086W | *sua7-ts* | b |
| YPR087W | *vps69Δ* | a |
| YPR088C | *srp54-ts* | b |
| YPR089W | *ypr089wΔ* | a |
| YPR089W | *ypr089wΔ* | a |
| YPR090W | *ypr090wΔ* | a |
| YPR091C | *ypr091cΔ* | a |
| YPR092W | *ypr092wΔ* | a |
| YPR093C | *asr1Δ* | a |
| YPR095C | *syt1Δ* | a |
| YPR096C | *ypr096cΔ* | a |
| YPR097W | *ypr097wΔ* | a |
| YPR098C | *ypr098cΔ* | a |
| YPR098C | *ypr098cΔ* | a |
| YPR099C | *ypr099cΔ* | a |
| YPR100W | *mrpl51Δ* | a |
| YPR101W | *snt309Δ* | a |
| YPR106W | *isr1Δ* | a |
| YPR107C | *yth1-ts* | b |
| YPR108W-A | *ypr108w-aΔ* | a |
| YPR109W | *ypr109wΔ* | a |
| YPR111W | *dbf20Δ* | a |
| YPR114W | *ypr114wΔ* | a |
| YPR115W | *ypr115wΔ* | a |
| YPR116W | *ypr116wΔ* | a |
| YPR117W | *ypr117wΔ* | a |
| YPR118W | *mri1Δ* | a |
| YPR119W | *clb2Δ* | a |
| YPR120C | *clb5Δ* | a |
| YPR121W | *thi22Δ* | a |
| YPR122W | *axl1Δ* | a |
| YPR123C | *ypr123cΔ* | a |
| YPR124W | *ctr1Δ* | a |
| YPR125W | *ylh47Δ* | a |
| YPR126C | *ypr126cΔ* | a |
| YPR127W | *ypr127wΔ* | a |
| YPR128C | *ant1Δ* | a |
| YPR129W | *scd6Δ* | a |
| YPR130C | *ypr130cΔ* | a |
| YPR131C | *nat3Δ* | a |
| YPR132W | *rps23bΔ* | a |
| YPR133C | *spn1-ts* | b |
| YPR133W-A | *tom5Δ* | a |
| YPR134W | *mss18Δ* | a |
| YPR135W | *ctf4Δ* | a |
| YPR137W | *rrp9-ts* | b |
| YPR138C | *mep3Δ* | a |
| YPR139C | *vps66Δ* | a |
| YPR140W | *taz1Δ* | a |
| YPR141C | *kar3Δ* | a |
| YPR141C | *kar3Δ* | a |
| YPR144C | *noc4-ts* | b |
| YPR145W | *asn1Δ* | a |
| YPR146C | *ypr146cΔ* | a |
| YPR147C | *ypr147cΔ* | a |
| YPR148C | *ypr148cΔ* | a |
| YPR149W | *nce102Δ* | a |
| YPR150W | *ypr150wΔ* | a |
| YPR151C | *sue1Δ* | a |
| YPR152C | *urn1Δ* | a |
| YPR153W | *ypr153wΔ* | a |
| YPR154W | *pin3Δ* | a |
| YPR155C | *nca2Δ* | a |
| YPR156C | *tpo3Δ* | a |
| YPR157W | *ypr157wΔ* | a |
| YPR158W | *cur1Δ* | a |
| YPR159C-A | *ypr159c-aΔ* | a |
| YPR159W | *kre6Δ* | a |
| YPR160W | *gph1Δ* | a |
| YPR161C | *sgv1-ts* | b |
| YPR161C | *sgv1-ts* | b |
| YPR162C | *orc4-ts* | b |
| YPR163C | *tif3Δ* | a |
| YPR164W | *mms1Δ* | a |
| YPR166C | *mrp2Δ* | a |
| YPR167C | *met16Δ* | a |
| YPR170C | *ypr170cΔ* | a |
| YPR171W | *bsp1Δ* | a |
| YPR172W | *ypr172wΔ* | a |
| YPR173C | *vps4Δ* | a |
| YPR174C | *ypr174cΔ* | a |
| YPR179C | *hda3Δ* | a |
| YPR180W | *aos1-ts* | b |
| YPR184W | *gdb1Δ* | a |
| YPR185W | *atg13Δ* | a |
| YPR188C | *mlc2Δ* | a |
| YPR189W | *ski3Δ* | a |
| YPR190C | *rpc82-ts* | b |
| YPR191W | *qcr2Δ* | a |
| YPR192W | *aqy1Δ* | a |
| YPR193C | *hpa2Δ* | a |
| YPR194C | *opt2Δ* | a |
| YPR195C | *ypr195cΔ* | a |
| YPR196W | *ypr196wΔ* | a |
| YPR197C | *ypr197cΔ* | a |
| YPR198W | *sge1Δ* | a |
| YPR199C | *arr1Δ* | a |
| YPR200C | *arr2Δ* | a |
| YPR201W | *arr3Δ* | a |
