# Supplementary material for: A Quantitative, High-Throughput Reverse Genetic Screen Reveals Novel Connections between Pre–mRNA Splicing and 5′ and 3′ End Transcript Determinants
Source: PLoS Genet. 2012 Mar 29;8(3):e1002530. doi: 10.1371/journal.pgen.1002530 (PMC3315463; doi:10.1371/journal.pgen.1002530)
Supplement: Table S4 — Forward and Reverse primer sequences used for QPCR for each target RNA. The list of forward and reverse primers used in this study. (DOCX) [file pgen.1002530.s013.docx]

| **Target** | **Forward primer** | **Reverse primer** |
| --- | --- | --- |
| U3 precursor (snR17a) | TAT GTA ATA TAC CCC AAA CAT TTT ACC CAC | AAA CTC TAG TAC CTA AGA CTT TTA GAT GCT |
| Tef5 pre-mRNA | GAT AGC ACA GAG CAG AGT ATC ATT A | CTG GAG AAT TCT GGG TAA GCA GAT T |
| Rpl31b pre-mRNA | ATT TCT CTG TGT TCT GCG ATC GAT | AGC GCC ATT ATA GTG TAA ACG TGA G |
| Ubc13 pre-mRNA | GAG CCG TCA CAC TCT ACA CTG TTT C | TTG CGT TAG TAT GTA AGT TCT AGC CA |
| Tub3 pre-mRNA | GCA AAT AAT TTG ACT TGT ACC AGC A | AGG TCA ATT ATG AGC TTT TTC GTC C |
| Yra1 pre-mRNA | ACA TTT TTA AGC TGG CGT ATT GTG T | TAG GAA CTA ACT ATC ACG GTC GAG G |
| Rec107 pre-mRNA | TCG CTA AAC AAG TTT AAC CAG CTA CT | AAA CTT GAG GGT ATG CAC TGT GTT |
| U1 snRNA | AAG TCC TAC TGA TCA AAC ATG CGC | GGA GTT TGC ATC AAT GAC TTC AAT G |
| Scr1 total | CAG GAG GCG TGA GGA ATC CGT CTC T | AAG GAC CCA GAA CTA CCT TGC CGC A |
| Tef5 total | CCA TTG TCA CTC TAG ATG TCA AGC C | GAT ACC GAA ACC AAT TGG GAT AAA T |
| Tub1 total | AGA AAC TTG GAT ATC CCA AGA CCA A | GCA ATT AGG TTG TTT AAG TTT GCA A |
| Faa1 total | TCT GCC CTA TGC TTA TTG GTT ACG | TAA CAC AAC CTG TTA GGT CAC CAG C |
| Srb2 total | CGC GGA TGT CTC CAA GCT CA | CGC GGA TGT CTC CAA GCT CA |
| Mud1 total | AAT AAG ACG CCT GAA GCA TAA GCT | CTA TCA ATC TCA GCC TCC TCT ACC TT |
